# From Kinase Inhibitors to Multitarget Ligands as Powerful Drug Leads for Alzheimer's Disease using Protein‐Templated Synthesis

**DOI:** 10.1002/anie.202106295

**Published:** 2021-07-26

**Authors:** Vanesa Nozal, Alfonso García‐Rubia, Eva P. Cuevas, Concepción Pérez, Carlota Tosat‐Bitrián, Fernando Bartolomé, Eva Carro, David Ramírez, Valle Palomo, Ana Martínez

**Affiliations:** ^1^ Structural and Chemical Biology Department Centro de Investigaciones Biológicas-CSIC Ramiro de Maeztu 9 28040 Madrid Spain; ^2^ Centro de Investigación Biomédica en Red de Enfermedades Neurodegenerativas (CIBERNED) Instituto de Salud Carlos III 28031 Madrid Spain; ^3^ Instituto de Química Médica-CSIC) Juan de la Cierva 3 28006 Madrid Spain; ^4^ Hospital Universitario 12 de Octubre Research Institute (imas12) Group of Neurodegenerative Diseases Juan de la Cierva 3 28006 Madrid Spain; ^5^ Instituto de Ciencias Biomédicas Universidad Autónoma de Chile Llano Subercaseaux 2801—piso 6 Santiago Chile

**Keywords:** Alzheimer's disease, BACE1, in situ click chemistry, multitarget directed ligands, protein kinase inhibitors

## Abstract

Multitarget directed ligands (MTDLs) are arising as promising tools to tackle complex diseases. The main goal of this work is to create powerful modulating agents for neurodegenerative disorders. To achieve this aim, we have combined fragments that inhibit key protein kinases involved in the main pathomolecular pathways of Alzheimer's disease (AD) such as tau aggregation, neuroinflammation and decreased neurogenesis, whilst looking for a third action in beta‐secretase (BACE1), responsible of β‐amyloid production. We obtained well‐balanced MTDLs with in vitro activity in three different relevant targets and efficacy in two cellular models of AD. Furthermore, computational studies confirmed how these compounds accommodate adequately into the long and rather narrow BACE1 catalytic site. Finally, we employed in situ click chemistry using BACE1 as protein template as a versatile synthetic tool that allowed us to obtain further MTDLs.

## Graphical abstract

Using different small molecules targeting protein kinases and BACE1 templated synthesis, we have obtained well‐balanced multitarget compounds. They have three different biological activities and may be a promising therapeutic approach for the treatment of complex diseases such as AD.

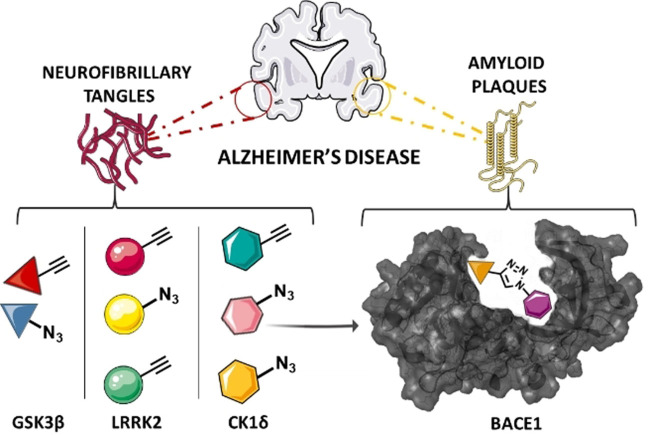

## Introduction

The lack of effective treatments for severe diseases characterized by a multifactorial etiology or therapeutic resistances, such as neurodegenerative disorders, cancer or bacterial infections, has led to the emergence of a new drug design paradigm: the multitarget approach strategy.[^1^, ^2^] Multitarget directed ligands (MTDLs) are characterized for showing activity in more than one molecular target taking advantage of multiple additive or synergic pharmacodynamic activities in a single molecule. This novel approach, that carefully selects polypharmacological drugs, contrasts with the previous classical design where drugs were designed to modulate a specific target with high selectivity, considering the multiple modulations of several targets undesirable. The single‐target molecule strategy has failed in the treatment of several pathologies including infectious diseases, cancer and neurodegenerative disorders and seems no longer appropriate.^[3]^ In order to address these pathologies, polypharmacy has been used in the clinical settings for numerous years targeting several biological systems at the same time.^[4]^ For example, it has been used in the treatment of cardiovascular diseases, cancer or HIV.^[5]^ However, the combination of different drugs for pharmacological treatment entails several risks and raises many difficulties such as unpredicted drug‐drug interactions or multiplied side effects in addition to lower patient compliance. With the specific aims of tackling several pathomolecular pathways simultaneously while reducing risks associated to polypharmacy, MTDLs are arising as an ideal strategy for the treatment of complex diseases.

Neurodegenerative diseases, and specifically Alzheimer's disease (AD), would greatly benefit from a MTDL approach.^[6]^ These complex diseases are characterized by their unknown etiology, their intricate molecular pathology and their multifactorial nature. AD is highly prevalent, and despite the great efforts to find an effective drug, it still presents high clinical failure. Furthermore, the complexity of the molecular pathology suggests that traditional drugs will not be able to produce a therapeutic effect.^[7]^ Due to this evidence, the association of multitarget compounds and neurodegenerative diseases has been growing in recent years and therefore some multitarget compounds have reached clinical trials.^[8]^ The specific design of these compounds needs to be carefully studied, being the main optimal characteristics the following: a) having a similar potency in all the modulated targets in order to enable a proper dose to tackle every pathobiological pathway efficiently, b) targeting proteins that present synergistic or additive effects reached with moderate activity that provides the opportunity to encounter a better safety profile, and c) preserving drug‐like properties.^[9]^

Specifically, the main pathological processes in AD are neurodegeneration, protein aggregation and brain inflammation. The major pathological aggregate hallmarks are β‐amyloid plaques and neurofibrillary tangles. β‐Amyloid plaques are extracellular neurotoxic deposits of β‐amyloid peptide caused, among other factors, by an abnormal processing by the beta‐secretase (BACE1).^[10]^ Neurofibrillary tangles are intracellular deposits composed of hyperphosphorylated protein tau. Multiple kinases including GSK3β, LRRK2 and CK1δ, among others, are involved in this pathological aggregation.[^11^, ^12^] Currently, different BACE1 and protein kinase inhibitors have reached clinical trials but none has been approved by the regulatory agencies so far.[^13^, ^14^] Our goal is to design multitarget compounds able to interfere simultaneously with three different targets involved in AD: BACE1 and, at least, two different protein kinases. In that sense, the new compounds could interfere with both proteinopathies present in AD. Moreover, since kinase inhibitors are also involved in the reduction of neuroinflammation and beta‐amyloid is emerging as a risk factor for many other diseases associated with aging,[^15^, ^16^] the combination of all the biological activities in a single molecule could create powerful drugs not only for AD but also for other dementias associated to frailty and aging. Several bifunctional molecules have already been discovered for the potential treatment of AD, however molecules able to combine 3 biological functions are more scarce. The first reported trifunctional molecules exhibited acetylcholinesterase (AChE) inhibitory activity together with the reduction of AChE‐induced amyloid‐β aggregation and metal chelating properties.^[17]^ Other discoveries have found molecules that tackle relevant AD targets such as AChE and its induced β‐amyloid aggregation, butyrylcholinesterase, monoamine oxidase or metal chelation.[^18^, ^19^, ^20^]

**Figure 1:**
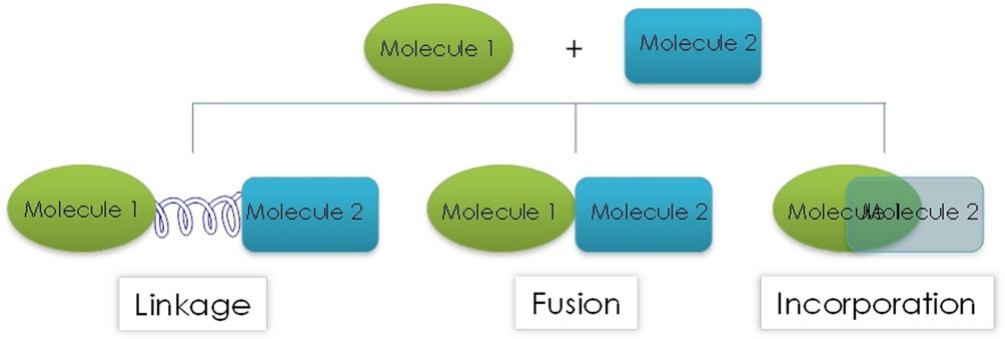
Different strategies used to generate multitarget compounds.

The rational design of MTDs is based in three main strategies: linkage, fusion and incorporation (Figure 1).

**Figure 2:**
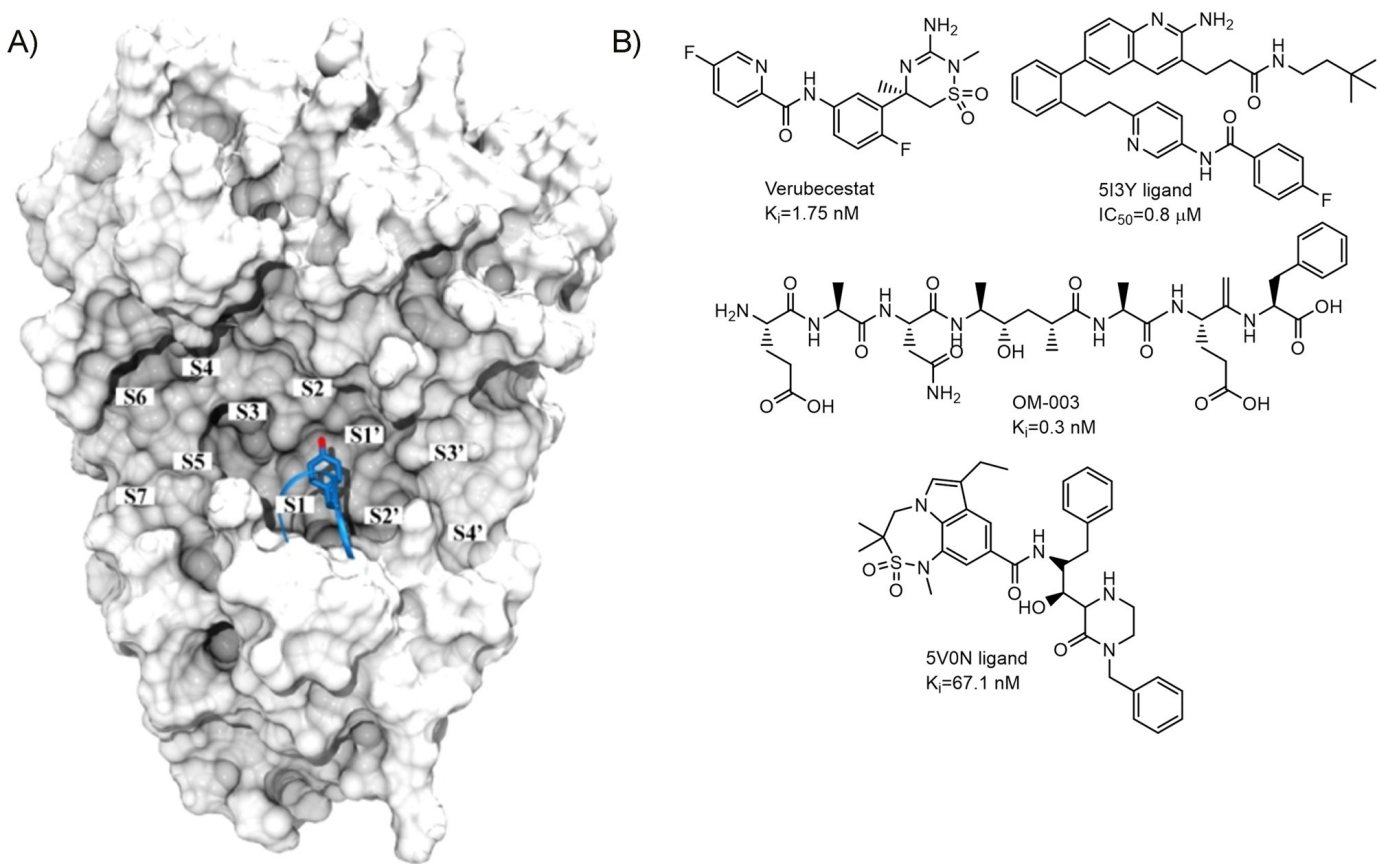
A) Representation of the catalytic site in BACE1. Flap and Tyr71 are shown in blue. B) Example of BACE1 inhibitors with heteroaromatic rings and amide functional groups.

Although the increase in molecular size and weight usually present in the design of multitarget compounds is one of the major disadvantages,^[21]^ the long and rather narrow size of the BACE1 catalytic site appears to be suitable for the linkage strategy in a MTDL design. The volume gained by linking two different protein kinase inhibitors could adjust into BACE1 catalytic site, which is able to accommodate eleven amino acid substrates (Figure 2 a).^[22]^ Inhibitors for this protease are normally large molecules able to maximize interactions in this cavity stabilizing the flexible loop on its N‐terminal domain known as “the flap” through its Tyr71 and locking BACE1 in an inactive conformation.^[23]^ The central binding pockets on the active site bind hydrophobic groups such as the ones found in peptidomimetic compounds based on substrate transition‐state analogues^[24]^ or other scaffolds like those showed in Figure 2 b, including verubecestat,^[25]^ OM‐003,^[26]^ 5I3Y ligand^[27]^ and 5V0N ligand.[^28^, ^29^] The BACE1 pharmacophore of amidine compounds, the most advanced BACE1 inhibitors in clinical trials, highlights the importance of heteroaromatic rings linked through an amide bond to optimally occupy S1 and S3 pockets.^[30]^ Therefore our working hypothesis consisted in the use of the linkage design to build BACE1 inhibitors starting from kinase inhibitor fragments and thus, obtaining MTDLs with both activities on the different AD hallmarks. Additionally, the linkage strategy also offers the possibility of connecting original scaffolds in a manner that can retain their primitive activity and interactions with the initial target.

In this work, we describe the design, synthesis, biological evaluation and suggest a binding mechanism of novel multitarget compounds with BACE1 and protein kinase inhibition activities. Furthermore, click chemistry and protein‐templated synthesis using BACE1 as scaffold has been used as versatile synthetic tools. Finally, different cellular assays have also been used to confirm the polypharmacology and applicability of our MTDLs.

## Results and Discussion

### Design and Evaluation of a Multitarget hit as Proof of Concept

In order to test our working hypothesis, we firstly synthesized two multitarget compounds, **3** and **8**, to evaluate whether linking two derivatives of a kinase inhibitor would be sufficient to gain BACE1 inhibitory activity by accommodating them into the catalytic site. Specifically, we selected the benzothiazole‐based LRRK2 inhibitors as starting fragments.^[31]^ To connect the two protein kinase fragments, different linkers were assayed: an aliphatic thioether chain and the heterocyclic 1,2,3‐triazole. Compounds **3** and **8** would serve as a proof of concept of activity preservation for LRRK2 inhibition and acquired BACE1 inhibition with molecules containing heteroaromatic rings linked through amide bonds.

**Scheme 1:**
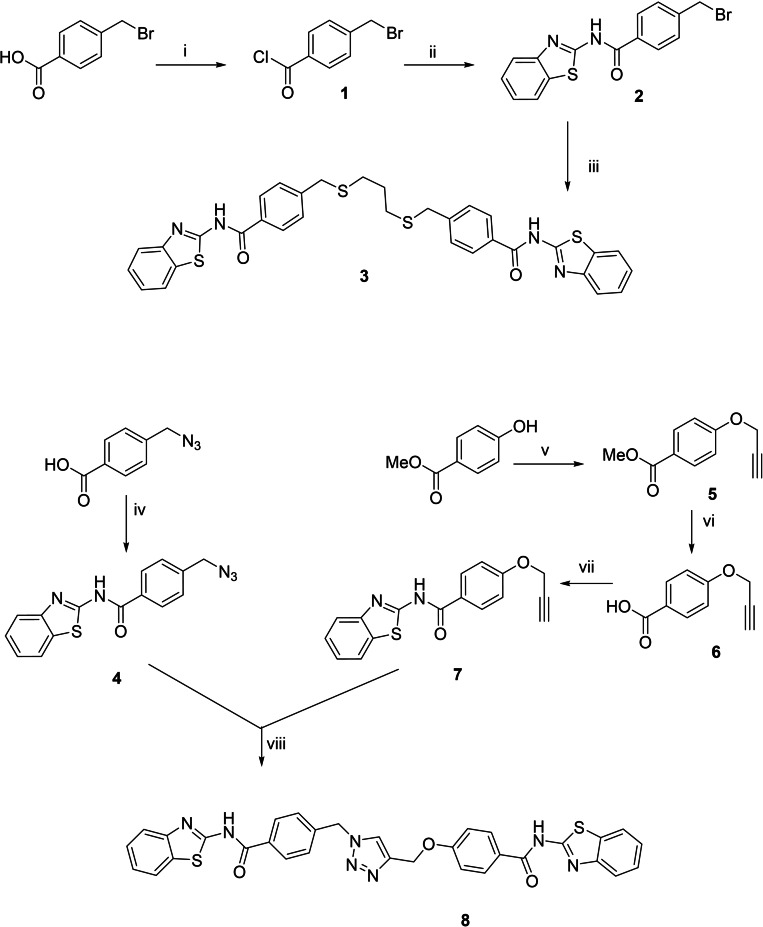
Synthesis of compounds **3** and **8**. i) SOCl_2_, 80 °C; ii) 2‐amino‐benzothiazole, 100 °C MW, THF; iii) K_2_CO_3_, propane‐1,3‐dithiol, THF, 80 °C; iv) EDCI, DMAP, DMF, r.t., 5 min. then 2‐amino‐benzothiazole, r.t.; v) 3‐bromopropyne, K_2_CO_3_, MeCN, 90 °C; vi) NaOH, MeOH:THF (1:1 v/v) r.t.; vii) EDCI, DMAP, DMF, r.t. 5 min. then 2‐amino‐benzothiazole, r.t.; viii) CuSO_4_⋅5 H_2_O, sodium ascorbate, DMF, r.t.

Synthesis of the thioether derivative **3** was performed coupling 2‐aminobenzothiazole **2** with propane‐1,3‐dithiol in basic conditions (Scheme 1).

Derivative **8** was obtained by click‐chemistry through a convergent synthesis. Azide fragment **4** was easily synthetized from 4‐(azidomethyl)‐benzoic acid and 2‐aminobenzothiazole coupling with EDCI and DMAP to form the amide bond. Alkyne **7** was obtained after three synthetic steps. Finally, the triazole **8** was synthesized using classic copper(I)‐catalyzed alkyne‐azide cycloaddition (CuAAC) (Scheme 1).

After in vitro biological evaluation using human recombinant enzymes, compounds **3** and **8** showed a well‐balanced inhibitory activity for the two targets. BACE1 was inhibited with IC_50_ values of 7.14 and 3.72 μM respectively, without losing LRRK2 inhibition (IC_50_ values of 1.88 and 1.83 μM, respectively). These compounds served as a first proof of concept for our working hypothesis. Additionally, kinetic experiments were done using human recombinant BACE1 and different concentrations of substrate and compound **8**. Lineweaver–Burk double reciprocal plot (Figure S1) showed a substrate‐competitive behavior in the inhibition of BACE1 by the multitarget compound **8**.

**Figure 3:**
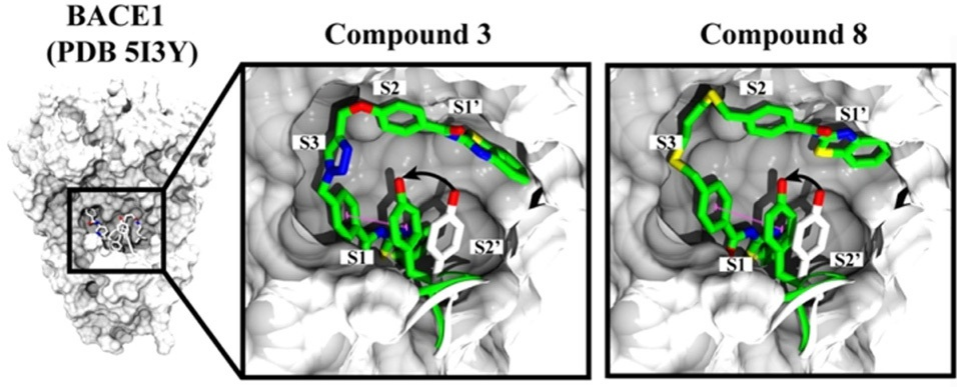
Suggested binding mode of compounds **3** and **8** within BACE1 catalytic site. Both the flap and residue Tyr71 from BACE1 crystal structure (PDB: 5I3Y) are represented in white. Compounds **3** and **8** as well as the flap and Tyr71 after docking are represented in green. π‐π interactions are showed as pink dotted lines.

Docking and binding free energy calculations showed how both inhibitors adopted similar poses within the BACE1 catalytic site (Figure 3). These large molecules established multiple hydrophobic contacts within pockets S1, S2, S3, S1′and S2′ of the protease and also stabilized the flap through a π‐π interaction with Tyr71. In fact, compared to the original BACE1 crystal structure, inhibitors **3** and **8** induced a closed conformation of the flap at the catalytic site, moving the Tryr71 2.7 Å and 3.6 Å, respectively.

Considering that both linkers presented similar inhibitory activity against the protease adopting a comparable binding mode, we selected the triazole **8** as our lead compound to obtain further multitarget molecules. Moreover, 1,2,3‐triazoles may be considered as amide bioisosteres^[32]^ and are readily obtainable through click chemistry. Therefore, we considered CuAAC as the best methodology to connect diverse heterocyclic fragments due to its versatility, accessibility and functional group tolerance.

**Figure 4:**
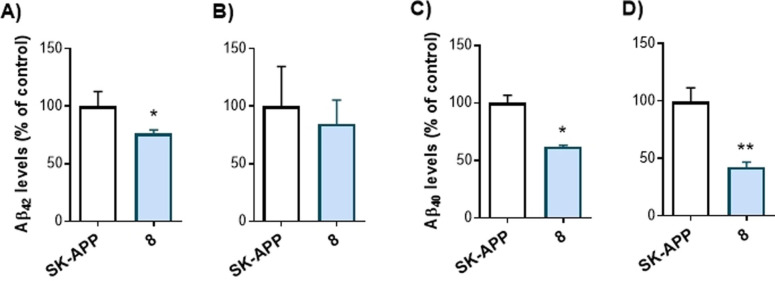
Reduced levels of Aβ_42_ and Aβ_40_ in SK‐APP cells after 48 h treatment with compound **8** at 10 μM. A) and C) extracellular fluid; B) and D) cellular lysates. Data are presented as mean ± SEM of at least three independent experiments performed in triplicate. *p<0.05; **p<0.01.

To explore the cellular activity of this MTDL regarding BACE1 inhibition, we tested the ability of compound **8** to decrease the production of toxic Aβ_40_ and Aβ_42_, in an amyloid protein precursor over‐expressing human neuroblastoma SH‐SY5Y cell line (SK‐APP cells). Levels of Aβ_40_ and Aβ_42,_ the two toxic species present in AD brains, were measured both in cellular lysates and in extracellular medium after 48 h of compound treatment. As shown in Figure 4, compound **8** exerts biological action at the cellular level probably due to BACE1 inhibition, reducing the Aβ_42_ levels in the extracellular fluid and Aβ_40_ levels in extracellular fluid as well as in cellular lysates.

With these excellent preliminary data showing that the new BACE1 in vitro activity found in the multitarget molecule is confirmed at the cellular level, we worked in exploring further compounds.

### Design, Synthesis and In Vitro Evaluation of New MTDLs: BACE1/Protein Kinases Inhibitors

Starting from molecules present in our in‐*house* chemical library,^[33]^ we selected the most interesting heterocyclic families targeting different protein kinases. Concretely, we chose GSK3β, LRRK2, and CK1δ inhibitors, that have shown inhibitory activity in the micromolar and submicromolar range with kinase selectivity, to design new multitarget compounds.

**Figure 5:**
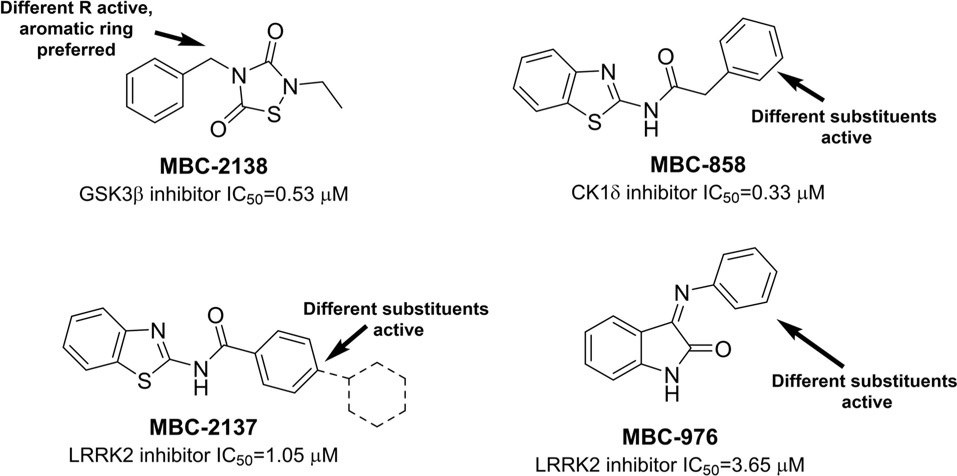
Different chemical modifications tolerated by the heterocyclic kinase inhibitors available at the MBC chemical library.

As the synthetic pathway chosen to create the MTDL involves the 1,3‐dipolar addition between azides and alkynes, we firstly examined the best positions in each heterocyclic family to introduce the alkyne and azide fragments without affecting the original biological activity of these kinase inhibitors. The most relevant leads for each family of kinase inhibitors are showed in Figure 5, indicating the potential chemical modifications tolerated based on previous SAR analysis.[^31^, ^34^, ^35^, ^36^] We selected one GSK3β inhibitor, one CK1 inhibitor, and two chemically diverse LRRK2 inhibitors as starting fragments for the MTDLs synthesis.

After the selection of the optimal sites for modification, different alkynes and azides were synthesized taking into account commercial availability of necessary reagents and synthetic efficiency.

Overall, five different fragments were obtained; two azides derived from LRRK2 and CK1δ inhibitors and three alkynes from LRRK2, GSK3β, and CK1δ inhibitors (derivatives **4**, **7**, **11**–**13**). In all cases, the synthesized fragments followed the original design incorporating the alkyne or azide at the optimal site for not interfering with the original protein kinase inhibitory activity. However, for the synthesis of the GSK3β inhibitor derivative, we encountered some synthetic difficulties since the needed reagents for the synthesis of the thiadiazolinone ring, isocyanates and isothiocyanates, are incompatible with a fair amount of chemical reactions. In this case, the strategy was subtly changed in order to obtain the fragment in an efficient and rapid manner. The aromatic ring needed for GSK3β activity was removed from the starting fragment, hypothesizing that the triazole formed after the click chemistry reaction would be positioned in a similar fashion in the final compound. In this way the linker was incorporated in the original GSK3β inhibitor structure (compound **12**).

**Scheme 2:**
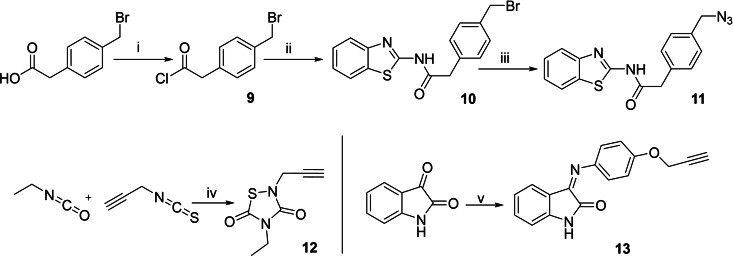
i) SOCl_2_, THF, 70 °C; ii) 2‐amino‐benzothiazole, 100 °C, MW; iii) NaN_3_, DMSO, r.t.; iv) SO_2_Cl_2_, 0 °C; v) 4‐(prop‐2‐yn‐1‐yloxy)aniline, EtOH, reflux.

The other fragments (alkynes and/or azides) were synthesized without further inconvenient (Scheme 2).

**Scheme 3:**
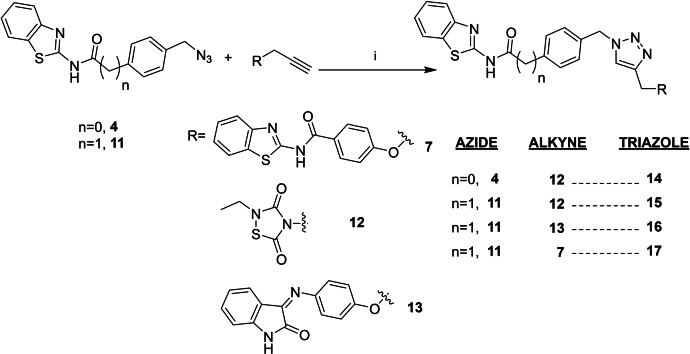
i) CuSO_4_⋅5 H_2_O, sodium ascorbate, DMF, r.t.

The multitarget compounds to be synthesized were designed in order to pair different kinase activities, building four different compounds that would yield MTDLs ideally showing inhibition of BACE1 and LRRK2‐GSK3β (**14**), CK1δ‐GSK3β (**15**), or CK1δ‐LRRK2 (**16, 17**), respectively (Scheme 3).

The inhibitory activity of the synthesized triazoles **14**–**17** was evaluated against human recombinant BACE1 and their two respective kinases, in order to establish whether these new chemical entities conserved the original inhibitory activities from their fragments and gained the new one as protease inhibitor (Table 1). Given that multitarget compounds come from selective kinase inhibitors serves as strong indicative of their selectivity against their kinase.[^27^, ^30^, ^31^, ^32^]

**Table 1:** Structure and activity of the 1,2,3‐triazoles in their respective enzymes.

No	Structure	LRRK2 IC_50_ (μM)	CK1δ IC_50_ (μM)	GSK3β IC_50_ (μM)	BACE1 IC_50_ (μM)
14	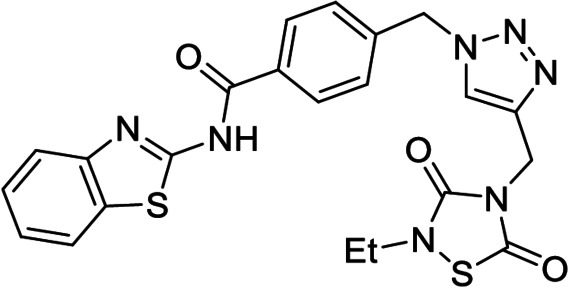	0.82±0.14	–	2.36±0.27	3.31±0.27
15	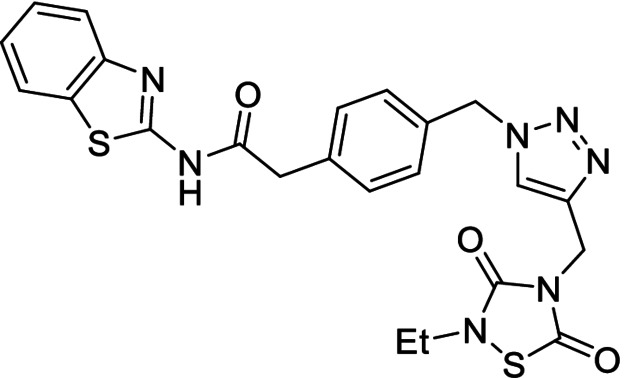	–	1.25±0.21	3.85±0.32	30 %^[a]^
16	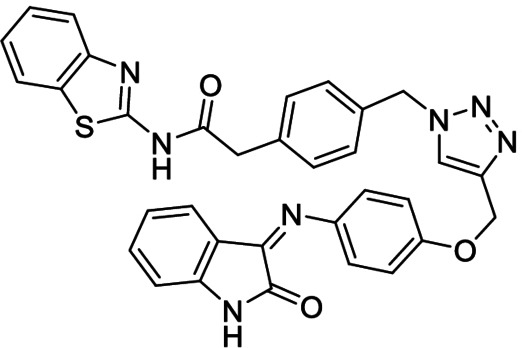	13.46±5.23	6.47±0.68	–	7.24±0.38
17	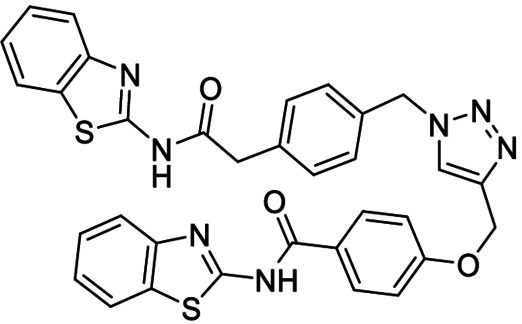	7.09±2.22	26 %^[a]^	–	2.51±0.43

[a] Enzyme inhibition at 10 μM. IC_50_ was not calculated due to poor solubility of the compound.

Results showed that most of the compounds inhibited BACE1 at the low micromolar range. Moreover, the original activity of the fragments was generally maintained, obtaining well‐balanced biological activities in three different targets and pointing to good candidates to modulate the AD pathobiological pathways efficiently at the same dose.

The BACE1 inhibitory activity of the original protein kinase inhibitors used to build the multitarget compounds was also tested, showing that the initial fragments alone did not have the capacity to bind to this enzyme (Table S1). These results suggest that the starting protein kinase inhibitors lack of BACE1 activity, thus BACE1 inhibition found in compounds **14**–**17** was gained by the molecular volume acquired after the linkage of the two protein kinase inhibitors, confirming our initial hypothesis.

**Figure 6:**
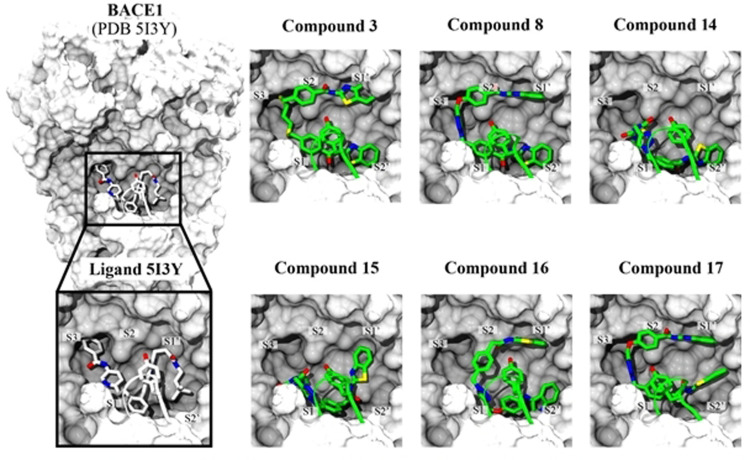
Binding mode of compounds **3** and **8** as well as synthesized triazoles within BACE1 catalytic site. Left panel show the crystallized structure of BACE1 in complex with 5I3Y ligand (PDB id 5I3Y).

The binding mode of these new MTDLs was explored with docking and free binding energy molecular dynamics studies, showing similar binding poses to compounds **3** and **8** with minor differences (Figure 6). For example, compounds **14** and **15** are smaller molecules and they preferentially occupy pockets S1, S2′ and partially S1′. Compound **15** and **16** showed a shifted pose leaving pocket S3 unoccupied.

### Cellular Activity of MTDLs

Cellular activity of the four new compounds (**14**–**17**) was evaluated in two different models in order to prove the beneficial effects of inhibiting BACE1 and the two different protein kinases.

Firstly, the human neuroblastoma cell line transfected with the precursor amyloid protein (SK‐APP cells) was used in order to confirm the ability of the MTDLs treatment to modulate Aβ_40_ and Aβ_42_ levels. A preliminary cellular toxicity study of these compounds at a fixed concentration of 10 μM and after 24 and 48 h of treatment, discarded compounds **16** and **17** from the experiment as they showed a significant decrease of cell viability at 48 hours (Figure S2).

**Figure 7:**
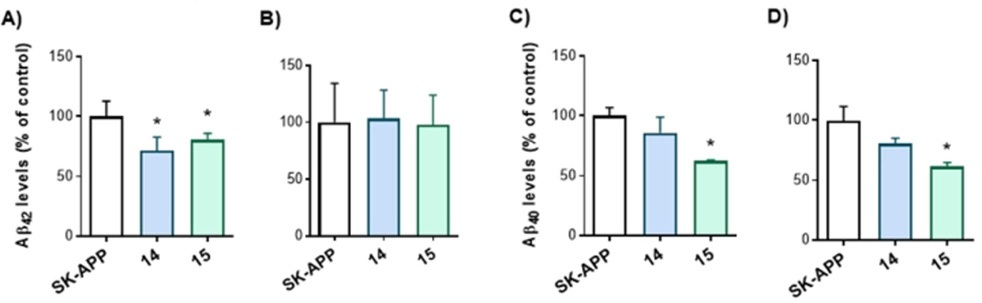
Reduction in the levels of Aβ_40_ and Aβ_42_ in SK‐APP cell line after 48 h treatment with compounds **14** and **15** at 10 μM. A) and C) extracellular fluid; B) and D) cellular lysates. Data are presented as mean ± SEM of at least three independent experiments performed in triplicate. *p<0.05.

The remaining compounds, **14** and **15** continued with the cellular characterization. Derivatives **14** and **15** exhibited a significant decrease in Aβ_42_ levels in extracellular fluids (Figure 7 A), while compound **15** was also able to decrease levels of Aβ_40_ both in cell lysates and extracellular media, showing their potential to interfere with toxic Aβ pathway (Figure 7 C,D).

These decreased Aβ levels could be the consequence of a reduction in Aβ secretion from APP processing. Although intracellular Aβ_42_ expression was unchanged, Aβ_40_ levels were reduced in cellular lysates mainly after compound **15** administration. This later result might suggest that these compounds were modulating Aβ production, and the subsequent release to medium. In view of our data, we suggest that compounds **14** and **15** provoked an important reduction of Aβ‐induced neurotoxicity, associated with diminished Aβ presence in the cell medium. Further dose dependent studies may be designed for more optimized lead compounds.

**Figure 8:**
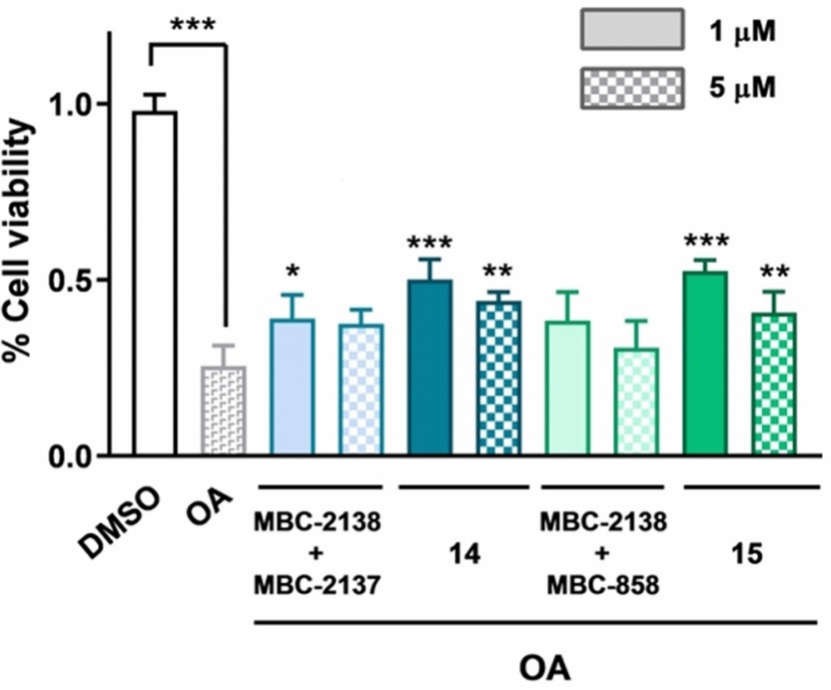
Effects of multitarget compounds and their precursors protein kinase inhibitors on the viability of SH‐SY5Y cells treated with OA.(In blue) Effects of GSK3β and LRRK2 inhibition. Cells were treated at the indicated doses with the combination of the individual kinase inhibitors (MBC‐2138 and MBC‐2137) or the multitarget triazole **14**. (In green) Effects of GSK3β and CK1δ. Cells were treated at the indicated doses with the combination of the individual kinase inhibitors (MBC‐2138 and MBC‐858) or the multitarget triazole **15**. Results expressed as % of control (DMSO) are the mean (± SEM) of at least three independent experiments performed in triplicate. (*p<0.05, **p<0.005, ***p<0.001 vs. treatment with OA only).

The protein kinase inhibition of the multitarget compounds, and specifically GSK3β, CK1δ, and LRRK2 inhibitory activities present in MTDLs **14** and **15** may interfere with tau phosphorylation, other of the hallmarks of AD. To evaluate the beneficial effects of our MTDLs in tau phosphorylation, the well‐known model of okadaic acid (OA) in human neuroblastome cell line SH‐SY5Y was used. OA is a serine/threonine phosphatase inhibitor able to induce tau hyperphosphorylation in cell culture with the subsequent cell death.^[33]^ Thus, neuroprotection of triazoles **14** and **15** was evaluated after 24 h of treatment in this model and compared to the biological effect produced by the simultaneous treatment with their individual protein kinase precursors, derivatives MBC‐2138 and MBC‐2137 for **14**; and MBC‐2138 and MBC‐858 for **15**. In Figure 8, the rescue from the toxic effect of OA can be observed after the treatment with new MTDLs **14** and **15**. The MTDLs were able to maintain or improve the neuroprotection showed by the combination of the two precursor fragments at cellular level, which highlights the advantage of MTDL versus polypharmacology approaches. Altogether, these data combined with the reduction of toxic β‐amyloid deposits in the SK‐APP cells demonstrates the potential of these compounds to treat complex neurodegenerative diseases such as AD and other dementias associated to aging.

### On Target BACE1 Directed Synthesis

In order to identify multitarget compounds with BACE1 and kinase inhibitory activity easily and effectively, we implemented the technology of in situ click chemistry.^[37]^ On template synthesis enables the formation of a product when reagents bound to the macromolecule acquire an optimal orientation for the chemical reaction to occur. In this manner, Huisgen cyclization can be performed in the absence of catalyst and at room temperature in a short time frame. We envisioned that by leveraging this methodology we could quickly identify BACE1 binders starting from kinase inhibitor fragments prior to synthesizing them in a bigger scale and thus saving resources.

**Figure 9:**
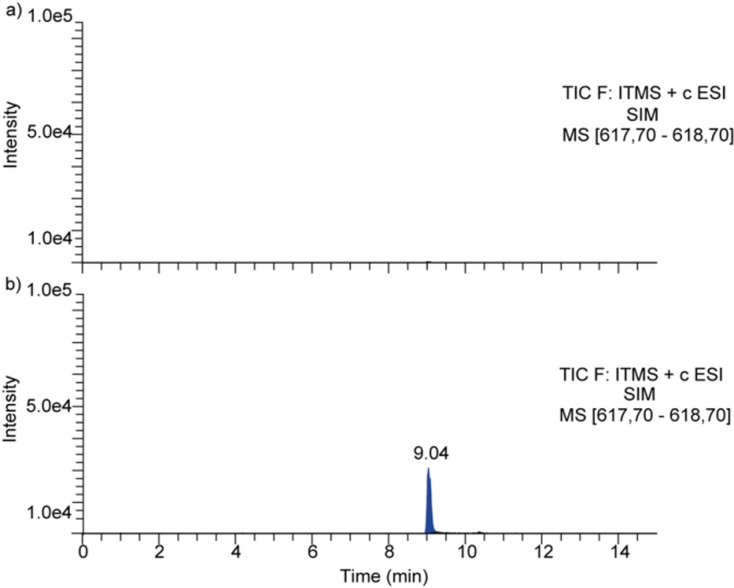
LC/MS‐SIM chromatograms of the incubation of azide **4** and alkyne **7**: A) without BACE1 and B) with 0.5 μM of BACE1.

Azide **4** and alkyne **7** were chosen to test the in situ click reaction with BACE1. Derivatives **4** and **7** were mixed in presence or absence of BACE1 in aqueous buffer and the mixtures were analyzed by LC/MS‐SIM. Concentration of the enzyme (0.25, 0.5, and 1 μM) and reagents (10, 50, 100, and 200 μM), temperature (20 and 37 °C), time (24 and 48 h) and buffer (phosphate‐buffered saline (PBS) pH 7.0 or sodium acetate pH 4.5) were screened in order to find the optimal conditions to test multiple reactions. Finally, selected experimental conditions were 24 h of incubation at room temperature using 50 mM sodium acetate buffer at pH 4.5, BACE1 at 0.5 μM and reagents at 100 μM. Only in the case of enzyme presence, a peak with the molecular weight of the expected triazole **8** was observed (Figure 9). Similarly, a control reaction in the presence of BSA was performed and no triazole MTDL was observed.

**Scheme 4:**
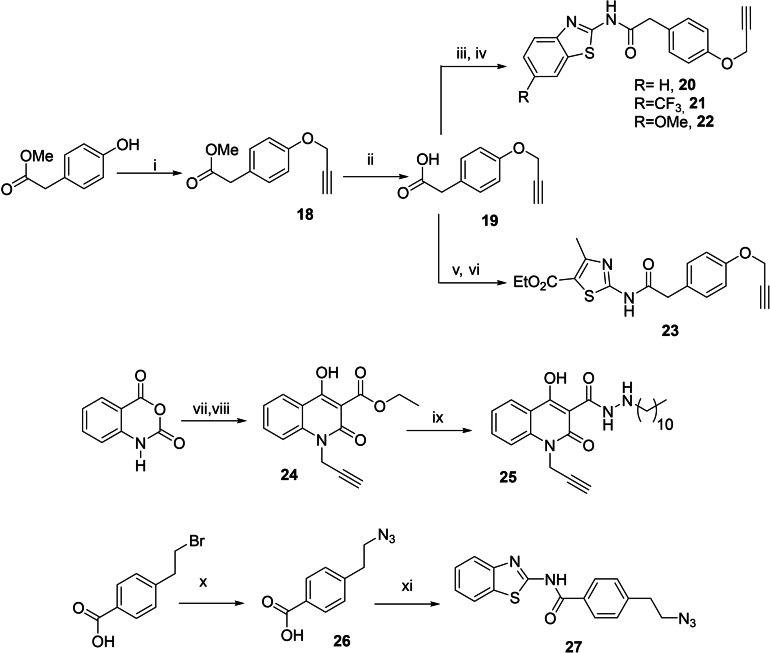
i) 3‐bromopropyne, K_2_CO_3_, THF, 80 °C; ii) NaOH, H_2_O:THF (1:4 v/v) r.t.; iii) SOCl_2_, CH_2_Cl_2_, 70 °C; then iv) 6‐*R*‐2‐amino‐benzothiazole, THF, 110 °C, MW; v) SOCl_2_, THF, 70 °C; then vi) ethyl 2‐amino‐4‐methylthiazole‐5‐carboxylate, THF, 120 °C, MW; vii) 3‐bromopropyne, NaH, DMF, 0 °C; viii) Diethylmalonate, NaH, DMF, 85 °C; ix) dodecanohydrazide, DMF, 160 °C; x) NaN_3_, DMF, 80 °C; xi) EDCI, DMAP, DMF,r.t., 5 min then 2‐amino‐benzothiazole, r.t.

To implement this methodology, other building blocks starting from different known protein kinase inhibitors were designed, inspired by kinase inhibitors in our chemical library. Thus, five new alkynes derived from inhibitors of CK1δ and GSK3β together with a new LRRK2 azide were synthesized following previous described experimental conditions (Scheme 4).

In total, three different azides (**4**, **11**, and **27**) and eight diverse alkynes (**7**, **12**, **13**, **20**–**23**, and **25**), with inhibitory activities in their respective protein kinases LRRK2, CK1δ and GSK3β, were combined in order to further select multitarget compounds (Table 2).

**Table 2:** In situ click chemistry building blocks with their inhibitory kinase properties.

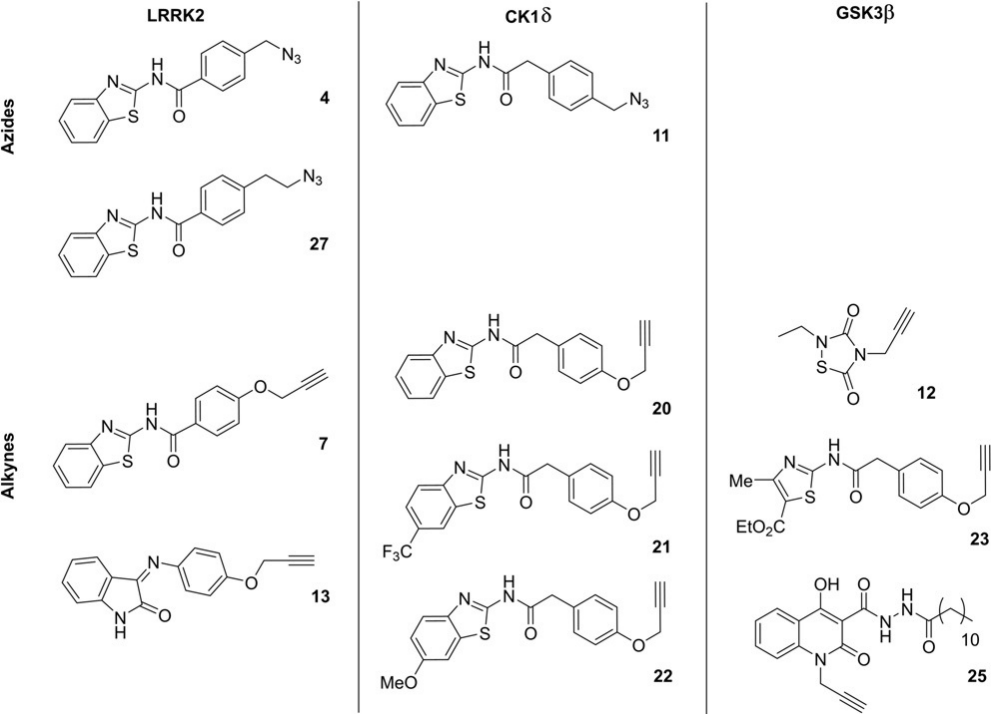

**Figure 10:**
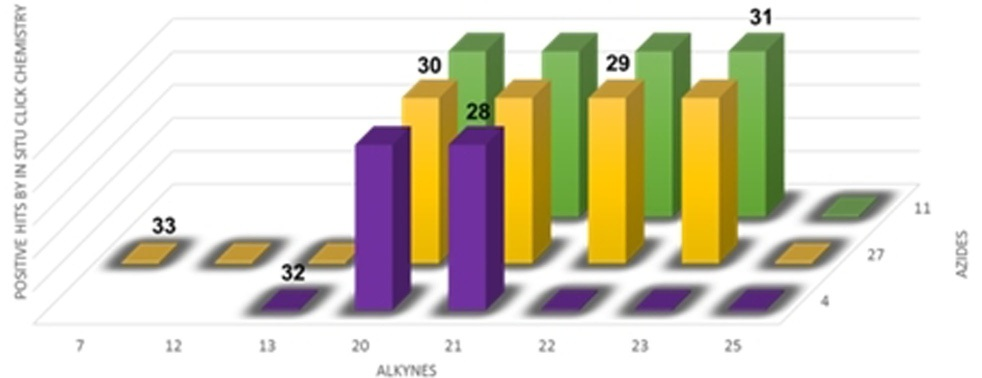
Hits obtained by in situ click chemistry reaction with BACE1 after incubation with the corresponding building blocks for 24 hours. Compounds synthetized to confirm the hypothesis are numbered above the bars: 28–31 positive hits and 32–33 negative hits. Data presented here is a result of two independent runs.

In situ click chemistry experiments were set incubating both fragments in the presence or absence of BACE1 and the reaction was analyzed by SIM LC‐MS. A total of nineteen independent reactions were set, from which we obtained eleven positive hits, observing that without the presence of BACE1 the subsequent triazole MTD was not being formed (Figure 10 and S3–S6).

**Scheme 5:**
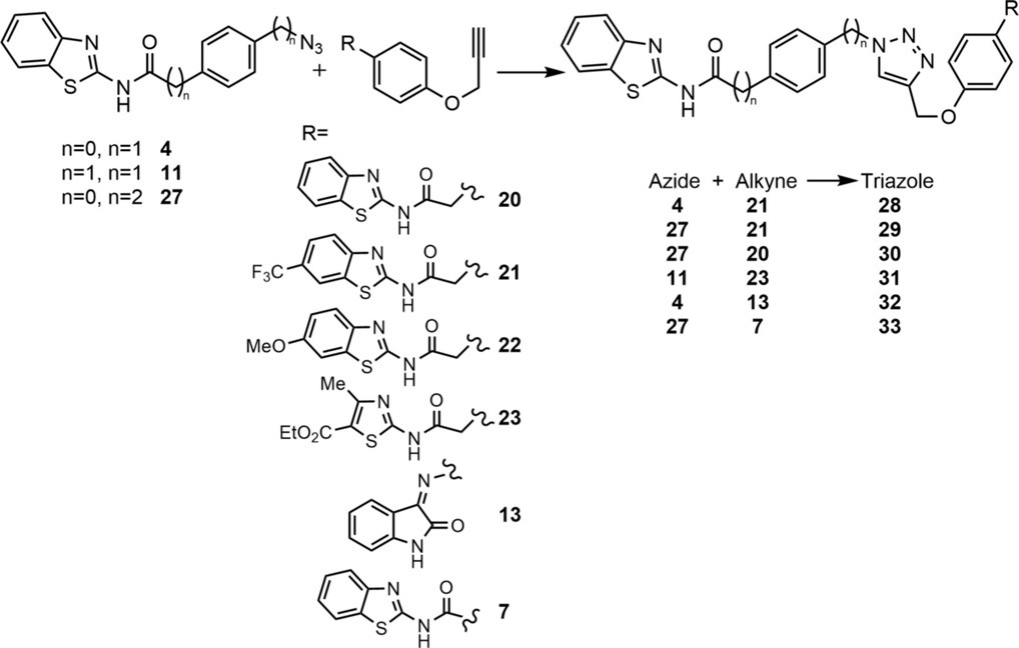
i) CuSO_4_⋅5 H_2_O, sodium ascorbate, DMF, r.t.

To fully confirm that hits obtained in this protein template synthesis correspond to BACE1 inhibitors, we synthesized four different MTDLs based in the availability of starting building blocks (compounds **28**–**31**). Furthermore, to control the reaction selectivity, two derivatives more (compounds **32** and **33**) were also prepared as negative controls (Scheme 5). In all cases, 1,2,3‐disubstituted triazoles were obtained using classical CuAAC.

Inhibitory activity of compounds **28**–**33** was tested on BACE1 and the corresponding protein kinases (Table 3). Results confirmed how hits selected by BACE1‐templated synthesis show inhibitory activity on this protease while negative controls lack this activity. In general, protein kinase inhibition profile was maintained in the multitarget compounds in a well‐balanced manner.

**Table 3:** Structure and activity of the new 1,2,3‐triazoles in their respective enzymes.

	Structure	LRRK2 IC_50_ (μM)	CK1δ IC_50_ (μM)	GSK3β IC_50_ (μM)	BACE1 IC_50_ (μM)
					
28	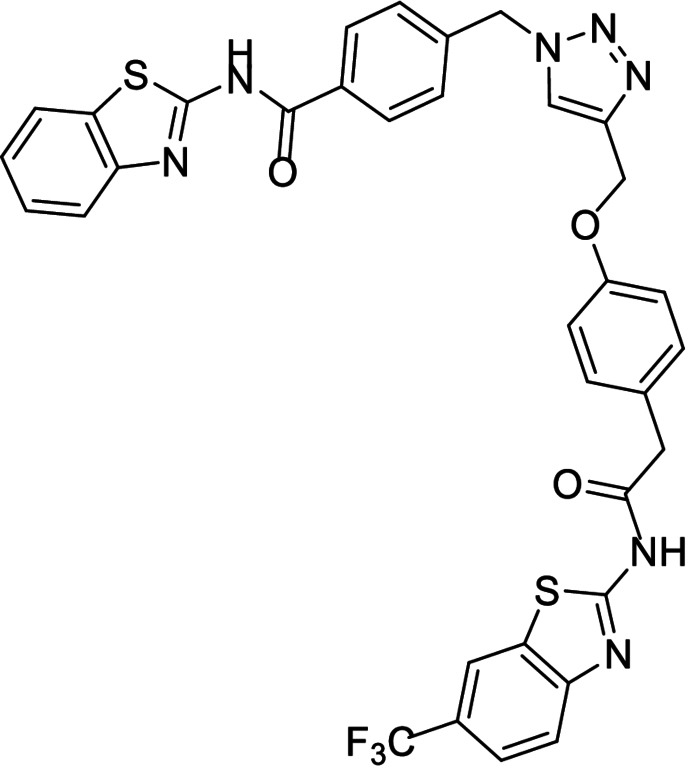	0.71±0.26	0.40±0.06	–	7.21±0.35
29	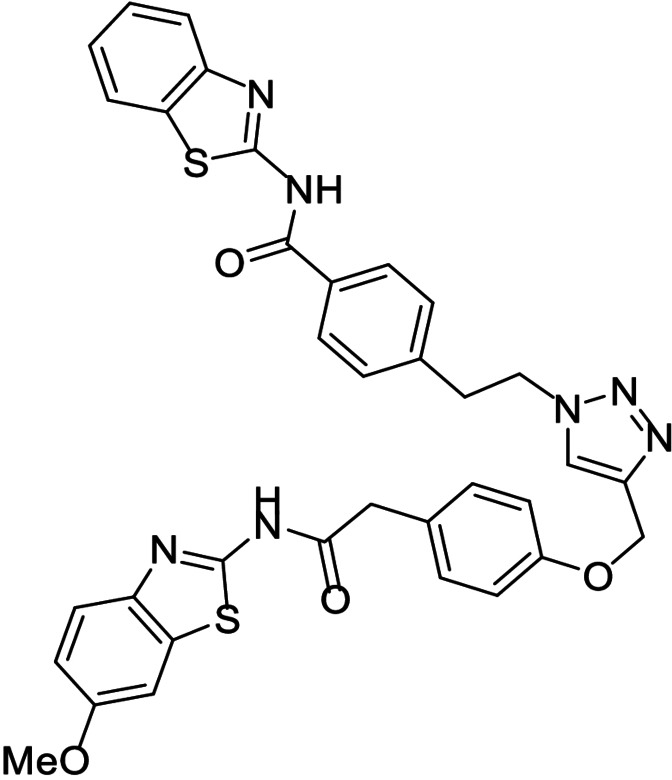	0.44±0.10	12.58±0.70	–	11.00±1.36
30	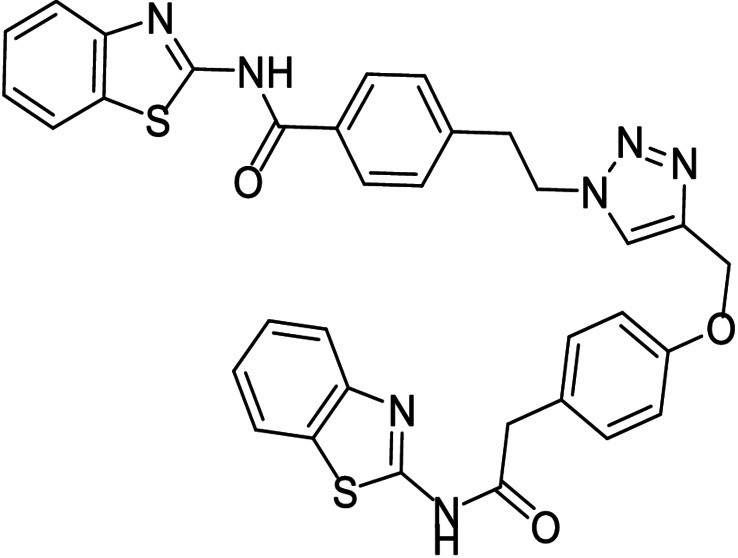	3.19±1.01	30 %^[a]^	–	8.57±0.45
31	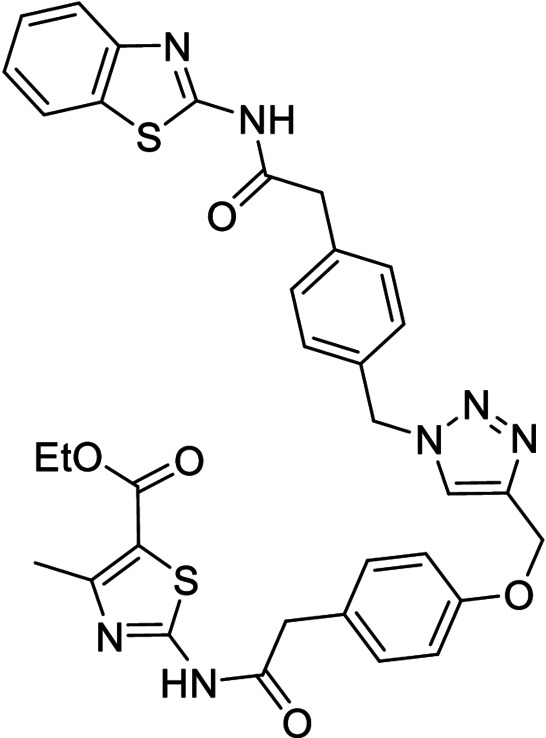	–	5.40±0.91	26 %^[a]^	6.76±0.61
32	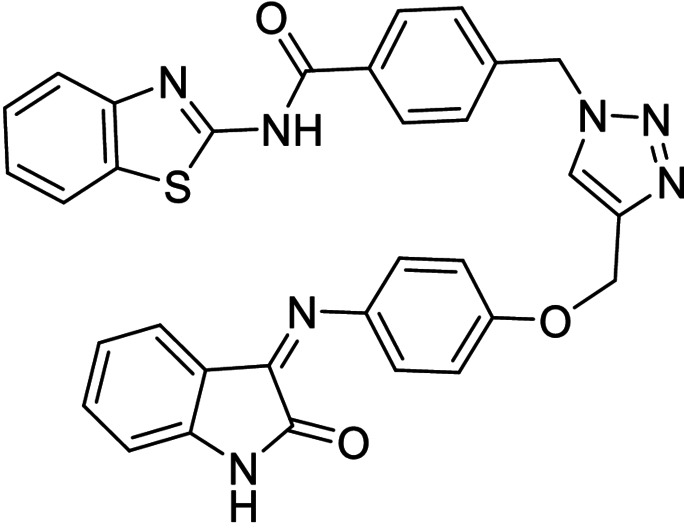	54 %^[a]^	–	–	<20 %
33	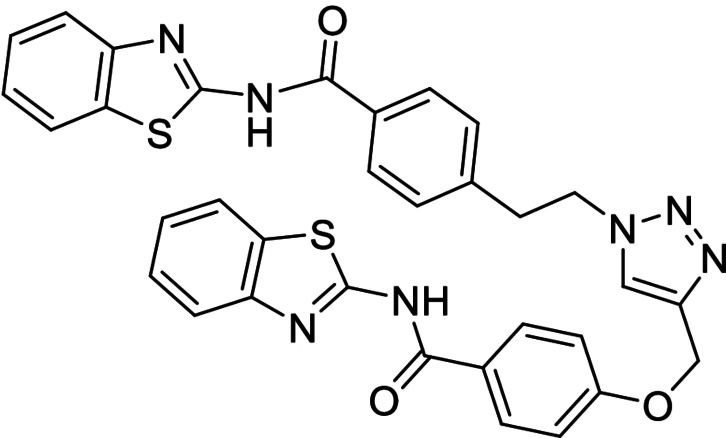	98 %	–	–	<20 %

[a] Enzyme inhibition at 10 μM. IC_50_ was not calculated due to poor solubility of the compound.

**Figure 11:**
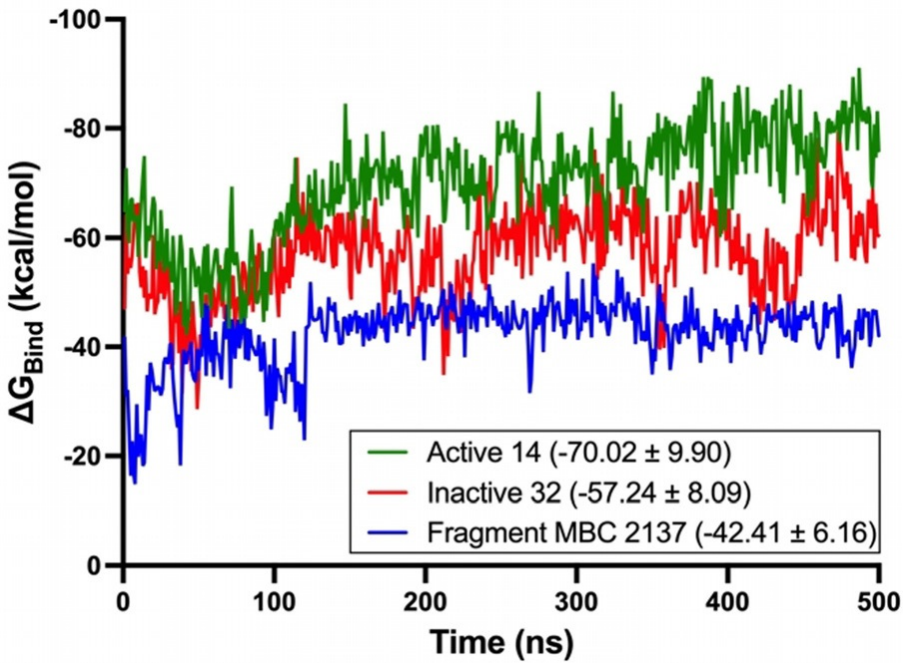
Δ*G*_Bind_ binding energy calculated using MMGBSA along the simulations. The Δ*G*_Bind_ average ± standard deviation (kcal mol^−1^) is displayed in the legend.

The in silico binding mode of the active triazoles on BACE1 was also studied, and it resembled those of compounds **3** and **8** (Figure S7). A detailed study of predicted binding energies (MMGSBA values) and interactions with key BACE1 residues have been done with MTDLs and the initial fragments (Table S2), showing lower energy values and different interactions for MTDLs BACE1 inhibitors (such as hydrophobic, hydrogen bonds and π‐π contacts with Try71 residue, or cation‐ π interactions with Arg128) that their corresponding starting fragments. Finally, a molecular dynamics study performed on MTDL compounds **14** and **32**, active and inactive on BACE1 respectively, together with their starting fragment MBC‐2137 show clearly how the active compound **14** present lower Δ*G*_Bind_ binding energy than the inactive **32** as well as the fragment MBC‐2137 (Figure 11). This analysis indicates that MTDL**14** presents more favorable interactions making its affinity for BACE1 higher, which is directly related to the inhibitory activity of BACE1 (Figures S8‐S13).

### Determination of Blood–Brain Barrier Penetration of MTDLs

**Figure 12:**
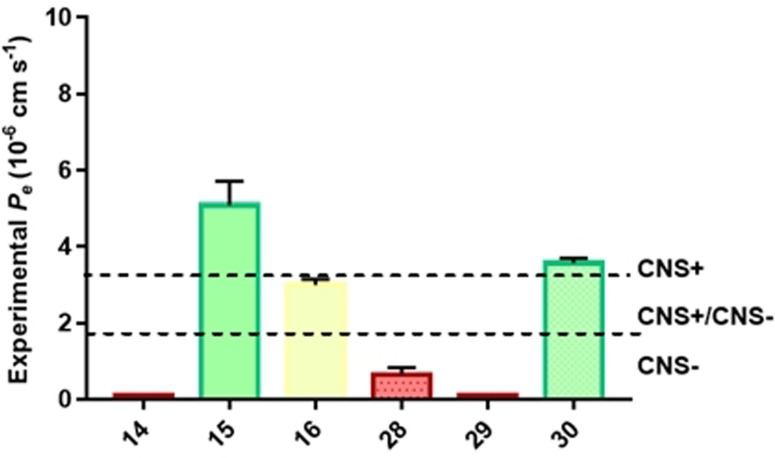
BBB prediction of multitarget compounds assayed by PAMPA methodology. CNS+ (green) for compounds with *P_e_* >3.20×10^−6^ cm s^−1^, CNS‐ (red) for compounds with *P_e_* <1.88×10^−6^ cm s^−1^ and CNS+/CNS‐ (yellow) for compounds with *P_e_* between both values. Data presented here is a result of two independent runs.

As stated in the introduction, one of the main disadvantages of the MTDLs is their increase in size and subsequent decline in pharmacokinetic properties. For the treatment of neurodegenerative diseases, a key pharmacokinetic feature is the permeation through the blood–brain barrier (BBB) that determines which compounds will be suitable candidates for the treatment of these diseases. Although there are several strategies to increase the BBB permeability of drug candidates using nanoparticles,^[38]^ in an attempt to characterize the pharmacokinetic profile of our multitarget compounds (**8**, **14**–**17**, **28**–**31**) we employed the parallel artificial membrane permeability assay (PAMPA) to predict the in vitro permeability (*P_e_)* by passive diffusion (Table S3 and Figure S14). While some compounds were insoluble in the conditions of the experiment (**8**, **17** and **31**), brain permeation prediction was calculated for MTDLs derivatives **14**–**16** and **28**–**30**. Compounds **15** and **30** showed apparent permeability values compatible with a positive brain penetration while compound **16** remains in the uncertainty region of prediction (CNS+/CNS‐). Compounds **14** and **29** were not detected in the acceptor well by HPLC‐MS and therefore experimental *P_e_* was assumed as 0 (Figure 12). These data confirm the difficulty of MTDLs to maintain good pharmacokinetic profiles but show two of the new MTDLs as potential brain penetrant compounds and therefore suitable agents to treat AD and other dementias. Further studies aiming to improve brain penetration of these compounds using PLGA nanoencapsulation are ongoing.[^39^, ^40^]

## Conclusion and Future Perspectives

In the recent decade, an unprecedented increase of polypharmacology and multitarget directed ligands (MTDLs) design strategies as the new chance to develop effective treatments for multifactorial severe diseases has been noted. We have here designed and synthetized new multitarget compounds that maintained their original kinase inhibitory activity adding a new BACE1 inhibitory effect. Therefore, we describe new MTDLs with great potential to treat complex diseases such as AD where Aβ pathology and aberrant phosphorylation events on tau protein are key events. The suggested binding mode of these compounds to BACE1 was simulated by molecular dynamic calculations showing how the compounds fit in the catalytic cavity closing it by direct interaction with the protein flap.

The new BACE1 inhibitory activity was further confirmed in a cellular model where these MTDLs were able to reduce Aβ_40_ and Aβ_42_ levels. Additional cellular assays were performed to test the potential of these multitarget compounds for protecting neural cells in the okadaic acid‐induced cell death model. MTDLs showed neuroprotective potential against the tau phosphorylation toxic effect with similar or improved potency than the simultaneous treatment with an equimolar mixture of their protein kinase inhibitor precursors. Besides we have demonstrated for the first time, how BACE1 can be used as template to select the fragments that may be exploited to obtain these and others interesting MTDLs. Additionally, we experimentally predicted that some of these MTDLs may penetrate into the brain by passive diffusion using PAMPA methodology.

Our strategy based on the interaction with the long catalytic site of BACE1 starting from individual protein kinase inhibitors to obtain new MTDLs has been confirmed. The cellular potency of the compounds has demonstrated how exploring this strategy could lead to promising compounds for the treatment of neurodegenerative diseases and specially Alzheimer's disease. In vivo evaluation of the brain penetrating multitarget compounds or the nanoencapsulated ones in a AD model will finally confirm the translational potential of these compounds.

## Conflict of interest

The authors declare no conflict of interest.

## Supporting information

As a service to our authors and readers, this journal provides supporting information supplied by the authors. Such materials are peer reviewed and may be re‐organized for online delivery, but are not copy‐edited or typeset. Technical support issues arising from supporting information (other than missing files) should be addressed to the authors.

Supporting Information
