# Supplementary material for: From Kinase Inhibitors to Multitarget Ligands as Powerful Drug Leads for Alzheimer's Disease using Protein‐Templated Synthesis
Source: Angew Chem Int Ed Engl. 2021 Jul 26;60(35):19344–54. doi: 10.1002/anie.202106295 (PMC8457121; doi:10.1002/anie.202106295)
Supplement: Supplementary file 1 — Supporting Information [file ANIE-60-19344-s001.pdf]

Angewandte Chemie
Eine Zeitschrift der Gesellschaft Deutscher Chemiker

Supporting Information

From Kinase Inhibitors to Multitarget Ligands as Powerful Drug Leads for Alzheimer's Disease using Protein-Templated Synthesis

Vanesa Nozal[+], Alfonso García-Rubia[+], Eva P. Cuevas, Concepción Pérez, Carlota Tosat-Bitrián, Fernando Bartolomé, Eva Carro, David Ramírez, Valle Palomo, and Ana Martínez**

anie_202106295_sm_miscellaneous_information.pdf

Index

Figure S1. Lineweaver-Burk double reciprocal plot of compound 8 in its inhibition of BACE1. 2

Table S1. Chemical structure and *in vitro* activity of the precursors of the fragments used in multitarget compounds 8, 14-15 in their respective protein kinases and BACE1. 3

Figure S2. Cell viability assat of MTDs cells.. 3

Figure S3. LC/MS-SIM of the optimization of BACE1 concentration. 4

Figure S4-6. LC/MS-SIM of azides 4, 11, 24 incubated with different alkynes in presence of BACE1.. 5

Figure S7. Binding mode of multitarget compounds (selected by *in situ* click chemistry) within BACE1 catalytic site. 10

Table S2. MMGSBA values and key interactions with BACE1 residues of MTDs compounds and their initial fragments……… …………………………..…….11

Figure S8. Number of interactions contacts along the simulations……………13

Figure S9. Interaction area between ligands and BACE1...............................14

Figure S10. BACE1-ligand interaction contacts during the 500ns simulations...15

Figure S11. Time dependence of the RMSD for ligand atoms and BACE1 backbone atoms during the 500 ns unrestrained MDs…………..……………16

Figure S12. BACE1 – ligands interactions throughout the simulation…,,,,,,,,…17

Figure S13. BACE1 – ligands interacting throughout the simulation.2………...18

Chemistry: Experimental procedures.. 11

Biological studies... 32

Computational studies.. 36

^{1}H and ^{13}C NMR Spectra... 39

Table S3. Permeability prediction in the PAMPA-BBB assay for the commercial drugs. 65

Figure S14. Linear correlation among experimental and reported permeability of commercial drug using the PAMPA-BBB assay 66

Supplementary bibliography.. 66

Figure S1. Lineweaver-Burk double reciprocal plot of compound 8 in its inhibition of BACE1.

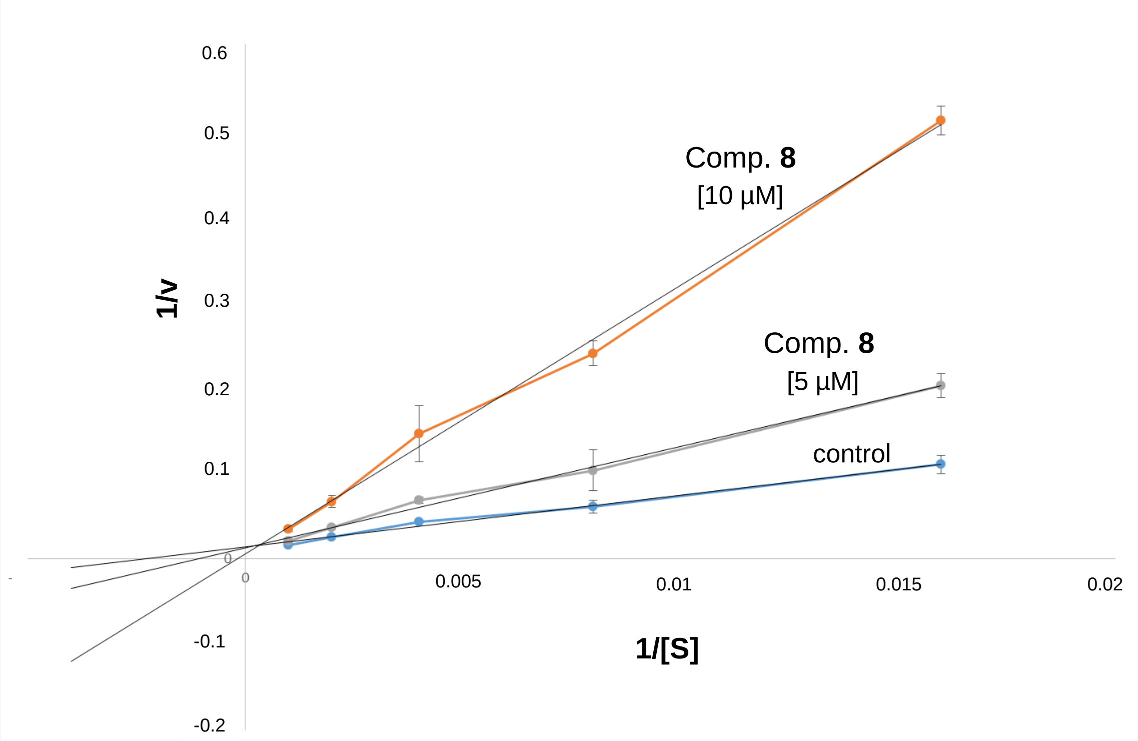

Table S1. Chemical structure and *in vitro* activity of the precursors of the fragments used in multitarget compounds **8, 14-15** in their respective protein kinases and BACE1**.**

**Code**	**Structure**	**LRRK2** IC_{50} (μM)	**CK1δ** IC_{50} (μM)	**GSK3β** IC_{50} (μM)	**BACE1** IC_{50} (μM)
**MBC-2138**	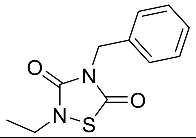	-	-	0.53±0.03	>20
**MBC-976**	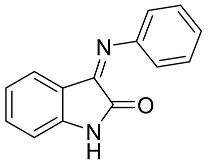	2.98±0.36			>20
**MBC-2137**	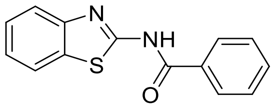	1.05±0.19	-	-	>20
**MBC-858**	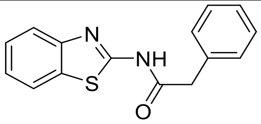	-	0.33±0.03	-	>20

Figure S2. Effect of the MTDs **8, 14-17** on the viability of cells: SK-APP cells at 10 μM A) after 24 h treatment, B) after 48 h treatment. C) SH-5YSY.after 24 h treatment.

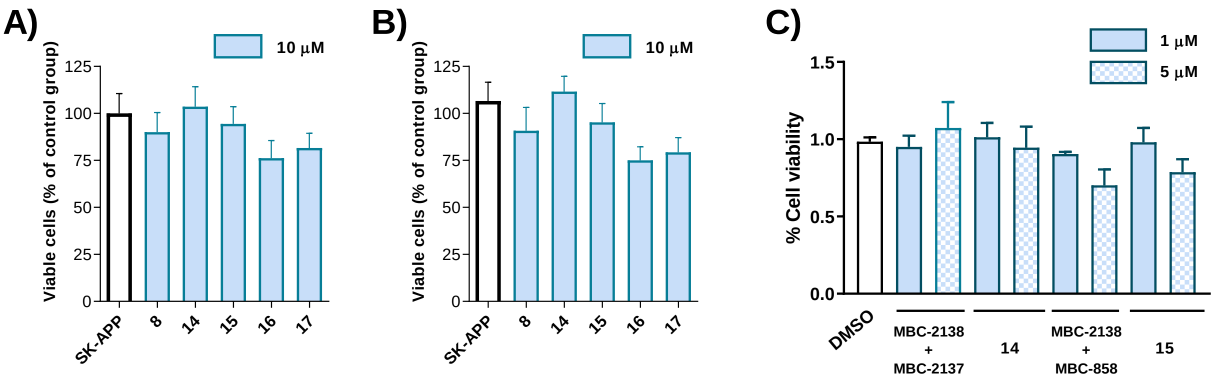

Figure S3. Optimization of BACE1 concentration. LC/MS-SIM for mixture azide **4** and alkyne **7** to provide triazole **8** when BACE1 is present: a) 0 µM, b) 0.25 µM, c) 0.5 µM d) 1 µM.

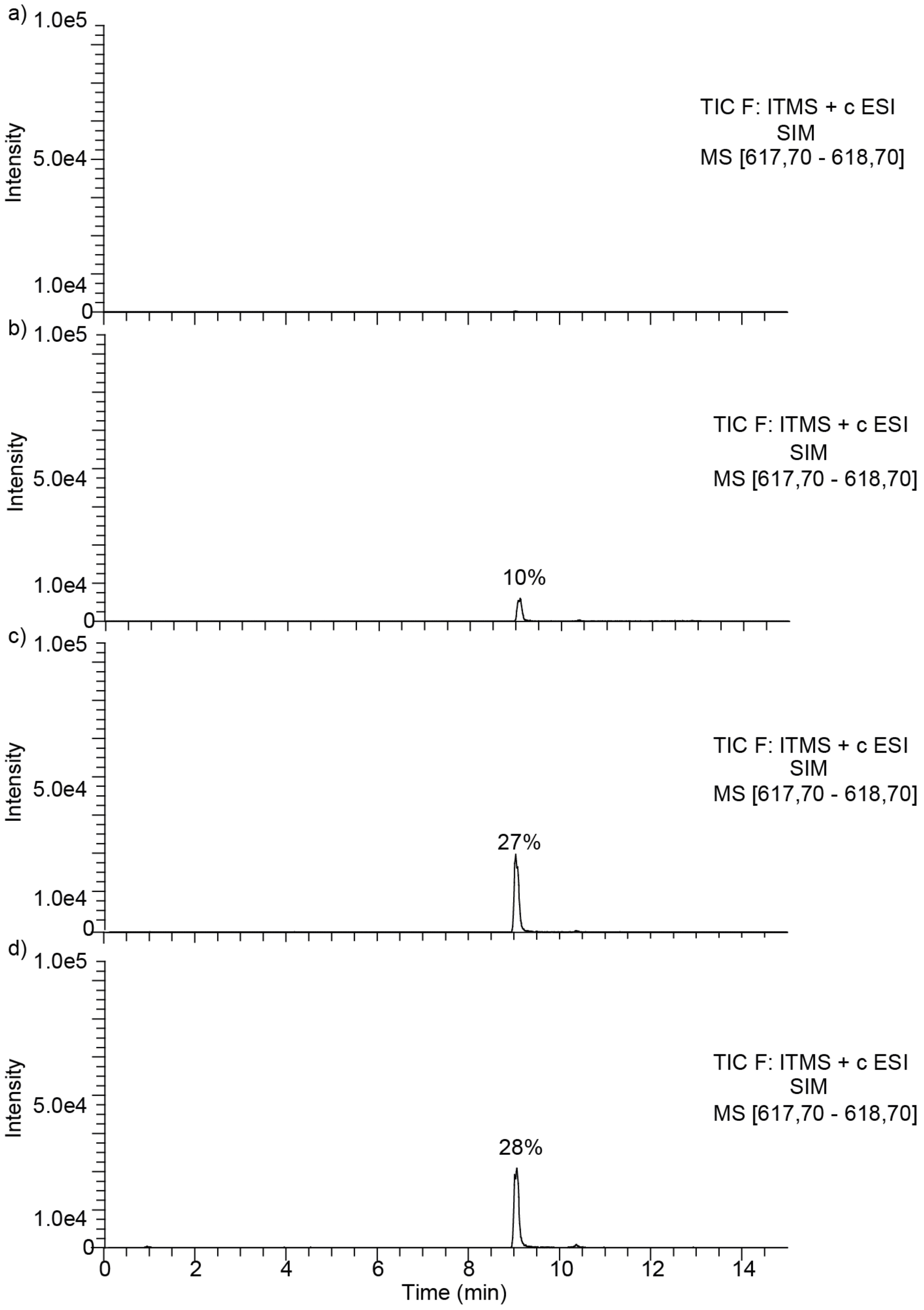

Figure S4. LC/MS-SIM of azide **4** incubated with different alkynes in presence of BACE1: a) alkyne **13**, b) alkyne **20**, c) alkyne **21**, d) alkyne **22**, e) alkyne **23**, f) alkyne **25.**

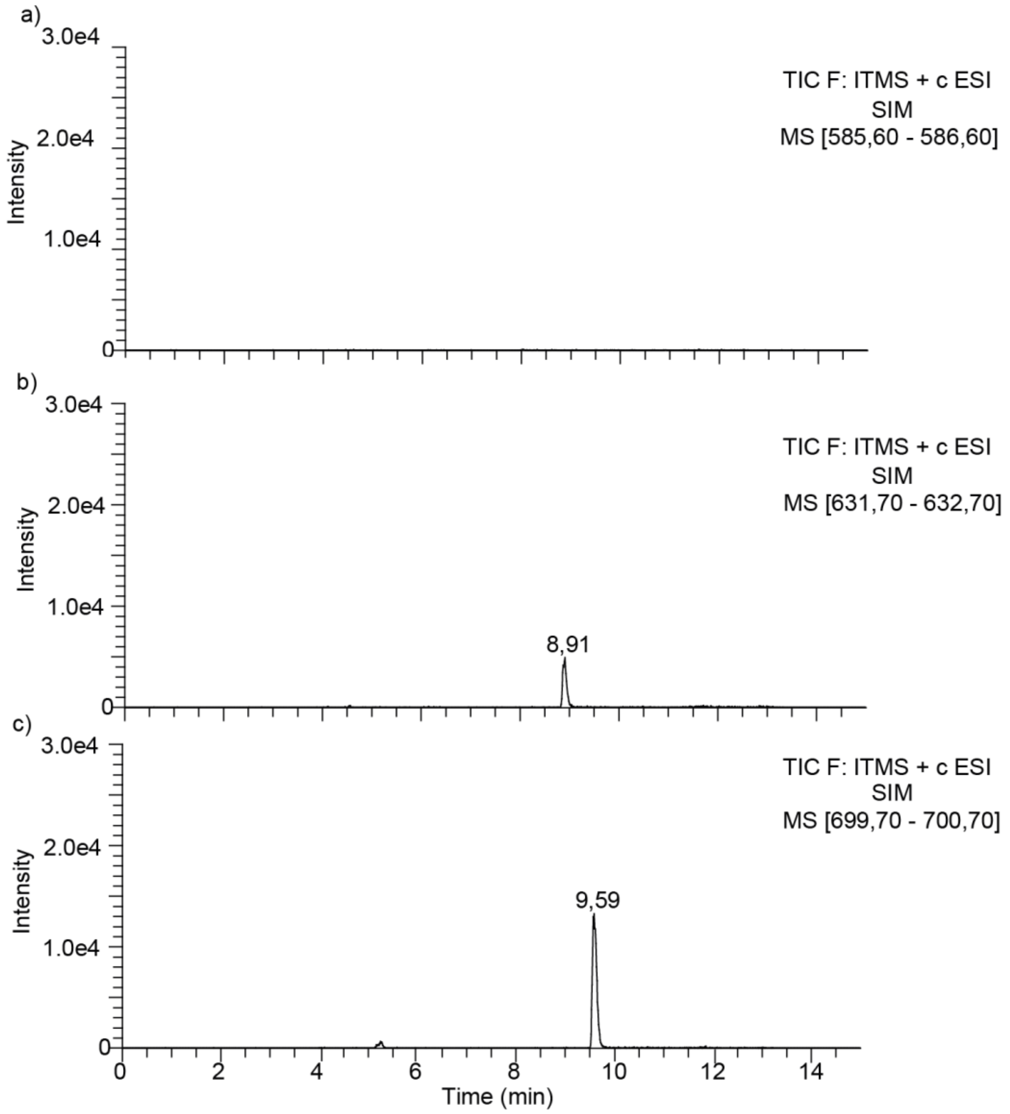

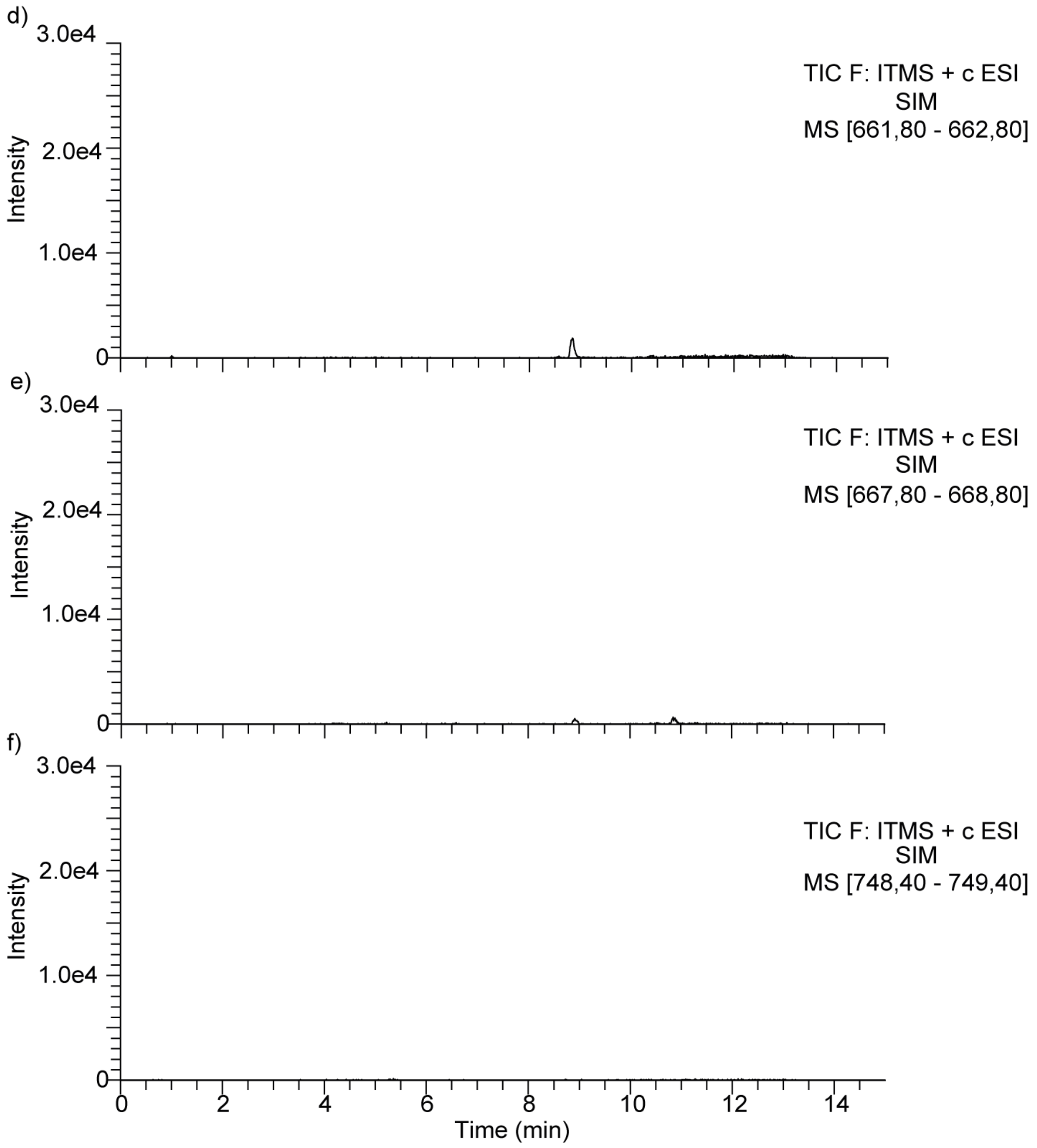
d)
3.0e4
2.0e4
1.0e4
0
Intensity
TIC F: ITMS + c ESI
SIM
MS [661,80 - 662,80]
e)
3.0e4
2.0e4
1.0e4
0
Intensity
TIC F: ITMS + c ESI
SIM
MS [667,80 - 668,80]
f)
3.0e4
2.0e4
1.0e4
0
Intensity
TIC F: ITMS + c ESI
SIM
MS [748,40 - 749,40]
0
2
4
6
8
10
12
14
Time (min)

Figure S5. LC/MS-SIM of azide **11** incubated with different alkynes in presence of BACE1: a) alkyne **20**, b) alkyne **21**, c) alkyne **22**, d) alkyne **23**, e) alkyne **25.**

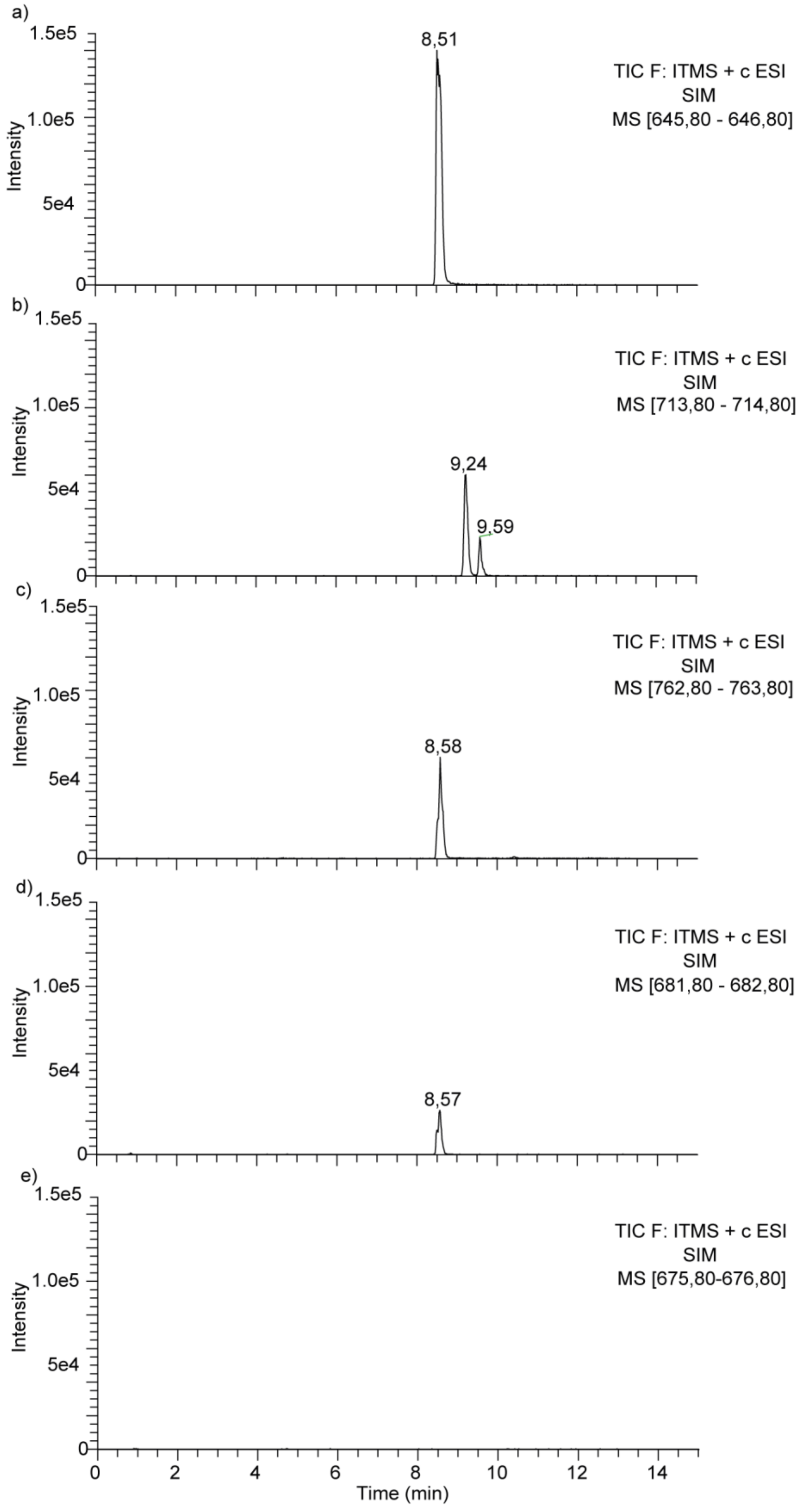

Figure S6. LC/MS-SIM of azide **27** incubated with different alkynes in presence of BACE1: a) alkyne **7,** b) alkyne **12**, c) alkyne **13**, d) alkyne **20**, e) alkyne **21**, f) alkyne **22**, g) alkyne **23**, h) alkyne **25.**

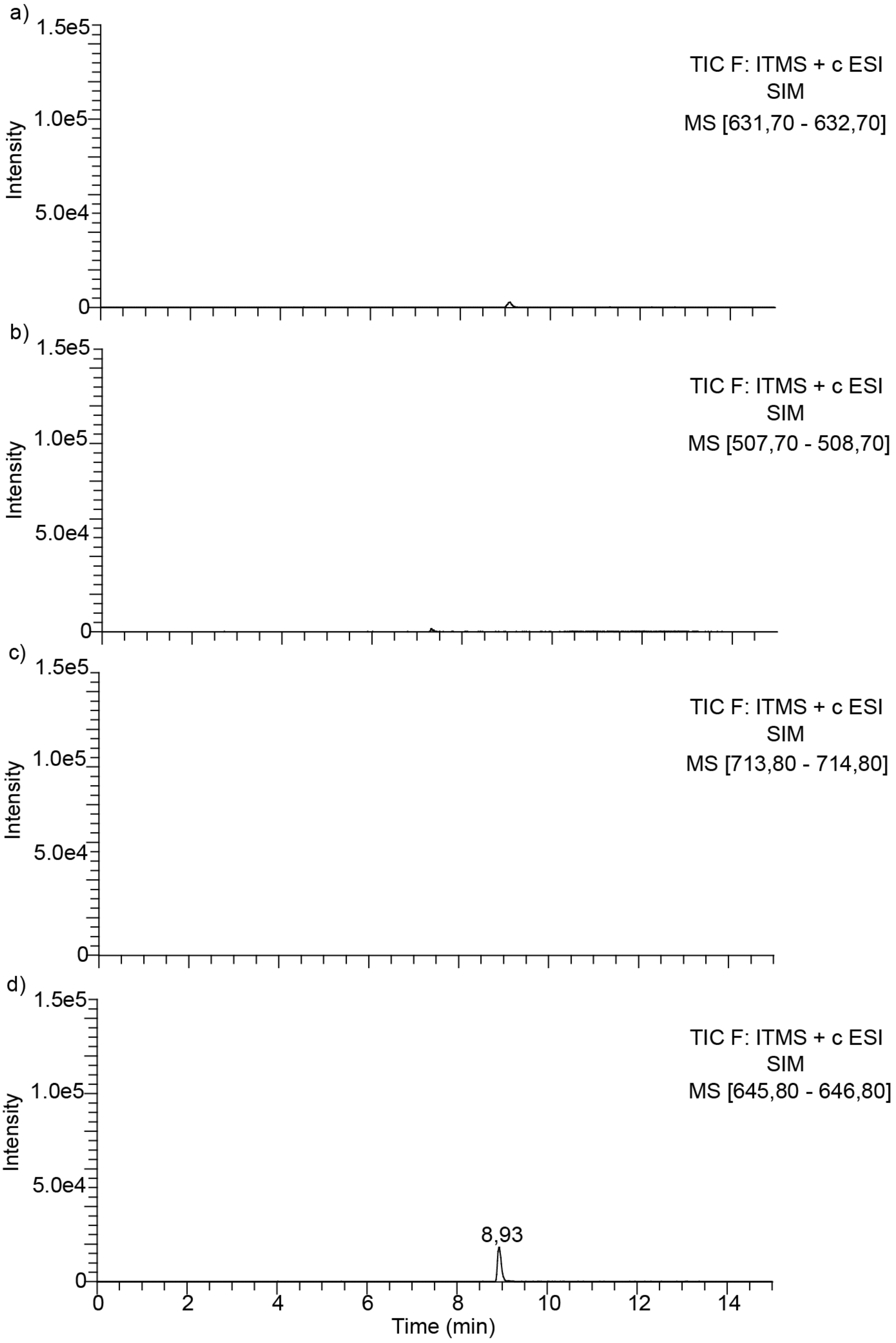

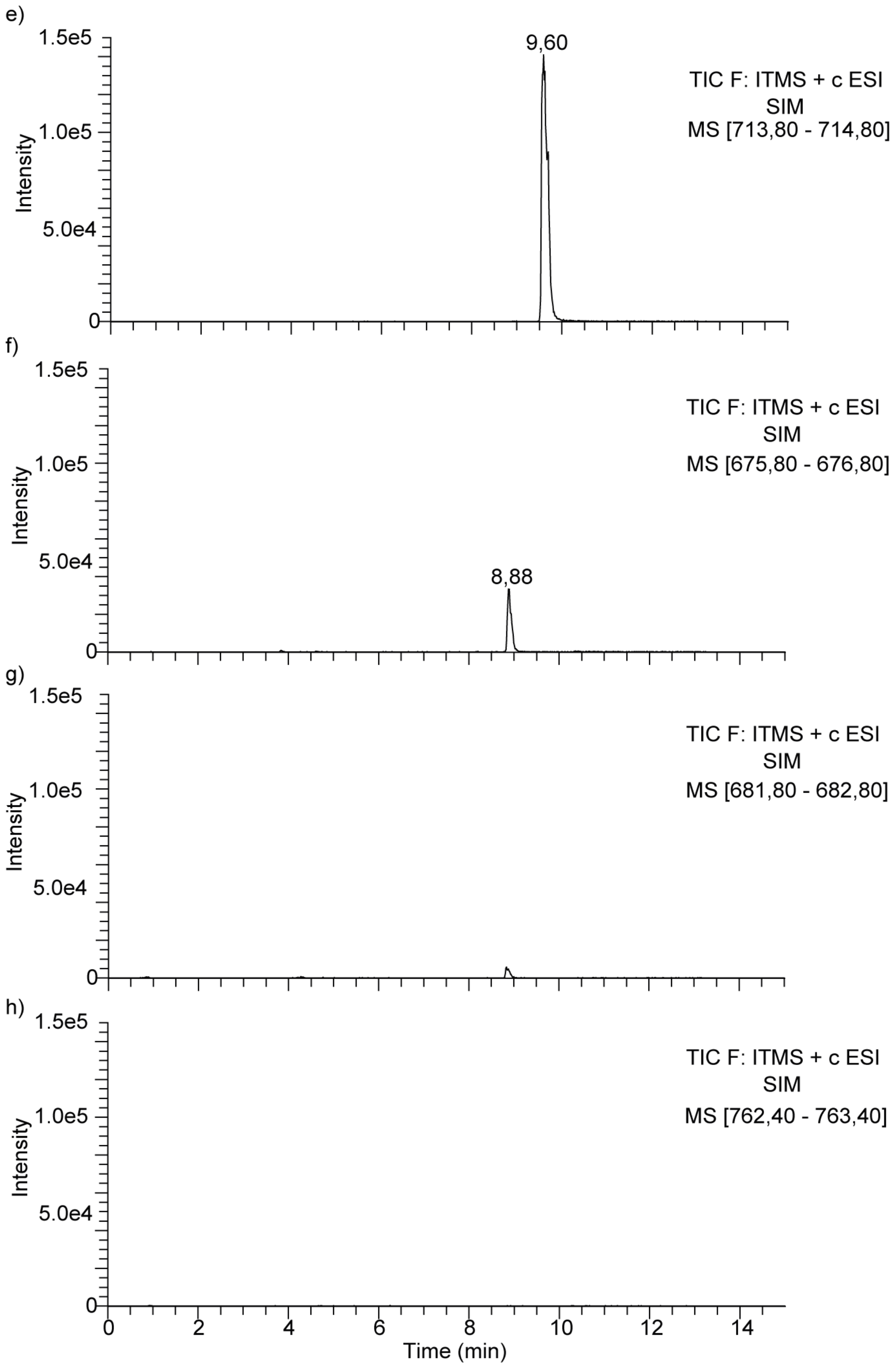

e)
9,60
TIC F: ITMS + c ESI
SIM
MS [713,80 - 714,80]
f)
8,88
TIC F: ITMS + c ESI
SIM
MS [675,80 - 676,80]
g)
TIC F: ITMS + c ESI
SIM
MS [681,80 - 682,80]
h)
TIC F: ITMS + c ESI
SIM
MS [762,40 - 763,40]
Intensity
1.5e5
1.0e5
5.0e4
0
0
2
4
6
8
10
12
14
Time (min)

Figure S7. Binding mode of multitarget compounds (selected by *in situ* click chemistry) within BACE1 catalytic site. Left panel show the crystallized structure of BACE in complex with aminoquinoline (PDB 5I3Y).

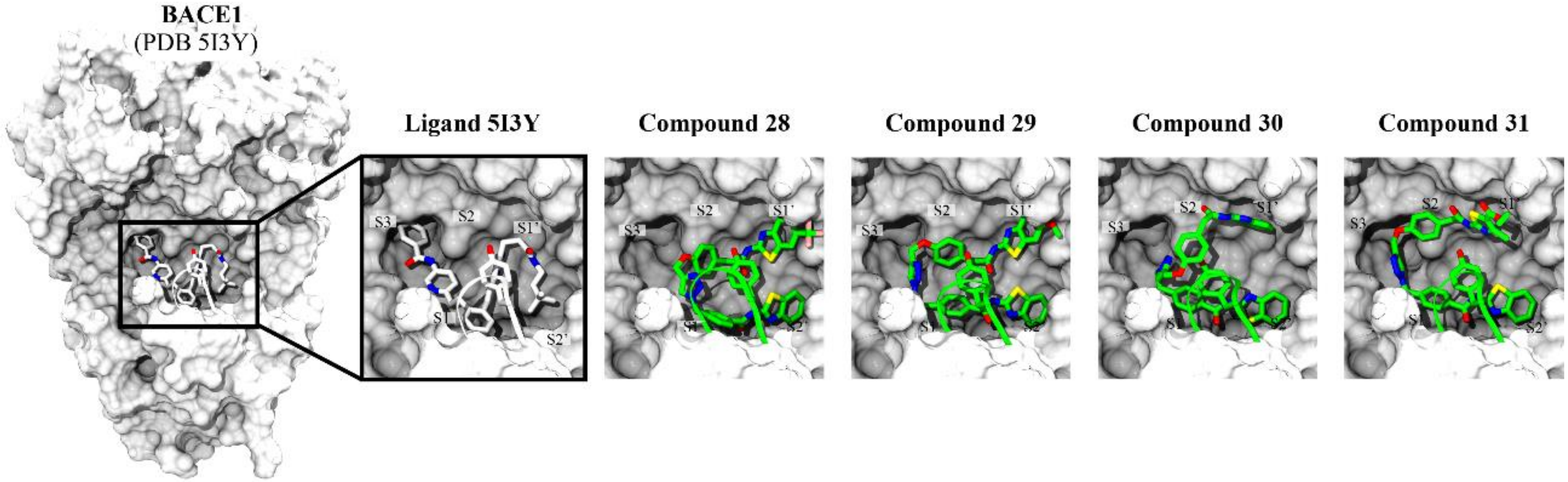

Table S2. MMGSBA values and key interactions with BACE1 residues of MTDs compounds and their initial fragments

Compound	3	8	14	15	16	17	28	29	30	31	MBC-858	MBC-976	MBC-2137	MBC-2138
MMGBSA (Kcal.mol^{-1})	-98.26	-70.48	-74.00	-51.28	-63.36	-53.91	-77.87	-61.95	-61.20	-58.76	-42.38	-43.93	-43.27	-46.76

	Compound	3	8	14	15	16	17	28	29	30	31	MBC-858	MBC-976	MBC-2137	MBC-2138
BACE 1 residues	LEU30	H		H	H		H			H		H			
	ASP32											HB			
	ASN37	H				HB			H					H	
	ALA39													H	
	VAL69	H	H		H	H				H			H	H	
	TYR71	Pi; H	Pi; H	HB; Pi; H	HB; Pi; H	HB; Pi; H	HB; Pi; H	Pi; H	Pi; H	Pi; H	HB; H	Pi; H	H		H
	THR72									H	H				
	GLN73			HB		H		HB		H					
	TRP76		HB; H	HB		HB		HB	HB	HB; H	HB; H		HB; Pi	Pi; H	HB
	LYS107														
	PHE108	H	H	Pi; H	Pi; H	H	Pi; H	Pi; H	Pi; H	H	Pi; H	Pi; H	H	H	Pi; H
	ILE110	H			H										
	TRP115	H		H	H				H	H		H			
	ILE118	H	H	H	H		H		H	H	H	H		H	H
	ILE126	H	H	H		H		H	H	H	H				
	ARG128	Cat	Cat	Cat		Cat	Cat	Cat	Cat	Cat	Cat		Cat	HB	
	TYR198	H			H	H	H	H	H	H		H		H	
	ILE226	H			H	H	H	H	H	H		H			
	ASP228							HB	HB						
	THR231	HB; H	HB; H				HB; H				H				
	THR232		HB				HB				HB				
	ASN233														
	ARG235	HB.													
	THR329		H			H	H			HB.	H				
	VAL332	H	H				H		H	H					

HB: Hydrogen Bonds; **H:** Hydrophobic; **Pi:** Pi-Pi interactions; **Cat**: Pi-Cation interactions

Figure S8. Number of interactions contacts (Hydrogen bonds, Pi-Pi stacking and Pi-cation) along the simulations. The number of interactions of compounds **14** and **32** are much higher than those of the MBC-2137 fragment, because compounds **14** and **32** are larger and can establish more interactions with the active site residues of BACE1. This is reflected in the ΔG_{Bind} binding energy (Figure 11).

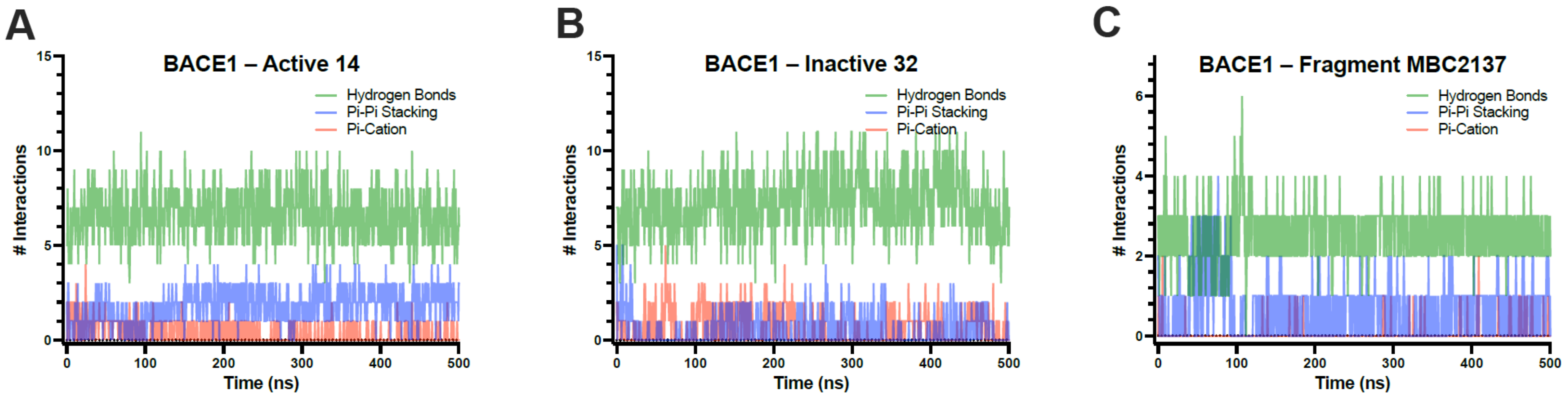

Figure S9. Interaction area between ligands and BACE1. Due the larger structure of **14** and **32**, the interaction area between the BACE1 binding site and these compounds is larger than the interaction area between the fragment MBC-2137 and BACE1. Having a larger interaction area allows **14** and **32** to establish a higher number of interactions (and more stable interactions) with the residues of BACE1 binding site. Due to the nature of the binding site of BACE1 (a very large site), it is important to have a large interaction area in order to anchor to the active site and thus have a longer residence time, which can be reflected in increased activity (potency).

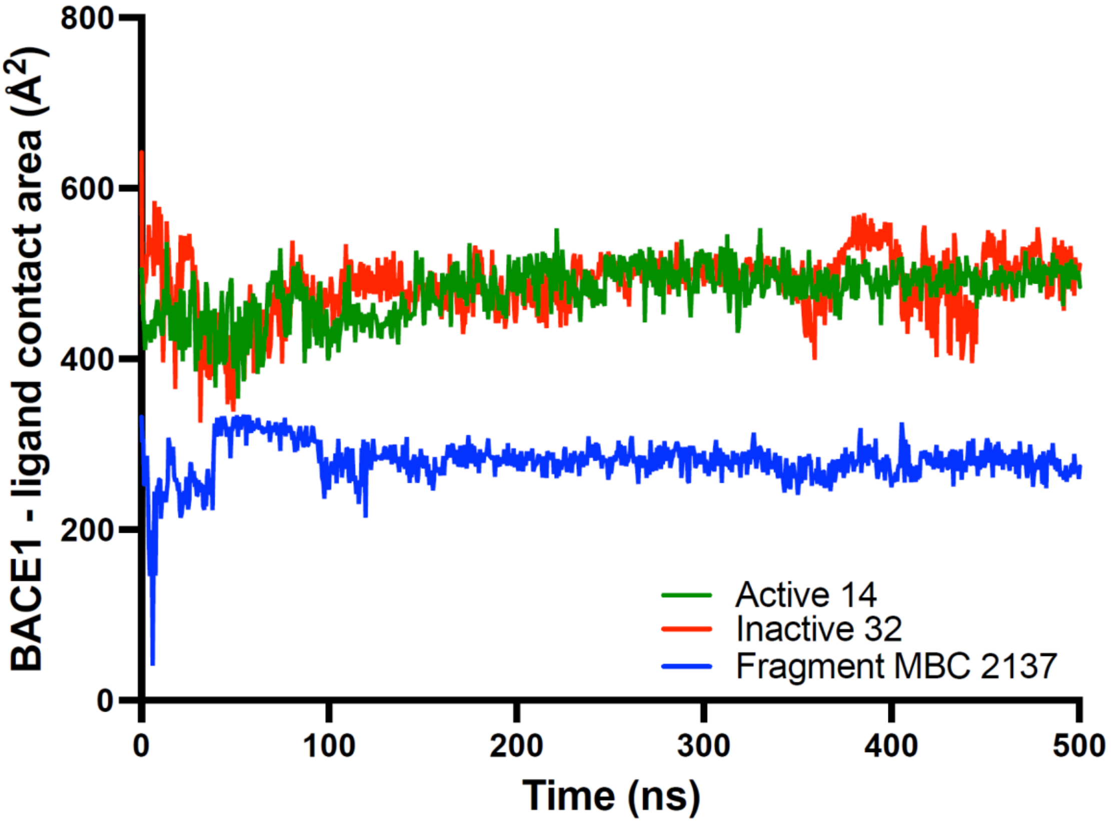

Figure S10. BACE1-ligand interaction contacts during the 500ns simulations. Green lines: pi-pi interactions; pink lines: H-bonds (and water bridges); red lines: pi-cation interactions. Hydrophobic, polar, and charged residues are displayed as green, cyan, and purple spheres. Interactions that occur more than 10% of the simulation time (500ns) are shown. More stable interactions are observed for compound **14** than for compound **32** and the MBC-2137 fragment. For example, Pi-Pi interactions with Tyr71, or hydrogen bonds with Thr232 remain for more than 50% of the simulation of compound **14** interacting with BACE1. This indicates that **14** stabilized very well in the binding site and that through these interactions it remains anchored in the pocket. For compound **32** there were no interactions above 30%, indicating that the compound moves in the large binding site and does not establish stable interactions throughout the simulation

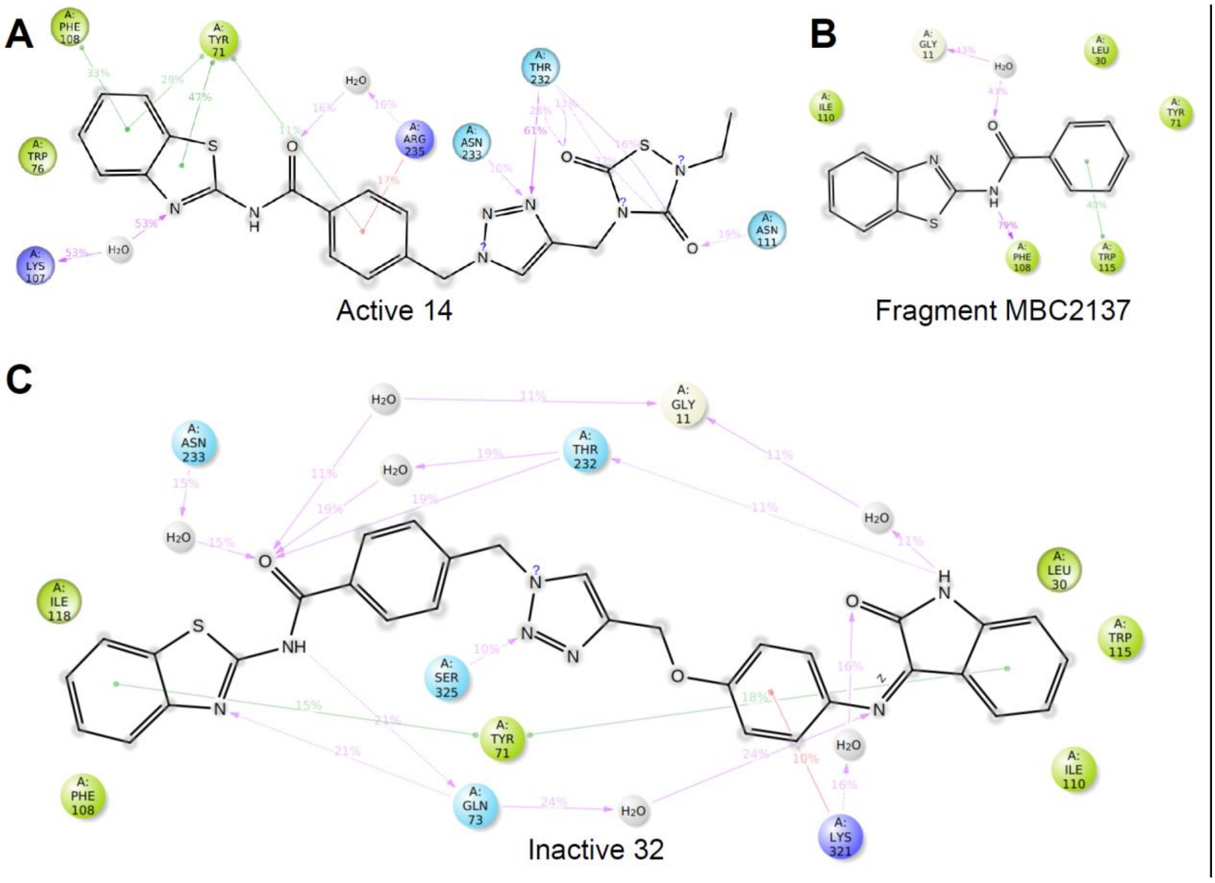

Figure S11. Time dependence of the RMSD for ligand atoms (red) and BACE1 backbone atoms (black) during the 500 ns unrestrained MDs. There are not significant changes in the BACE1 protein during simulations, which allows us to see that in all 3 simulations without energy restrictions the protein behaved stably. Regarding the changes in the atoms of the ligands. It is observed that the active compound **14** slowly changes it conformation in the binding site and after ~100ns it stabilizes and remains stable. Compound **32** undergoes several significant changes at the beginning of the simulation and after ~110 ns it stabilizes until ~350ns, where it undergoes another conformational change. Compound MCB-2137 rapidly changes conformation, moves around the large binding site at the beginning of the simulation, where it stabilizes after ~100ns.

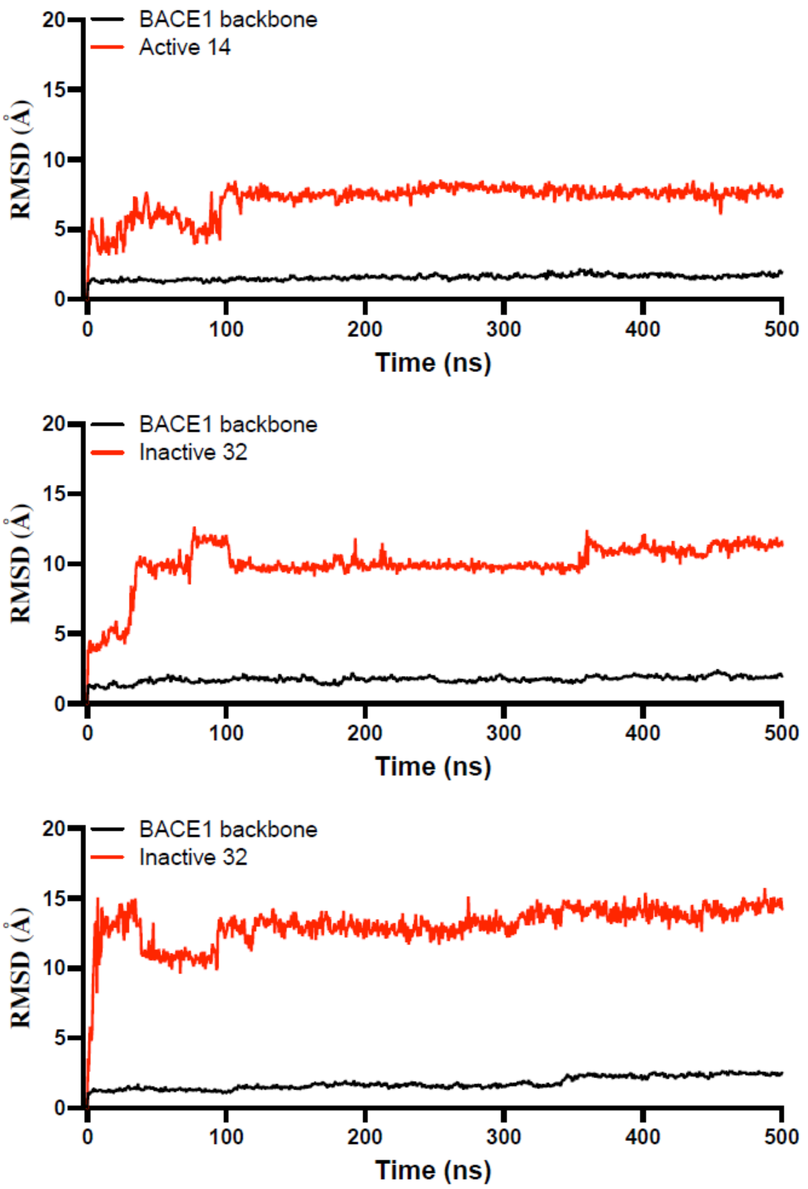

Figure S12. BACE1 – ligands interactions throughout the simulation. Interactions are categorized into five types: Hydrogen Bonds, Water Bridges, Hydrophobic, Pi-Pi, and Pi-cation. The stacked bar charts are normalized over the course of the trajectory: for example, a value of 0.5 suggests that 50% of the 500ns simulation (~250 ns) the specific interaction is maintained. Values over 1.0 indicated that the protein reside make multiple contacts of same subtype with the ligand. The active MTD compound **14** establish strong integrations with BACE1 residues at the binding site, manly with Tyr71, Lys107, and Thr232. These interactions possibly allow it to bind with higher affinity to BACE1 (-70.02 ± 9.90 kcal/mol), which is reflected in its gain of activity against the protein.

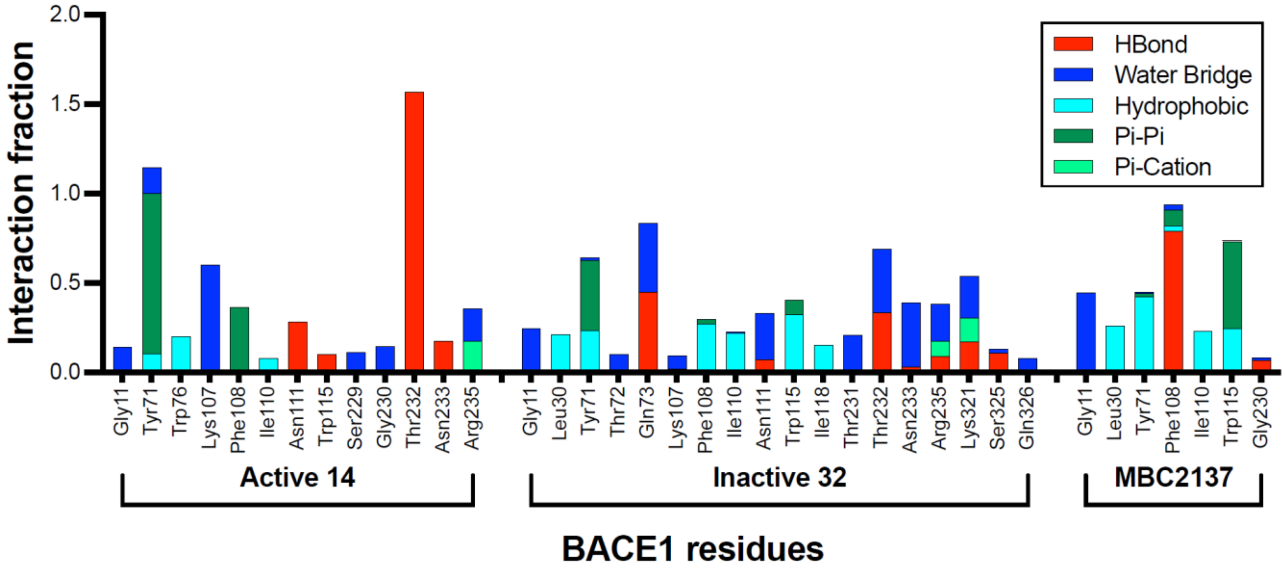

Figure S13. BACE1 – ligands interacting throughout the simulation. Compound **14** (A-C), compound **32** (D-F), and compound MBC-2137 (G-I) are shown as green sticks. Interacting residues are shown as blue sticks. Hydrogen bonds (blue), Pi-Pi (green), Pi-cation (yellow), and hydrophobic (gray) interactions are shown as dotted lines.

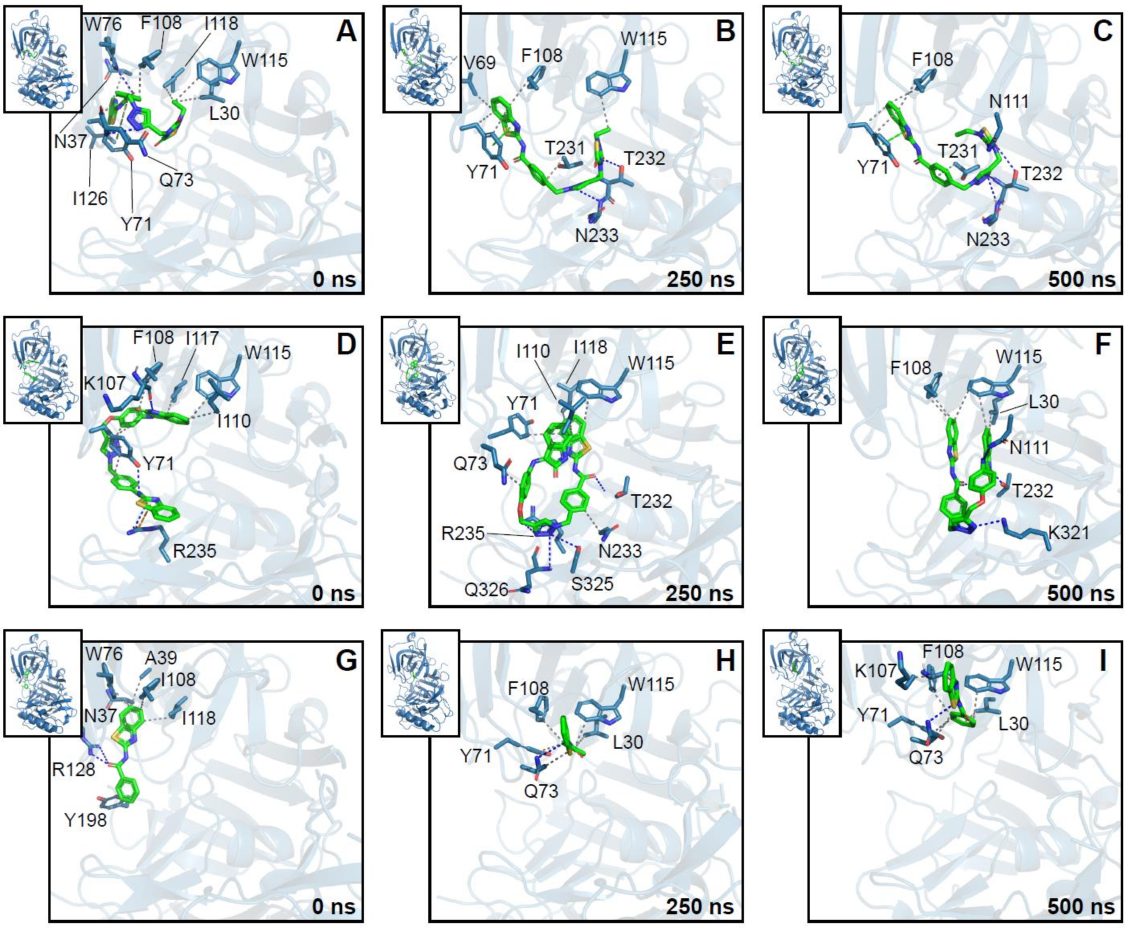

It is observed that the active compound **14** at 0 ns is located at the top of the binding site (Figure S13-A). After 250ns stable and strong interactions have already been established, mainly Pi-Pi interactions with the Y71 residue in the flap, and hydrogen bonds with NT232 and N233 at the bottom of the cavity (Figure S13-B). This allows compound **14** to spread throughout the cavity with high affinity, stabilized also by other hydrophobic interactions. After 500ns the binding mode of compound **14** remains stable (Figure S13-C). The inactive compound **32**, at the beginning of the simulation is interacting in the peripheral part (Figure S13-D), then it changes its conformation and moves towards the interior of the cavity (Figure S13-E), there it is observed that it folds in itself establishing intramolecular Pi-Pi interactions. Finally, it moves folded towards the bottom of the cavity and establishes mainly hydrophobic interactions. It does not interact with Y71 at the flap. The MBC2137 fragment at the beginning of the simulation (Figure S13-G) is found interacting at the top-peripheral part of the BACE1 binding site. Later it enters the binding pocket a little but always remains at the top (Figures S13-H and S13-I). Because it is a fragment of relatively small size (with respect to **14** and **32**) it is not possible for it to establish interactions along the cavity, this is reflected in its lack of activity and affinity for BACE1.

Chemistry: Experimental procedures

General Information

Reagents were obtained from the commercial sources and used without further purification. Purifications of crudes were performed with the indicated solvent as eluent by flash column chromatography carried out at medium pressure using silica gel (E. Merck, Grade 60, particle size 0.040–0.063 mm, 230–240 mesh ASTM) or IsoleraOne flash purification system from Biotage. ^{1}H NMR and ^{13}C NMR data were obtained from a Bruker AV300 or AV500 MHz spectrometer. Chemical shifts, δ, are expressed in ppm and calculated taking the reference of the appropriated deuterated solvents. Signal multiplicities (bs: broad signal, s: singlet, d: doublet, dd: doublet of doublets, ddd: doublet of doublet of doublets, t: triplet, td: triplet of doublets, q: quartet, m: multiplet) and coupling constants (*J* = Hz) are indicated for each molecule. Acquired spectroscopic data was analysed with MestreNova 10.2 software. The microwave assisted synthesis was carried out using a Biotage Initiator eight single-mode cavity instrument from Biotage. Experiments were performed with temperature control mode in sealed microwave process vials. The temperature was measured with an IR sensor on the outside of the reaction vessel. Stirring was provided by an *in situ* magnetic stirrer. High-Performance Liquid Chromatography (HPLC-MS) analysis. The column used for the analysis was a SunFire ® C18, 3.5µm, 4.6x50mm and UV-Vis spectra of the samples were acquired in a Thermo Finnigan Surveyor UV-Vis Plus Detector coupled with FinniganTM LXQ TM. Melting points were determined in a Büchi Melting Point M-560.

Experimental

4-(Bromomethyl)benzoyl chloride (1). 4-(bromomethyl)benzoic acid (500 mg, 2.3 mmol) and thionyl chloride (410 mg, 3.3 mmol) were dissolved in 2 mL of CH_2Cl_2. The reaction was stirred under inert atmosphere at 80ºC overnight. Then, the crude was rotated under reduced pressure to remove the excess of thionyl chloride to provide an orange solid which was used in the next reaction without further purification.

***N*-(benzo[d]thiazol-2-yl)-4-(bromomethyl)benzamide (2).** 4-(Bromomethyl)benzoyl chloride **(1)** (500 mg, 2.2 mmol) and 2-aminobenzothiazol (340 mg, 2.2 mmol) were dissolved in THF and stirred for 2 h under MW irradiation. The crude is dissolved in CH_2Cl_2, washed with HCl (1M), $NaHCO_3$ and brine. The organic layers were dried over $MgSO_4$, filtered, concentrated, and dried under high vacuum. The crude was purified by flash chromatography using ethyl acetate/hexane 7:1 as solvents to provide a white solid (300 mg, 39% yield). The presence of the compound was confirmed using HPLC-MS and it was used in the next reaction.

4,4'-((propane-1,3-diylbis(sulfanediyl))bis(methylene))bis(*N*-(benzo[d]thiazol-2-yl)benzamide) (3). To a solution of potassium carbonate (175 mg, 1.2 mmol) in THF propane-1,3-dithiol (23.4 mg, 0.2 mmol) was added. After 30 min. *N*-(benzo[d]thiazol-2-yl)-4-(bromomethyl)benzamide (2) (0.15 g, 0.4 mmol) in THF solution was added. The reaction was stirred at 80ºC for 46 h. The solvent was removed under reduced pressure to give a yellow crude which was dissolved in CH_2Cl_2 and washed with $NaHCO_3$ and brine. The organic phases were dried over $MgSO_4$, filtered, concentrated, and dried under high vacuum. The remaining solid was purified by flash chromatography using ethyl acetate/hexane as eluents to afford a white solid (97 mg, 72% yield). ^{1}H-NMR (300 MHz, $CDCl_3$) δ (ppm): 12.83 (s, 2H), 8.10 (d, J = 8.3 Hz, 4H), 8.00 (d, J = 7.3 Hz, 2H), 7.78 (d, J = 7.9 Hz, 2H), 7.56 – 7.39 (m, 6H), 7.33 (td, J = 7.4, 7.1, 1.3 Hz, 2H), 3.81 (s, 4H), 2.53 – 2.41 (m, 4H), 1.83 – 1.68 (m, 2H). ^{13}C NMR (75 MHz, DMSO-d_6) δ (ppm): 165.6, 148.9, 144.2, 132.0, 131.2, 129.0, 128.5, 126.2, 123.7, 121.7, 34.6, 29.6, 28.3. ESI calcd for $C_{33}H_{29}N_4O_2S_4$ $[M + H]^+$ 641.1168; found 641.1163.

4-(Azidomethyl)-*N*-(benzo[d]thiazol-2-yl)benzamide (4). 4-(Azidomethyl)benzoic acid (354 mg, 2.0 mmol), EDCI (383 mg, 4.0 mmol) and DMAP (488 mg, 2.0 mmol) were dissolved in DMF (10 mL). After stirring the mixture during 5 min. at r.t., 2-amino-benzothiazole (300 mg, 2.0 mmol) were added. The resulting mixture was stirred overnight at r.t.. Afterwards, water (35 mL) and ethyl acetate (35 mL) was added and the mixture was washed with saturated $NaHCO_3$ and saturated NaCl solutions. The solvent of the organic phase was then removed under reduced pressure and the remaining solid was purified by flash chromatography using ethyl acetate/hexane 1:9 as eluents to

afford a white solid (426 mg, 69% yield). ^{1}H NMR (300 MHz, DMSO-d_6) δ (ppm): 12.95 (s, 1H), 8.27 – 8.11 (d, *J* = 8.2 Hz, 2H), 8.03 (dd, *J* = 7.6, 1.2 Hz, 1H), 7.80 (d, *J* = 8.2 Hz, 1H), 7.56 (d, *J* = 8.2 Hz, 2H), 7.48 (dd, *J* = 8.2, 1.3 Hz, 1H), 7.35 (td, *J* = 7.6, 1.2 Hz, 1H), 4.60 (s, 2H). ^{13}C NMR (75 MHz, DMSO-d_6) δ (ppm): 166.1, 159.3, 148.9, 141.1, 131.2, 129.2, 128.8, 126.7, 124.2, 122.2, 120.8, 53.5. ESI calcd for $C_{15}H_{12}N_5OS$ [M + H]$^+$ 310.0757; found 310.0765.

Methyl-4-(prop-2-yn-1-yloxy)benzoate (5). 3-Bromopropyne (600 µL, 7.67 mmol) was added to a mixture of methyl 4-hydroxybenzoate (991 mg, 6.52 mmol) and potassium carbonate (3.47 g, 25.1 mmol) in acetonitrile (32 mL) via a syringe. The resulting solution was stirred overnight under reflux. After cooling to r.t.and quenching with water, the solvent was extracted with ethyl acetate (20 mL × 3). The combined organic layer was washed with brine (10 mL), dried over anhydrous Na_2SO_4 and concentrated to dryness. The isolated residue was further purified by silica gel chromatography to afford a colorless oil (929 mg, 75% yield). ^{1}H NMR (600 MHz, $CDCl_3$) δ (ppm): 8.08 (d, *J* = 9.0 Hz, 2H), 7.00 (d, *J* = 9.0 Hz, 2H), 4.75 (s, 2H), 3.89 (s, 3H), 2.55 (s, 1H). ^{13}C NMR (75 MHz, $CDCl_3$) δ (ppm): 172.6, 157.1, 130.7, 127.4, 115.4, 78.9, 75.9, 56.2, 52.4, 40.7.

4-(Prop-2-yn-1-yloxy)benzoic acid (6). Methyl 4-propynyloxybenzoate (**5**) (3.8 g, 20 mmol) was dissolved into CH_3OH (30 mL) and THF (1:1 v/v), then 5 mL NaOH (4 g, 100 mmol) aqueous solution was added dropwise. The mixture was stirred for 12 h at r. t.. After adjusted pH ≈ 3 with 5 mol/L HCl, the solvent was removed under reduced pressure. Then, 200 mL water was added and stirred for 0.5 h. The insoluble substance was filtered and washed with cold water. A white solid was obtained, (3.48 g, 98.8 % yield). ^{1}H NMR (300 MHz, DMSO-d_6) δ (ppm): 7.86 (d, *J* = 8.3 Hz, 2H), 7.01 (d, *J* = 8.3 Hz, 2H), 4.84 (s, 2H), 3.55 (t, 1H). ^{13}C NMR (75 MHz, DMSO-d_6) δ (ppm): 173.0, 155.9, 130.4, 127.8, 114.6, 79.4, 78.2, 55.3, 39.8.

***N*-(Benzo[d]thiazol-2-yl)-4-(prop-2-yn-1-yloxy)benzamide (7).** 4-(Prop-2-yn-1-yloxy)benzoic acid (**6**) (704 mg, 4.0 mmol), EDCI (766 mg, 4.0 mmol) and DMAP (976 mg, 8.0 mmol) were dissolved in DMF. After stirring the mixture during 5 min. at r.t., 2-amino-benzothiazole (600 mg, 4.0 mmol) was added. The resulting mixture was stirred overnight at r. t.. Afterwards, ethyl acetate (35 mL) was added

and the insoluble substance was filtered and washed with cold water to afford a white solid (852 mg, 69% yield). mp: 150-152 ºC. ^{1}H NMR (300 MHz, DMSO-d_6) δ (ppm): 12.77 (bs, 1H), 8.17 (d, *J* = 8.9 Hz, 2H), 8.02 (d, *J* = 7.6, 1H), 7.78 (d, *J* = 8.0 Hz, 1H), 7.47 (ddd, *J* = 8.2, 7.3, 1.3 Hz, 1H), 7.34 (td, *J* = 7.6, 1.2 Hz, 1H), 7.22 – 7.09 (m, 2H), 4.94 (d, *J* = 2.4 Hz, 2H), 3.66 (s, 1H). ^{13}C NMR (75 MHz, DMSO-d_6) δ (ppm): 165.6, 161.2, 159.3, 148.9, 132.0, 130.8, 126.6, 125.1, 124.0, 122.2, 120.7, 115.2, 79.2, 56.2. ESI calcd for $C_{17}H_{13}N_2O_2S$ [M + H]$^+$ 309,0692; found 309,0699.

2-(4-(Bromomethyl)phenyl)acetyl chloride (9). To a solution of 2-(4-(bromomethyl)phenyl)acetic acid (2.35 g, 10 mmol) in chloroform (15 mL) was added thionyl chloride (3.85 g, 32 mmol). The mixture was heated to 70 °C for 6 h to obtain a yellow solution. The mixture was cooled to r.t.and the solvent was removed under reduced pressure to give a yellow solid (2.37 g, 96% yield). This product was used in the next reaction without further purification.^{1}H NMR (400 MHz, $CDCl_3$) δ (ppm): 7.44 – 7.38 (m, 2H), 7.28 – 7.24 (m, 2H), 4.48 (s, 2H), 4.14 (s, 2H). ^{13}C NMR (100.6 MHz, $CDCl_3$) δ (ppm): 171.6, 137.8, 131.3, 129.9, 129.6, 52.6, 32.6.

***N*-(Benzo[d]thiazol-2-yl)-2-(4-(bromomethyl)phenyl)acetamide (10).** To a solution of aminobenzothiazole (600 mg, 4.0 mmol) in THF (7.5 mL) the acid chloride **9** (990 mg, 4.0 mmol) was added at r. t.. This mixture was heated under microwave radiation at 100 °C for 15 min. Then, the mixture was cooled to r.t., water (35 mL) and CH_2Cl_2 (65 mL) were added. The solvent of the organic phase was then removed under reduced pressure and the remaining solid was purified by flash chromatography using ethyl acetate/hexane 9:1 as eluents to afford a white solid used in the next reaction.

2-(4-(Azidomethyl)phenyl)-*N*-(benzo[d]thiazol-2-yl)acetamide (11). Sodium azide (0.042 g, 0.65 mmol) was added to a solution of *N*-(benzo[d]thiazol-2-yl)-2-(4-(bromomethyl)phenyl)acetamide, **10**, (0.150 g, 0.43 mmol) in DMSO (2 mL), and the reaction mixture was stirred for 18 h at r. t.. The reaction mixture was quenched by adding water (5 mL) and extracted with ethyl ether (20 mL x 3). The organic layers were dried over $MgSO_4$, filtered, concentrated, and dried under high vacuum. The remaining solid was purified by flash chromatography using

ethyl acetate/hexane 9:1 as eluents to afford a white solid (109 mg, 79% yield). ^{1}H NMR (300 MHz, $CDCl_3$) δ (ppm): 9.92 (bs, 1H), 7.77 (d, *J* = 7.7 Hz, 1H), 7.66 (d, *J* = 8.1 Hz, 1H), 7.37 (ddd, *J* = 8.2, 7.3, 1.3 Hz, 1H), 7.26 (td, *J* = 7.7, 1.2 Hz, 1H), 7.22 – 7.12 (m, 4H), 4.26 (s, 2H), 3.77 (s, 2H). ^{13}C NMR (75 MHz, $CDCl_3$) δ (ppm): 169.5, 159.0, 148.3, 135.7, 133.2, 132.4, 130.3, 129.3, 126.9, 124.6, 122.0, 121.1, 54.7, 43.5. ESI calcd for $C_{16}H_{14}N_5OS$ $[M + H]^+$ 324.0814; found 324.0814.

3-Ethyl-5-(prop-2-yn-1-yl)thiazolidine-2,4-dione (12). A stirred solution of ethyl isocyanate (163 µL, 2.0 mmol) and propargyl isothiocyanate (189 µL, 2.0 mmol) in THF (5 mL) was cooled to 0 °C. Sulfuryl chloride (167 µL, 2 mmol) was added slowly and the mixture was allowed to warm to r.t.and stirred overnight. The reaction was then opened to the air and stirred for 30 minutes before the solvent was removed under reduced pressure. Flash chromatography (0-20% ethyl acetate in hexane) was used to purify the crude reaction mixture, to obtain a pale yellow oil (243 mg, 66% yield). ^{1}H NMR (300 MHz, $CDCl_3$) δ (ppm): 4.37 (d, *J* = 2.5 Hz, 2H), 3.65 (q, *J* = 7.2 Hz, 2H), 2.23 (t, *J* = 2.7 Hz, 1H), 1.22 (t, *J* = 7.2 Hz, 3H). ^{13}C NMR (75 MHz, $CDCl_3$) δ (ppm): 165.5, 152.0, 76.5, 72.8, 40.6, 31.8, 14.3. ESI calcd for $C_7H_9N_2O_2S$ $[M + H]^+$ 185.0379; found 185.0385.

(*Z*)-3-((4-(prop-2-yn-1-yloxy)phenyl)imino)indolin-2-one (13). A mixture of isatin (294 mg, 2 mmol) and 4-(prop-2-yn-1-yloxy)aniline (294 mg, 2 mmol) in ethanol (3 mL) was refluxed on a steam bath for 1 h and stirred at r.t.for 12 h. The product obtained was filtered and the solid was purified by silica gel chromatography with ethyl acetate and hexane 1:1 to obtain an orange solid (110 mg, 20% yield). mp: 230 – 232 ºC. ^{1}H NMR (300 MHz, DMSO-d_6) δ (ppm): 10.97 (s, 1H), 7.35 (td, *J* = 7.7, 1.3 Hz, 1H), 7.13 – 7.08 (m, 2H), 7.03 – 6.97 (m, 2H), 6.90 (d, *J* = 7.8 Hz, 1H), 6.76 (td, *J* = 7.7, 1.0 Hz, 1H), 6.59 (dd, *J* = 7.8, 1.2 Hz, 1H), 4.85 (d, *J* = 2.4 Hz, 2H), 3.62 (t, *J* = 2.4 Hz, 1H). ^{13}C NMR (75 MHz, DMSO-d_6) δ (ppm): 164.1, 155.4, 155.3, 147.3, 144.3, 134.7, 125.5, 122.2, 119.7, 116.3, 116.2, 111.9, 79.7, 78.8, 56.3. ESI calcd for $C_{17}H_{13}N_2O_2$ $[M + H]^+$ 277.0972; found 277.0979.

Methyl 2-(4-(prop-2-yn-1-yloxy)phenyl)acetate (18). Methyl 2-(4-hydroxyphenyl)acetate (1.0 g, 6 mmol) and potassium carbonate (2.5 g, 18 mmol)

were dissolved in 20 mL of anhydrous THF and the mixture was stirred under inert atmosphere at 80 ºC for 30 min. Then propargyl bromide (1.8 g, 15 mmol) was added and the reaction was stirred at r.t. overnight. The crude was extracted with ethyl acetate (20 mL) and the organic phase was washed with brine, dried over $MgSO_4$, filtered and concentrated to dryness. The isolated residue was further purified by sillica gel chromatography to obtain a colorless oil (883 mg, 77% yield). ^1H NMR (300 MHz, $CDCl_3$) δ (ppm): 7.21 (d, *J* = 8.6 Hz, 2H), 6.94 (d, *J* = 8.3 Hz, 2H), 4.68 (d, *J* = 2.3 Hz, 2H), 3.68 (s, 3H), 3.57 (s, 2H), 2.51 (t, *J* = 2.5 Hz, 1H). ^{13}C NMR (75 MHz, $CDCl_3$) δ (ppm): 172.6, 157.1, 130.7, 127.4, 115.4, 78.9, 75.9, 56.2, 52.4, 40.7.

2-(4-(Prop-2-yn-1-yloxy)phenyl)acetic acid (19). A solution of sodium hydroxide (0.5 g, 12 mmol) in water (4 mL) was added to a solution of methyl 2-(4-(prop-2-yn-1-yloxy)phenyl)acetate (**18**) (0.8 g, 4 mmol) in THF (16 mL). The mixture was stirred at r.t.overnight. The pH was adjusted to ≈ 3 with 5 mol/L HCl and the crude was extracted with ethyl acetate. The organic layers were washed with H_2O and brine, dried over $MgSO_4$, filtered and concentrated to dryness to obtain a white solid without further purification (533 mg, 70% yield). ^1H NMR (300 MHz, $CDCl_3$) δ (ppm): 12.56 (s, 1H), 7.18 (d, *J* = 8.6 Hz, 2H), 6.92 (d, *J* = 8.6 Hz, 2H), 4.76 (d, *J* = 2.4 Hz, 2H), 3.55 (t, *J* = 2.4 Hz, 1H), 3.49 (s, 2H). ^{13}C NMR (75 MHz, $CDCl_3$) δ (ppm): 172.9, 155.9, 130.4, 127.8, 114.6, 79.4, 78.2, 55.3, 39.8.

***N*-(Benzo[d]thiazol-2-yl)-2-(4-(prop-2-yn-1-yloxy)phenyl)acetamide (20).** 2-(4-(Prop-2-yn-1-yloxy)phenyl)acetic acid (**19**) (0.25 g, 1.3 mmol) were dissolved in anhydrous CH_2Cl_2 and $SOCl_2$ (150 µL, 2.0 mmol) was added dropwise. The mixture was stirred at 80 ºC in an inert atmosphere for 24 hours. The crude was dried at vaccum pressure to eliminate the excess of $SOCl_2$ and used in the following reaction. The acid chloride previously synthesized was dissolved with THF (2.5 mL) and of 2-aminobenzothiazole (0.15 g, 1 mmol) in a microwave vial. The reaction was stirred under microwave radiation for 2 hours at 110ºC. The crude was extracted with CH_2Cl_2 (20 mL) and the organic layer was washed with brine. The isolated residue was purified by silica gel chromatography to give a white solid (180 mg, 60% yield), mp. 166 - 167 ºC. ^1H NMR (300 MHz, $CDCl_3$) δ (ppm): 9.30 (s, 1H), 7.76 (ddd, *J* = 8.0, 1.3, 0.7 Hz, 1H), 7.68 (dt, *J* = 8.1, 0.9 Hz, 1H), 7.43 (ddd, *J* = 8.2, 7.3, 1.3 Hz, 1H), 7.32 (ddd, *J* = 8.4, 7.3, 1.2 Hz, 1H), 7.23

(d, J = 8.6 Hz, 2H), 6.94 (d, J = 8.7 Hz, 2H), 4.70 (d, J = 2.4 Hz, 2H), 3.81 (s, 2H), 2.55 (s, 1H). ^{13}C NMR (75 MHz, $CDCl_3$) δ (ppm): 169.6, 158.0, 157.6, 148.3, 132.3, 130.9, 126.5, 125.5, 124.2, 121.6, 121.0, 116.0, 78.4, 76.0, 56.0, 42.9. ESI calcd for $C_{18}H_{15}N_2O_2S$ [M + H]$^+$ 323.0849; found 323.0847.

2-(4-(Prop-2-yn-1-yloxy)phenyl)-*N*-(6-(trifluoromethyl)benzo[d]thiazol-2-yl)acetamide (21). 2-(4-(Prop-2-yn-1-yloxy)phenyl)acetic acid (**19**) (0.25 g, 1.3 mmol) were dissolved in anhydrous CH_2Cl_2 and $SOCl_2$ (150 µL) was added dropwise. The mixture was stirred at 80 ºC in an inert atmosphere for 24 hours. The crude was dried under vaccum to eliminate the excess of $SOCl_2$ and used in the following reaction. The acid chloride previously synthesized was dissolved with THF (2.5 mL) and 2-amino-6-trifluoromethylbenzothiazole (0.23 g, 1 mmol) in a microwave vial. The reaction was stirred under microwave radiation for 2 hours at 110ºC. The crude was extracted with CH_2Cl_2 (20 mL) and the organic layer was washed with brine. The isolated residue was purified by silica gel chromatography to give a white solid (237 mg, 61% yield), mp. 127-128 ºC. ^{1}H NMR (300 MHz, $CDCl_3$) δ (ppm): 9.39 (s, 1H), 8.10 (dt, J = 1.7, 0.8 Hz, 1H), 7.78 (dt, J = 8.5, 0.8 Hz, 1H), 7.66 (ddd, J = 8.5, 1.8, 0.7 Hz, 1H), 7.22 (d, J = 8.7 Hz, 2H), 7.00 (d, J = 8.7 Hz, 2H), 4.71 (d, J = 2.4 Hz, 2H), 3.90 (s, 2H), 2.55 (t, J = 2.4 Hz, 1H). ^{13}C NMR (75 MHz, $CDCl_3$) δ (ppm): 170.1, 161.7, 157.8, 146.5, 131.1, 130.6, 127.8, 126.20 (d, $J_{C\text{-}F}$ = 271.6 Hz), 124.8, 124.52 (d, $J_{C\text{-}F}$ = 3.0 Hz), 120.0, 119.73 (d, $J_{C\text{-}F}$ = 4.1 Hz), 116.1, 78.4, 76.0, 56.0, 42.9. ESI calcd for $C_{19}H_{14}FN_2O_2S$ [M + H]$^+$ 391.0723; found 391.0721.

***N*-(6-methoxybenzo[d]thiazol-2-yl)-2-(4-(prop-2-yn-1-yloxy)phenyl)-acetamide (22).** 2-(4-(Prop-2-yn-1-yloxy)phenyl)acetic acid (**19**) (0.24 g, 1.1 mmol) were dissolved in anhydrous CH_2Cl_2 and 150 µL of $SOCl_2$ were added dropwise. The mixture was stirred at 80 ºC in an inert atmosphere for 24 hours. The crude was dried under vaccum to eliminate the excess of $SOCl_2$ and used in the following reaction. The acid chloride previously synthesized was dissolved with THF (2.5 mL) and 2-amino-6-methoxybenzothiazole (0.22 g, 1.1 mmol) in a microwave vial. The reaction was stirred under microwave radiation for 2 hours at 110 ºC. The crude was extracted with CH_2Cl_2 (20 mL) and the organic layer was washed with brine. The isolated residue was purified by silica gel chromatography to give a white solid (200 mg, 51% yield), mp. 134-135 ºC. ^{1}H

NMR (300 MHz, $CDCl_3$) δ (ppm): >12.5 (s, 1H), 7.54 (d, *J* = 8.9 Hz, 1H), 7.27 – 7.13 (m, 4H), 6.99 (dd, *J* = 8.9, 2.5 Hz, 1H), 6.92 (d, *J* = 8.7 Hz, 2H), 4.72 (d, *J* = 2.4 Hz, 2H), 3.80 (s, 3H), 3.74 (s, 2H), 2.48 (s, 1H). ^{13}C NMR (75 MHz, $CDCl_3$) δ (ppm): 169.5, 157.5, 157.2, 156.5, 141.3, 133.0, 130.9, 125.5, 121.2, 116.0, 115.7, 104.5, 78.4, 76.0, 56.0 42.9. ESI calcd for $C_{19}H_{17}N_2O_2S$ [M + H]$^+$ 353.0954; found 353.0954.

Ethyl 4-methyl-2-(2-(4-(prop-2-yn-1-yloxy)phenyl)acetamido)thiazole-5-carboxylate (23). 2-(4-(Prop-2-yn-1-yloxy)phenyl)acetic acid (**19**) (0.72 g, 11 mmol) were dissolved in anhydrous CH_2Cl_2 and of $SOCl_2$ (120 µL, 1.5 mmol) were added dropwise. The mixture was stirred at 80 ºC in an inert atmosphere for 24 hours. The crude was dried dried under vaccum to eliminate the excess of $SOCl_2$ and used in the following reaction. The acid chloride previously synthesized was dissolved with THF (2.5 mL) and ethyl 2-amino-4-methylthiazole-5-carboxylate (0.20 g, 1 mmol) in a microwave vial. The reaction was stirred under microwave radiation for 2 hours at 110ºC. The crude was extracted with CH_2Cl_2 (20 mL) and the organic layer was washed with brine. The isolated residue was purified by silica gel chromatography to give a white solid (258 mg, 72% yield), mp. 208-209 ºC. ^1H NMR (500 MHz, DMSO-d_6) δ (ppm): 12.68 (s, 1H), 7.25 (d, *J* = 8.6 Hz, 2H), 6.94 (d, *J* = 8.7 Hz, 2H), 4.77 (d, *J* = 2.4 Hz, 2H), 4.22 (q, *J* = 7.1 Hz, 2H), 3.72 (s, 2H), 3.54 (t, *J* = 2.4 Hz, 1H), 2.50 (s, 3H), 1.26 (t, *J* = 7.1 Hz, 3H). ^{13}C-NMR (126 MHz, DMSO-d_6) δ (ppm): 170.8, 162.5, 160.0, 156.7, 156.6, 130.7, 127.6, 115.3, 114.5, 79.8, 78.6, 61.0, 55.8, 41.2, 17.5, 14.7. ESI calcd for $C_{18}H_{19}N_2O_4S$ [M + H]$^+$ 359.1060; found 359.1048.

Ethyl 4-hydroxy-2-oxo-1-(prop-2-yn-1-yl)-1,2-dihydroquinoline-3-carboxylate (24). Sodium hydride (0.30 g, 11 mmol) and propargyl bromide (1.3 g, 11 mmol) were added to a solution of isatoic anhydride (1 g, 6.3 mmol) in DMF (20 mL) at 0 ºC. After the addition the reaction was stirred for 5 h at r.t. The crude was rotated at vacuum to evaporate the excess of propargyl bromide and used in the following reaction. The crude was mixed with ethyl malonate (7.6 mmol) and sodium hydride (7.6 mmol) and stirred for 12 h at 85 ºC. The mixture was acidified with HCl (1M) until the apparition of a precipitate, filtered under vacuum. The solid was washed with cold MeOH to afford a white solid (957 mg, 56% yield).

This compound was used in the following synthetic step without further characterization.

***N'*-dodecanoyl-4-hydroxy-1-(prop-2-yn-1-yl)-1,2-dihydroquinoline-3-carbohydrazide (25).** Ethyl 4-hydroxy-1-(prop-2-yn-1-yl)-1,2-dihydroquinoline-3-carboxylate (**23**) (0.6 g, 2.1 mmol) and dodecyl hydrazide (0.45 g, 2.2 mmol) were dissolved in DMF (15 mL). The mixture was stirred at 60 ºC for 3 min. and then it was left at r.t. MeOH (10 mL) was added observing the apparition of a white precipitate that was washed with cold MeOH (502 mg, 52% yield), mp. 163 - 164 ºC. ^{1}H NMR (500 MHz, DMSO-*d₆*) δ (ppm): 11.81 (d, *J* = 4.1 Hz, 1H), 10.78 (d, *J* = 4.2 Hz, 1H), 8.15 (dd, *J* = 8.0, 1.6 Hz, 1H), 7.90 (ddd, *J* = 8.8, 7.1, 1.6 Hz, 1H), 7.71 (d, *J* = 8.7 Hz, 1H), 7.45 (t, *J* = 7.6 Hz, 1H), 5.14 (d, *J* = 2.5 Hz, 2H), 3.31 (t, *J* = 2.4 Hz, 1H), 2.24 (t, *J* = 7.4 Hz, 2H), 1.55 (t, *J* = 7.1 Hz, 2H), 1.34 – 1.19 (m, 19H), 0.93 – 0.76 (m, 3H). ^{13}C NMR (126 MHz, DMSO-d_6) δ (ppm): 171.4, 169.6, 167.1, 160.4, 138.4, 134.8, 124.7, 123.2, 115.7, 115.1, 95.4, 78.7, 74.9, 32.9, 31.3, 30.7, 29.0, 28.9, 28.7, 28.5, 25.0, 22.1, 14.0. ESI calcd for $C_{25}H_{36}N_3O_4$ [M + H]$^+$ 440.2544; found 440.2539.

4-(2-Azidoethyl)benzoic acid (26). 4-(2-Bromoethyl)benzoic acid (1.0 g, 4.4 mmol) and sodium azide (0.72 g, 11 mmol) were dissolved in DMF (10 mL). The reaction was stirred at 80ºC for 12 hours. The solvent was evaporated and the crude was resuspended in cold water and acidified with glacial acetic acid. The solid was dried at vacuum to give a white solid (574 mg, 68% yield). The compound was used in the next reaction without further purification.

4-(2-Azidoethyl)-*N*-(benzo[d]thiazol-2-yl)benzamide (27). 4-(2-Azidoethyl)benzoic acid (0.57 g, 3 mmol), 2-aminobenzothiazole (0.45 g, 3 mmol), DMAP (0.73 g, 6 mmol) and EDCI (0.63 g, 3.3 mmol) were dissolved in DMF and stirred for 12 h. at r.t.. Then the mixture was extracted with CH_2Cl_2 and water. The organic phase was dried over $MgSO_4$ and purified by silica gel chromatography. ^{1}H NMR (300 MHz, DMSO-d_6) δ (ppm): 12.85 (s, 1H), 8.11 (d, *J* = 8.3 Hz, 2H), 8.03 (dd, *J* = 8.0, 1.2 Hz, 1H), 7.79 (d, *J* = 8.0 Hz, 1H), 7.54 – 7.43 (m, 3H), 7.34 (td, *J* = 7.6, 1.2 Hz, 1H), 3.65 (t, *J* = 7.0 Hz, 2H), 2.96 (t, *J* = 7.0 Hz, 2H). ^{13}C NMR (75 MHz, DMSO-d_6) δ (ppm): 166.2, 159.3, 148.9, 144.3,

132.0, 130.6, 129.6, 128.9, 126.6, 124.1, 122.2, 120.8, 51.5, 34.8. ESI calcd for $C_{16}H_{14}N_5OS$ [M + H]$^+$ 324.0914; found 324.0912.

General procedure for the synthesis of triazoles (8, 14-17, 28-33):

A mixture of alkyne (1 eq) and azide (1 eq) in DMF was reacted overnight at r.t.in presence of copper sulfate ($CuSO_4{\cdot}5H_2O$) (10 mol%) and sodium ascorbate (20 mol%). The reaction mixture was poured onto water, extracted with CH_2Cl_2:MeOH (9:1), washed with 5% ammonium hydroxide solution, dried over magnesium sulfate ($MgSO_4$), concentrated and purified using flash chromatography on silica gel (CH_2Cl_2 - MeOH mixture).

***N*-(Benzo[d]thiazol-2-yl)-4-((1-(4-(benzo[d]thiazol-2-ylcarbamoyl)benzyl)-1H-1,2,3-triazol-4-yl)methoxy)benzamide (8).** *N*-(benzo[d]thiazol-2-yl)-4-(prop-2-yn-1-yloxy)benzamide (**7**) (58.8 mg, 0.2 mmol) and 4-(azidomethyl)-*N*-(benzo[d]thiazol-2-yl)benzamide (**4**) (59.0 mg, 0.2 mmol) were used. Yield: 71 mg, 57%. White solid; mp: 280 – 282 ºC (decomp.). ^{1}H NMR (300 MHz, DMSO-d_6) δ (ppm): >12.5 (s, 1H), 10.95 (s, 1H), 8.23 (s, 1H), 8.07 (d, *J* = 7.8 Hz, 3H), 8.01 (d, *J* = 7.8 Hz, 1H), 7.79 (d, *J* = 8.0 Hz, 1H), 7.52 – 7.33 (m, 4H), 7.13 (d, *J* = 8.7 Hz, 2H), 7.01 – 6.94 (m, 2H), 6.88 (d, *J* = 7.8 Hz, 1H), 6.75 (t, *J* = 7.6 Hz, 1H), 6.62 (d, *J* = 7.6 Hz, 1H), 5.15 (d, *J* = 6.6 Hz, 2H), 4.73 (t, *J* = 7.1 Hz, 2H). ^{13}C NMR (75 MHz, DMSO-d_6) δ (ppm): 166.0, 165.5, 162.0, 159.30, 159.29, 148.8 (2C), 143.0, 141.1, 132.1, 131.9, 130.8, 129.2, 128.4, 126.6, 126.5, 125.6, 124.6, 124.6, 124.1, 123.9, 122.1, 122.0, 120.7, 115.0, 61.7, 52.8. ESI calcd for $C_{32}H_{24}N_7O_3S_2$ [M + H]$^+$ 618,1377; found 618,1385.

***N*-(Benzo[d]thiazol-2-yl)-4-((4-((2-ethyl-3,5-dioxo-1,2,4-thiadiazolidin-4-yl)methyl)-1H-1,2,3-triazol-1-yl)methyl)benzamide (14).** 4-(azidomethyl)-*N*-(benzo[d]thiazol-2-yl)benzamide (**4**) (59 mg, 0.2 mmol) and 3-ethyl-5-(prop-2-yn-1-yl)thiazolidine-2,4-dione (**12**) (37 mg, 0.2 mmol) were used. Yield: 66 mg, 67%. White solid; mp: 204 – 206 ºC.^{1}H NMR (300 MHz, DMSO-d_6) δ (ppm): 12.92 (s, 1H), 8.23 (s, 1H), 8.14 (d, *J* = 8.3 Hz, 2H,), 8.03 (d, *J* = 7.6, 1H), 7.79 (d, *J* = 8.0 Hz, 1H), 7.56 – 7.41 (m, 3H), 7.35 (td, *J* = 7.6, 1.2 Hz, 1H), 5.71 (s, 2H), 4.83 (s, 2H), 3.64 (q, *J* = 7.1 Hz, 2H), 1.16 (t, *J* = 7.1 Hz, 3H). ^{13}C NMR (75 MHz, DMSO-d_6) δ (ppm): 166.2, 152.5, 148.9, 142.1, 141.2, 132.1, 131.9, 129.3, 128.5, 126.7,

124.6, 124.2, 122.3, 120.8, 52.8, 40.1, 37.5, 14.0. ESI calcd for $C_{22}H_{20}N_7O_3S_2$ [M + H]$^+$ 494,1064; found 494,1072.

***N*-(Benzo[d]thiazol-2-yl)-2-(4-((4-((2-ethyl-3,5-dioxo-1,2,4-thiadiazolidin-4-yl)methyl)-1H-1,2,3-triazol-1-yl)methyl)phenyl)acetamide (15).** 2-(4-(azidomethyl)phenyl)-*N*-(benzo[d]thiazol-2-yl)acetamide (**11**) (64 mg, 0.2 mmol) and 3-ethyl-5-(prop-2-yn-1-yl)thiazolidine-2,4-dione (**12**) (37 mg, 0.2 mmol) were used. Yield: 62 mg, 61%. White solid; mp: 226 – 228 ºC (decomp.). ^{1}H NMR (300 MHz, DMSO-d_6) δ (ppm): 12.62 (s, 1H), 8.14 (s, 1H), 7.96 (d, *J* = 8.0 Hz, 1H), 7.75 (d, *J* = 8.0 Hz, 1H), 7.43 (td, *J* = 7.6, 1.2 Hz, 1H), 7.36 (d, *J* = 8.3 Hz, 2H), 7.35 – 7.23 (m, 3H), 5.57 (s, 2H), 4.81 (s, 2H), 3.85 (s, 2H), 3.61 (q, *J* = 7.1 Hz, 2H), 1.13 (t, *J* = 7.2 Hz, 3H). ^{13}C NMR (75 MHz, DMSO-d_6) δ (ppm): 170.5, 166.1, 158.4, 152.5, 149.0, 142.0, 135.1, 135.1, 131.9, 130.2, 128.6, 126.6, 124.2, 124.0, 122.2, 121.0, 53.0, 41.9, 40.0, 37.5, 14.0. ESI calcd for $C_{23}H_{21}N_7O_3S_2$ [M]$^+$ 507,1147; found 507,1156.

(*Z*)-*N*-(Benzo[d]thiazol-2-yl)-2-(4-((4-((4-((2-oxoindolin-3-ylidene)amino) phenoxy)methyl)-1H-1,2,3-triazol-1-yl)methyl)phenyl)-acetamide (16). 2-(4-(azidomethyl)phenyl)-*N*-(benzo[d]thiazol-2-yl)acetamide, (**11**) (64 mg, 0.2 mmol) and (*Z*)-3-(4-(prop-2-yn-1-yloxy)benzylidene)indolin-2-one (**13**) (62 mg, 0.2 mmol) were used. Yield: 55 mg, 46%. Orange solid; mp: 166 – 168 ºC. ^{1}H NMR (300 MHz, DMSO-d_6) δ (ppm): >12.5 (s, 1H), 10.89 (s, 1H), 8.25 (s, 1H), 7.95 – 7.78 (m, 2H), 7.67 (d, *J* = 8.0 Hz, 1H), 7.42 – 7.17 (m, 6H), 7.06 (d, *J* = 8.8 Hz, 2H), 6.94 – 6.86 (m, 2H), 6.82 (d, *J* = 7.8 Hz, 1H), 6.68 (t, *J* = 7.6 Hz, 1H), 6.54 (d, *J* = 7.6 Hz, 1H), 5.54 (s, 2H), 5.10 (s, 2H), 3.77 (s, 2H). ^{13}C NMR (75 MHz, DMSO-d_6) δ (ppm): 170.4, 162.7, 158.2, 156.2, 155.0, 148.8, 147.2, 143.8, 135.0, 134.6, 131.8, 130.2, 128.5, 126.5, 125.4, 125.1, 123.9, 122.1, 119.7, 115.9, 111.8, 61.71, 53.0, 41.8. ESI calcd for $C_{33}H_{26}N_7O_3S$ [M + H]$^+$ 600,6765; found 600,6774.

***N*-(Benzo[d]thiazol-2-yl)-4-((1-(4-(2-(benzo[d]thiazol-2-ylamino)-2-oxoethyl) benzyl)-1H-1,2,3-triazol-4-yl)methoxy)benzamide (17).** 2-(4-(Azidomethyl) phenyl)-*N*-(benzo[d]thiazol-2-yl)acetamide (**11**) (64 mg, 0.2 mmol) and 4-(azidomethyl)-*N*-(benzo[d]thiazol-2-yl)benzamide (**7**) (59.0 mg, 0.2 mmol) were used. Yield: 69 mg, 55%. White solid; mp: 198 – 200 ºC (decomp.). ^{1}H NMR (300

MHz, DMSO-d_6) δ (ppm): 12.57 (s, 2H), 8.33 (s, 1H), 8.15 (d, *J* = 8.4 Hz, 2H), 8.05 – 7.91 (m, 2H), 7.80 – 7.65 (m, 2H), 7.49 – 7.24 (m, 8H), 7.22 (d, *J* = 8.4 Hz, 2H), 5.61 (s, 2H), 5.25 (s, 2H), 3.84 (s, 2H). ^{13}C NMR (75 MHz, DMSO-d_6) δ (ppm): 170.5, 165.7, 162.8, 162.1, 158.3, 148.9, 143.0, 135.2, 135.1, 131.9, 130.9, 130.3, 128.6, 126.6, 125.3, 124.7, 124.0, 122.2, 121.0, 120.7, 115.1, 61.8, 53.1, 41.9. ESI calcd for $C_{33}H_{26}N_7O_3S$ [M + H]$^+$ 632,1533; found 632,1542.

***N*-(Benzo[d]thiazol-2-yl)-4-((4-((4-(2-oxo-2-((6-(trifluoromethyl)benzo[d]thiazol-2-yl)amino)ethyl)phenoxy)methyl)-1H-1,2,3-triazol-1-yl)methyl)benzamide (28).** 2-(4-(Prop-2-yn-1-yloxy)phenyl)-*N*-(6-(trifluoromethyl)benzo[d]thiazol-2-yl)acetamide (**21**) (47 mg, 0.12 mmol) and 4-(azidomethyl)-*N*-(benzo[d]thiazol-2-yl)benzamide (**4**) (38 mg, 0.12 mmol) were used. Yield 81.2 mg, 97%. Beige solid; mp: 210 – 212 ºC (decomp.). ^{1}H-NMR (500 MHz, DMSO-d_6) δ (ppm): 12.90 (s, 1H), 12.80 (s, 1H), 8.48 (s, 1H), 8.35 (s, 1H), 8.13 (d, *J* = 8.3 Hz, 2H), 8.02 (d, *J* = 7.9 Hz, 1H), 7.90 (d, *J* = 8.5 Hz, 1H), 7.78 (d, *J* = 8.1 Hz, 1H), 7.73 (dd, *J* = 8.6, 1.9 Hz, 1H), 7.52 – 7.43 (m, 3H), 7.34 (td, *J* = 7.9, 1.1 Hz, 1H), 7.28 (d, *J* = 8.7 Hz, 2H), 7.02 (d, *J* = 8.7 Hz, 2H), 5.74 (s, 2H), 5.15 (s, 2H), 3.80 (s, 2H). ^{13}C-NMR (126 MHz, DMSO-d_6) δ (ppm): 170.9, 161.2, 157.1, 151.3, 143.1, 140.8, 132.0, 131.7, 130.4, 128.8, 128.0, 126.7, 126.2, 125.6, 124.9, 123,7, 123.5 (d, $J_{C\text{-}F}$ = 10.3 Hz) 122.9 (d, $J_{C\text{-}F}$ = 3.7 Hz), 122.4, 121.8, 120.9, 119.9 (d, $J_{C\text{-}F}$ = 4.7 Hz), 114.7, 61.1, 52.4, 40.9. ESI calcd for $C_{34}H_{25}F_3N_7O_3S_2$ [M + H]+ 700,1407; found 700.1403.

***N*-(Benzo[d]thiazol-2-yl)-4-(2-(4-((4-(2-((6-methoxybenzo[d]thiazol-2-yl)amino)-2-oxoethyl)phenoxy)methyl)-1H-1,2,3-triazol-1-yl)ethyl)benzamide (29).** 4-(2-Azidoethyl)-*N*-(benzo[d]thiazol-2-yl)benzamide (**25**) (38.8 mg, 0.12 mmol) and *N*-(6-methoxybenzo[d]thiazol-2-yl)-2-(4-(prop-2-yn-1-yloxy)phenyl)-acetamide (**22**) (42.3 mg, 0.12 mmol) were used. Yield 68.9 mg, 85%. Beige solid, mp: 222 – 223 ºC (decomp.). ^{1}H-NMR (300 MHz, DMSO-d_6) δ (ppm): 12.41 (s, 2H), 8.17 (s, 1H), 8.08 – 7.91 (m, 3H), 7.78 (d, *J* = 8.1 Hz, 1H), 7.62 (d, *J* = 8.8 Hz, 1H), 7.53 (d, *J* = 2.6 Hz, 1H), 7.52 – 7.40 (m, 1H), 7.42 – 7.27 (m, 3H), 7.26 (d, *J* = 8.6 Hz, 2H), 7.06 – 6.90 (m, 3H), 5.09 (s, 2H), 4.68 (t, *J* = 7.2 Hz, 2H), 3.78 (s, 3H), 3.72 (s, 2H), 3.27 (t, *J* = 7.2 Hz, 2H). ^{13}C-NMR (75 MHz, DMSO-d_6) δ (ppm): 170.3, 157.1, 156.2, 156.0, 143.1, 142.6, 130.46, 129.1, 128.5, 127.1, 126.3, 124.6, 123.8, 121.8, 121.2, 120.4, 115.0, 114.8,

104.8, 61.2, 55.7, 50.0, 41.0, 35.5. ESI calcd for $C_{35}H_{30}N_7O_4S_2$ [M + H]+ 676.1795; found 676.1793.

***N*-(Benzo[d]thiazol-2-yl)-4-(2-(4-((4-(2-(benzo[d]thiazol-2-ylamino)-2-oxoethyl) phenoxy)methyl)-1H-1,2,3-triazol-1-yl)ethyl)benzamide (30).** *N*-(Benzo[d]thiazol-2-yl)-2-(4-(prop-2-yn-1-yloxy)phenyl)acetamide (**20**) (38.7 mg, 0.12 mmol) and 4-(2-azidoethyl)-*N*-(benzo[d]thiazol-2-yl)benzamide (**25**) (39.2 mg, 0.12 mmol) were used. Yield 26.4 mg, 34%. Beige solid, mp: 239 - 240 ºC ^{1}H-NMR (300 MHz, DMSO-d_6) δ (ppm): 12.82 (bs, 1H), 12.55 (s, 1H), 8.18 (s, 1H), 8.08 – 7.89 (m, 4H), 7.76 (dd, *J* = 13.2, 8.1 Hz, 2H), 7.61 – 7.21 (m, 8H), 6.99 (d, *J* = 8.7 Hz, 2H), 5.10 (s, 2H), 4.69 (t, *J* = 7.2 Hz, 2H), 3.76 (s, 2H), 3.34 (m, 2H). ^{13}C-NMR (75 MHz, DMSO-d_6) δ (ppm): 170.5, 157.9, 157.0, 148.5, 143.0, 142.6, 132.5, 131.4, 130.4, 129.0, 128.5, 126.9, 126.2, 126.1, 124.5, 123.7, 123.5, 121.7, 120.5, 114.7, 111.9, 61.1, 49.9, 35.4. ESI calcd. for $C_{34}H_{28}N_7O_3S_2$ [M + H]+ 646,1690; found 646.1683.

Ethyl 2-(2-(4-((1-(4-(2-(benzo[d]thiazol-2-ylamino)-2-oxoethyl)benzyl)-1H-1,2,3-triazol-4-yl)methoxy)phenyl)acetamido)-4-methylthiazole-5-carboxylate (31). 2-(4-(Azidomethyl)phenyl)-*N*-(benzo[d]thiazol-2-yl)acetamide (**11**) (29.8 mg, 0.09 mmol) and ethyl 4-methyl-2-(2-(4-(prop-2-yn-1-yloxy)phenyl)acetamido)thiazole-5-carboxylate (**23**) (33.0 mg, 0.09 mmol) were used. Yield 45.4 mg, 72%. White solid, mp: 195 - 196 ºC. ^{1}H-NMR (300 MHz, DMSO-d_6) δ (ppm): 12.66 (s, 1H), 12.59 (s, 1H), 8.26 (s, 1H), 7.98-7.93 (m, 1H), 7.74 (d, *J*= 8.0 Hz, 1H), 7.43 (t, *J*= 7.6 Hz, 1H), 7.35 (d, *J*= 7.6 Hz, 2H), 7.31-7.26 (m, 3H), 7.23 (d, *J*= 8.2 Hz, 2H), 6.98 (d, *J*= 8.5 Hz, 2H), 5.58 (s, 2H), 5.09 (s, 2H), 4.22 (q, *J*= 7.1 Hz, 2H), 3.83 (s, 2H), 3.71 (s, 2H), 2.54 (3H, s), 1.26 (t, *J*= 7.1 Hz, 3H). ^{13}C-NMR (75 MHz, DMSO-d_6) δ (ppm): 171.3, 171.0, 163.1, 160.5, 158.8, 158.0, 157.1, 149.5, 144.0, 135.63, 135.62, 132.4, 131.3, 130.7, 129.1, 127.7, 127.1, 125.6, 124.5, 122.7, 121.5, 115.7, 115.0, 62.0, 61.5, 53.5, 42.4, 41.8, 18.0, 15.2. ESI calcd. for $C_{34}H_{32}N_7O_5S_2$ [M + H]+ 682,1901; found 682.1897.

(*Z*)-*N*-(benzo[d]thiazol-2-yl)-4-((4-((4-((2-oxoindolin-3-ylidene)amino)phen-oxy)methyl)-1H-1,2,3-triazol-1-yl)methyl)benzamide (32). 4-(Azidomethyl)-*N*-(benzo[d]thiazol-2-yl)benzamide(**4**) (92.8 mg, 0.3 mmol) and (Z)-3-((4-(prop-2-

yn-1-yloxy)phenyl)imino)indolin-2-one (**13**) (82.9 mg, 0.3 mmol) were used. Yield 72.0 mg, 41 %. Red solid, m.p. 274 – 276 ºC, decompose. ^{1}H NMR (300 MHz, DMSO-d_6) δ >12.5 (s, 1H), 10.95 (s, 1H), 8.40 (d, *J* = 6.9 Hz, 1H), 8.15 (d, *J* = 7.6 Hz, 2H), 8.03 (d, *J* = 7.6 Hz, 1H), 7.89 – 7.68 (m, 1H), 7.48 (m, 3H), 7.35 (m, 2H), 7.15 (d, *J* = 8.0 Hz, 2H), 6.99 (d, *J* = 8.2 Hz, 2H), 6.89 (d, *J* = 7.8 Hz, 1H), 6.73 (d, *J* = 7.5 Hz, 1H), 6.61 (t, *J* = 7.5 Hz, 1H), 5.77 (s, 2H), 5.20 (s, 2H). ^{13}C NMR (75 MHz, DMSO) δ ^{13}C NMR (75 MHz, DMSO) δ 166.0, 164.0, 159.4, 156.6, 156.2, 155.1, 152.2, 147.2, 145.4, 143.9, 143.5, 141.8, 141.1, 134.6, 132.1, 129.2, 128.3, 126.6, 125.4, 125.4, 124.1, 122.9, 122.7, 122.5, 122.5, 122.1, 120.6, 119.7, 116.0, 114.6, 111.8, 110.9, 61.7, 52.6. ESI calcd. for $C_{32}H_{24}N_7O_3S$ [M + H]+ 586,1656; found 586.1655.

N-(benzo[d]thiazol-2-yl)-4-((1-(4-(benzo[d]thiazol-2-ylcarbamoyl)phenethyl)-1H-1,2,3-triazol-4-yl)methoxy)benzamide **(33).** 4-(2-Azidoethyl)-*N*-(benzo[d]thiazol-2-yl)benzamide (**27**) (97.0 mg, 0.3 mmol) and *N*-(Benzo[d]thiazol-2-yl)-4-(prop-2-yn-1-yloxy)benzamide (**7**) (92.5 mg, 0.3 mmol) were used. Yield 108.0 mg, 57%. White solid, m.p. 295-297 ºC. ^{1}H NMR (300 MHz, DMSO-d_6) δ >12.5 (bs, 2H), 8.24 (s, 1H), 8.16 (d, *J* = 8.2 Hz, 2H), 8.06 (d, *J* = 7.6 Hz, 2H), 7.98 (dd, *J* = 12.4, 5.6 Hz, 2H), 7.77 (m, 2H), 7.46 (t, *J* = 7.2 Hz, 2H), 7.39 (d, *J* = 7.6 Hz, 2H), 7.33 (t, *J* = 7.5 Hz, 2H), 7.19 (d, *J* = 8.2 Hz, 2H), 5.26 (s, 2H), 4.72 (t, *J* = 7.2 Hz, 2H), 3.32 (m, 2H). ^{13}C NMR (75 MHz, DMSO) δ 166.1, 165.5, 162.7, 159.3, 148.7, 143.3, 142.5, 131.9, 130.8, 130.6, 129.3, 128.8, 126.5, 125.1, 124.6, 124.6, 124.0, 123.9, 122.1, 120.6, 115.0, 61.7, 50.3, 36.1. ESI calcd. for $C_{33}H_{25}N_7NaO_3S_2$ $[M + Na]^+$ 654,1353; found 654.1349.

Biological studies

***In situ* click chemistry.** In a typical reaction, azide (1.66 µL of 3 mM DMSO solution) and alkyne (1.66 µL of 3 mM DMSO solution) were mixed with and without human recombinant BACE1 in 50 µL of 50 mM sodium acetate buffer pH 4.5, to afford final concentrations of 100 µM of azide and alkyne and 0.5 µM of BACE1. Reaction mixtures were vortexed and then incubated at room temperature for 24 h. Each sample and its control were analyzed by HPLC/MS instrument with SIM mode. Gradient MeCN/H_2O (0.1% formic acid in 10/90, v/v over 15 min at flow rate 0.5 mL min^{-1}.The identities of triazole products were

confirmed by molecular weight. Control experiments in the absence of BACE1 were set up every time the reactions were performed. Experiment with the presence of BSA (1 mg mL^{-1}) instead of the BACE1 enzyme was performed for azide 4 and alkyne 7.

LRRK2 inhibition enzymatic assay. The LRRK2 inhibitors were evaluated by the ThermoFisher company under the following Adapta Assay. The assay itself can be divided into two phases: a kinase reaction phase, and an ADP detection phase. In the kinase reaction phase, all components required for the kinase reaction are added to the well, and the reaction is allowed to incubate for 60 minutes. After the reaction, a detection solution consisting of a europium labeled anti-ADP antibody, an Alexa Fluor™ 647 labeled ADP tracer, and EDTA (to stop the kinase reaction) is added to the assay well. ADP formed by the kinase reaction (in the absence of an inhibitor) will displace the Alexa Fluor 647 labeled ADP tracer from the antibody, resulting in a decrease in the TR-FRET signal. In the presence of an inhibitor, the amount of ADP formed by the kinase reaction is reduced, and the resulting intact antibodytracer interaction results in a high TR-FRET signal. ADP formation is determined by calculating the emission ratio from the assay well.

The 2X LRRK2 / ERM (LRRKtide) mixture is prepared in 50 mM Tris pH 8.5, 0.01% Brij-35, 10 mM $MgCl_2$, 1 mM EGTA, 0.02% NaN_3. The final 10 µL Kinase Reaction consists of 3.75 - 70 ng LRRK2 and 200 µM ERM (LRRKtide) in 25 mM Tris / 7.5 mM HEPES pH 8.2, 0.005% Brij-35,5 mM $MgCl_2$, 0.5 mM EGTA, 0.01% NaN_3. After the 1 hour Kinase Reaction incubation, 5 µL of Detection Mix is added.

CK1δ inhibition enzymatic assay. The “Kinase-Glo” Kit from Promega was used to screen compounds for activity against CK1δ Kinase-Glo assays were performed in assay buffer using black 96-well plates. In a typical assay, 10 µl of test compound (dissolved in DMSO at 1 mM concentration and diluted in advance in assay buffer to the desired concentration) 10 µl (16 ng) of enzyme were added to each well followed by 20 µl of assay buffer containing 0,1 % casein as substrate and 4 µM ATP. The final DMSO concentration in the reaction mixture did not exceed 1%. After 60-min incubation at 30°C the enzymatic reaction was stopped

with 40 µl of Kinase-Glo reagent. Glow-type luminescence was recorded after 10 min using a FLUOstar Optima (BMG Labtechnologies GmbH, Offenburg, Germany) multimode reader. The activity is proportional to the difference of the total and consumed ATP. The inhibitory activities were calculated on the basis of maximal activities measured in the absence of inhibitor. The IC_{50} was defined as the concentration of each compound that reduces a 50% the enzymatic activity with respect to that without inhibitors.

GSK3β inhibition enzymatic assay. Human recombinant GSK3β and the pre-phosphorylated polypeptide substrate were purchased from Millipore (Millipore Ibérica SAU). Kinase-Glo Luminescent Kinase Assay was obtained from Promega (Promega Biotech Ibérica, SL). ATP and all other reagents were from Sigma Aldrich (St. Louis, MO). Assay buffer contained 50 mM HEPES (pH 7.5), 1 mM EDTA, 1 mM EGTA, and 15 mM magnesium acetate.

Kinase-Glo assays were performed in assay buffer using black 96-well plates. In a typical assay, 10 µL (10 µM) of test compound (dissolved in DMSO) at 1 mM concentration and diluted in advance in assay buffer to the desired concentration) and 10 µL (20 ng) of enzyme were added to each well followed by 20 µL of assay buffer containing 25 µM substrate and 1 µM ATP. The final DMSO concentration in the reaction mixture did not exceed 1%. After 30 min incubation at 30 °C, the enzymatic reaction was stopped with 40 µL of Kinase-Glo reagent. Glow-type luminescence was recorded after 10 min using a FLUOstar Optima (BMG Labtechnologies GmbH, Offenburg, Germany) multimode reader. The activity is proportional to the difference of the total and consumed ATP. The inhibitory activities were calculated based on maximal activities measured in the absence of inhibitor. The IC_{50} was defined as the concentration of each compound that reduces a 50 % the enzymatic activity with respect to that without inhibitors.

BACE1 enzymatic assay. BACE1 in vitro assays were carried out using FRET. An APP-based peptide substrate (rhodamine-EVNLDAEFK-quencher, *K*m of 20 µM) carrying the Swedish mutation and containing a rhodamine as a fluorescence donor and a quencher acceptor at each end was used. The intact substrate is weakly fluorescent and becomes highly fluorescent upon enzymatic cleavage. The assays were conducted in 50 mM sodium acetate buffer, pH 4.5, in a final

enzyme concentration (1 U/mL). Inhibitor screening at 10 µM. The mixture was incubated for 60 min at 25 °C under dark conditions and then stopped with 2.5 M sodium acetate. Fluorescence was measured with a FLUOstar Optima (BMG Labtechnologies GmbH, Offenburg, Germany) microplate reader at 545 nm excitation and 585 nm emission.

Evaluation of BACE Inhibitors Kinetics. To evaluate the mechanism of BACE1 inhibition of the compounds, substrate-dependent kinetic experiments were performed. BACE activity were measured at five different concentrations substrate (1000 -62,5 µM) in the absence or presence of the select inhibitor at two different concentrations. The results are presented as double reciprocal Lineweaver−Burk plots (1/V vs 1/[S]) Figure S1.

Cell culture and treatments. SK-N-MC cells stably expressing the human isoform APP695 (SK-APP cell line D1; SKAPP-D1) were generated by transfection of SK-N-MC cells with a pCDNA3-APP expression vector, and kindly provided by Dr. MJ Bullido. These cells were cultured in DMEM (Thermo Fisher) medium supplemented with 10% FBS (Thermo Fisher), 1 mM glutamine (Sigma-Aldrich), and 100 unit/ml penicillin (Lonza) and 100 mg/ml streptomycin (Lonza). Cell cultures were maintained in a 5% CO_2, humidified atmosphere at 37 °C.
SH-SY5Y cells were obtained from the American Type Culture Collection and grown in DMEM media (Gibco), supplemented with 10% foetal bovine serum, and 1% penicillin/streptomycin (Invitrogen). The cells were grown at 37 °C in a humidified 5% CO_2 atmosphere. For the treatments, all the compounds were dissolved in dimethyl sulfoxide (DMSO, from Sigma) at an initial stock concentration of 10 mM. A 250X working solutions were obtained from the dilution of the stock solutions in DMEM media (DMSO control solutions were prepared similarly to the compounds). Okadaic acid (OA, Sigma-Aldrich) was dissolved in DMSO at an initial stock concentration of 60 µM and a 250X working solution was obtained from the dilution of the stock solution in DMEM.

All the compounds (250x solutions) were added directly into the wells at the indicated final concentrations for 1 hour before the treatment with OA at a final concentration of 30 nM. DMSO was added at the appropriate concentration to the

control-untreated cells. The cells were maintained in the presence of the OA and the different compounds for 24h.

Measurement of cell viability. Cell viability assays were carried out using a 3-(4,5-Dimethyl-2-thiazolyl)-2,5-diphenyl-2H-tetrazolium bromide (MTT) assay (Sigma-Aldrich) in 96-well cell culture plates. All cells were then incubated with a 0.5 mg/ml MTT solution (Sigma-Aldrich) at 37 °C for 4 h and then with cell lysis buffer (20% SDS, 50% N,N-dimethylformamide, pH 4.7) for 3 h. The absorbance values were determined at 590 nm. Cell viability was expressed as a percent of OD of control cells, the value of which was defined as 100%.

Cell viability in SH-SY5Y were carried out following the same procedure but seeding the cells in 24 well multiplates. Absorbance was measured using a Varioskan Flash multiplate reader (Thermo Scientific) at test 550 nm and reference 664 nm wave-lengths. Compound concentration for SK-APP line was 10 µM and for the SH-SY5Y was 1 and 5 µM.

Immunoassays. Levels of $A\beta_{40}$ and $A\beta_{42}$ were quantified by sandwich ELISAs (Wako, Osaka, Japan) from cellular lysates and conditioned media collected.

CNS penetration: in vitro PAMPA-BBB test. Prediction of the brain penetration was evaluated using a parallel artificial membrane permeability assay (PAMPA).[1] Nine quality control compounds (mentioned below) of know BBB permeability were included in each experiment to validate the analysis set.[2] Commercial drugs (3-5 mg of atenolol, caffeine, desipramine, enoxacine, ofloxacine, piroxicam, testosterone, 12 mg of promazine and 25 mg of verapamile, Sigma-Aldrich) were dissolved in 250 µL of DMSO (Sigma-Aldrich). 25 µL of these compound stock solutions were taken and 225 µL of DMSO and 4750 µL of PBS pH 7.4 buffer were added to reach 5% of DMSO concentration in the experiment and a final compound concentration in the range of 0.2 to 1.1 mM. Compounds to be determined their ability to pass the brain barrier (0.5-1 mg) were dissolved in 250 µL of DMSO and 4750 µL of PBS pH 7.4 was carefully added to afford a final compound concentration in the range of 0.2 to 1.1 mM. Solutions were filtered using filter PDVF membrane units (diameter 30 mm, pore size 0.45 µm, Symta). 10 µL of each solution were taken and diluted 10-fold with PBS/DMSO (95/5) for HPLC-MS analysis. The acceptor 96-well plate (Multiscreen®, catalog no.

MAMCS9610, Millipore) was filled with 180 µL of PBS/DMSO (95/5). The donor 96-well plate (Multiscreen® IP Sterile Plate PDVF membrane, pore size is 0.45 M, catalog no. MAIPS4510, Millipore) was coated with 4 µL 20 mg·mL-1 of porcine brain lipid (catalog no. 141101, Avanti Polar Lipids) in dodecane (Fluka) for 5 min and then 180 µL of each compound solution was added. Then the donor plate was put on the acceptor plate and kept for 2.5 h at room temperature. After incubation, samples were collected from the acceptor plate and diluted 10-fold with PBS/DMSO (95/5) for HPLC-MS analysis and compared to HPLC-MS of initial solutions. Absorbance peak of each compound was determined by MS detection and was used to calculate the concentration of compounds and experimental *Pe* (10^{-6} cm s^{-1}). Every sample was analysed in two independent runs. HPLC-MS was performed using a HPLC-MS equipment from Thermo Fisher coupled to a Finnigan TM LXQ TM detector. Each compound was analysed using a Sunfire column C18, 3.5 µm (50 mm x 4.6 mm) and acetonitrile and Milli-Q water (with 0.1% formic acid) as mobile phase. The standard gradient consisted of a 7 min run from 5% to 95% of acetonitrile at a flow rate of 0.5 mL min^{-1}. A good correlation between experimental-described values was obtained P_e (Exp) = 0.6639 (bibl.) + 0.5767 (R^2 = 0.9638). From the pattern previously established in the literature for BBB permeation prediction[2] and this equation, compounds were classified as CNS+ when they present a permeability higher than 3.20 x 10^{-6} cm s^{-1}.

Computational studies.

To appropriately select the correct crystal structure for BACE1 computational studies we created a virtual library of all the ligands (excluding peptide-like compounds and ions) co-crystallized with the BACE1 in the Protein data base (PDB) and prepared them with LigPrep at pH 7.0 ± 2.0 selecting only one conformer per compound. Then the ligands were compared with compound 8 using the ligand-based virtual screening LiSiCA software,[3] which uses the Tanimoto coefficient to search 3D similarities between a given set of compounds. This step gave us 5I3Y 3D-crystallografic structure,[4] which is the BACE1 target co-crystallized with a ligand that is very similar to our reference compound, so the

binding site is adapted to this compound. Performing docking simulations with this crystalline structure increases the probability of finding acceptable solutions for the compounds studied in this work.

The 5I3Y was prepared with the Maestro module Protein Preparation Wizard, the hydrogen bonds were optimized using PROPKA at pH 7 and the co-crystalized ligand was removed for further docking simulations. The grid box was centered at Asp32 and Asp228 catalytic residues, with a size large enough to include the sites involved in the binding site of the crystallized ligand. Molecular docking studies with the selected compounds was performed using the software Glide[5] and the Standard Precision (SP) scoring function, the top-10 poses per docking were collected and postprocessed by using the molecular mechanics generalized Born surface area (MM-GBSA) method implemented in Prime software,[6] The MM-GBSA method has been used to postprocess docking solutions because it has shown more accurate results than the classical docking score.[7, 8, 9] With MM-GBSA, the binding free energy between the BACE1 and the compounds studied is calculated by frozen the entire protein and then minimized the ligand as well as the residues within 5Å of each compound which allows receptor flexibility to adapt the residues involved in the binding site of each compound in each simulation. The best results according to the calculated free energy were selected for the analyses presented here.

The complexes between BACE1 and compounds **14**, **32**, and the fragment MBC-2137, which presents the best predicted binding free energy (MM-GBSA), were subjected to 525 ns of molecular dynamics simulations (MDs) using Desmond software[10] and the OPLS3e force field .[11] To prepare the systems, the complexes were embedded into a pre-equilibrated phosphatidyl oleoyl phosphatidylcholine (POPC) bilayer in a periodic boundary condition box with pre-equilibrated SPC water molecules. Then the systems were neutralized by adding Na^+ or Cl^- counter ions to balance the net charge of the systems and NaCl at a concentration of 0.15 M was added to simulate physiological conditions of the systems. Each system was relaxted using the default Desmond relaxation protocol and then equilibrated with a spring constant force of 1.0 $kcal \times mol^{-1} \times Å^{-2}$ applied to the BACE1 backbone atoms and the ligands for 25 ns using the NPT ensemble at constant pressure (1 atm), temperature (310 K), and number of atoms using the

isothermal-isobaric ensemble and the Nosé–Hoover method with a relaxation time of 1 ps applying the MTK algorithm,[12] with a timestep of 2 fs. Then the last frame was taken and a second non-restricted 500 ns MDs was performed using the same conditions previously described. Systems were analyzed using the Thermal MMGBSA script available in the Schrödinger script center, as well as the contact surface analyzer[13] and in house scripts.

^{1}H and ^{13}C NMR Spectra.

Compound 3

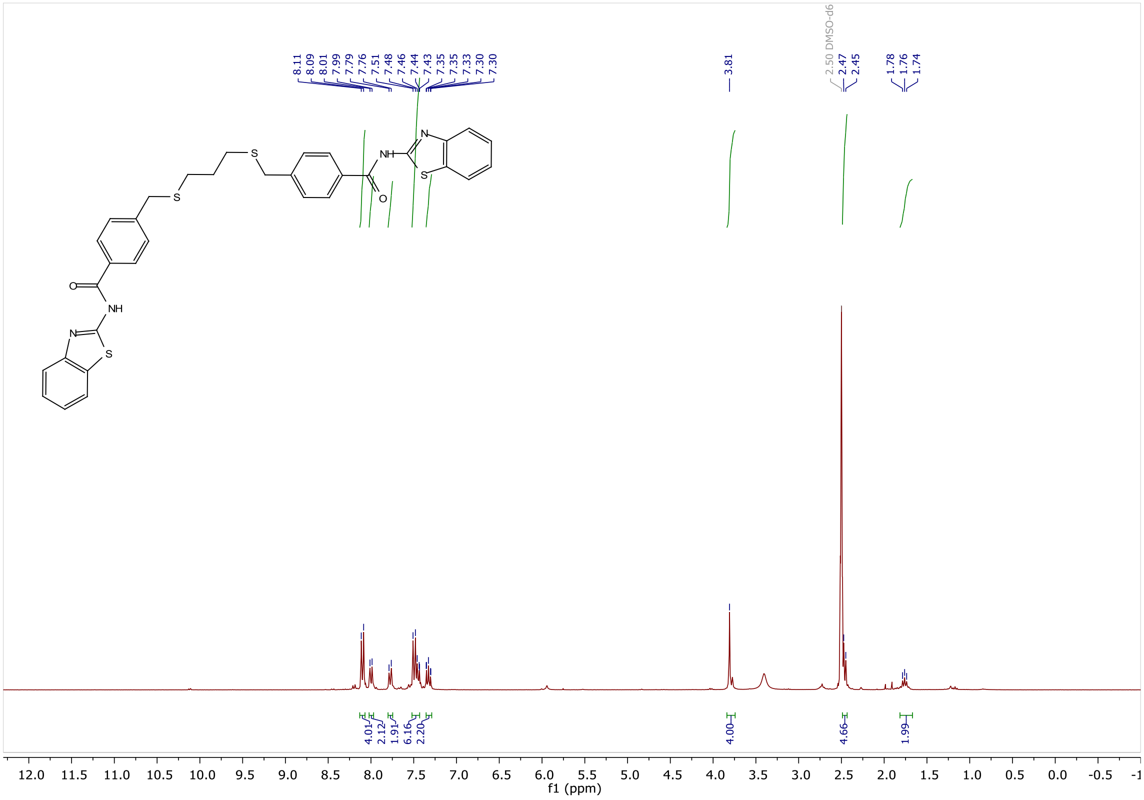

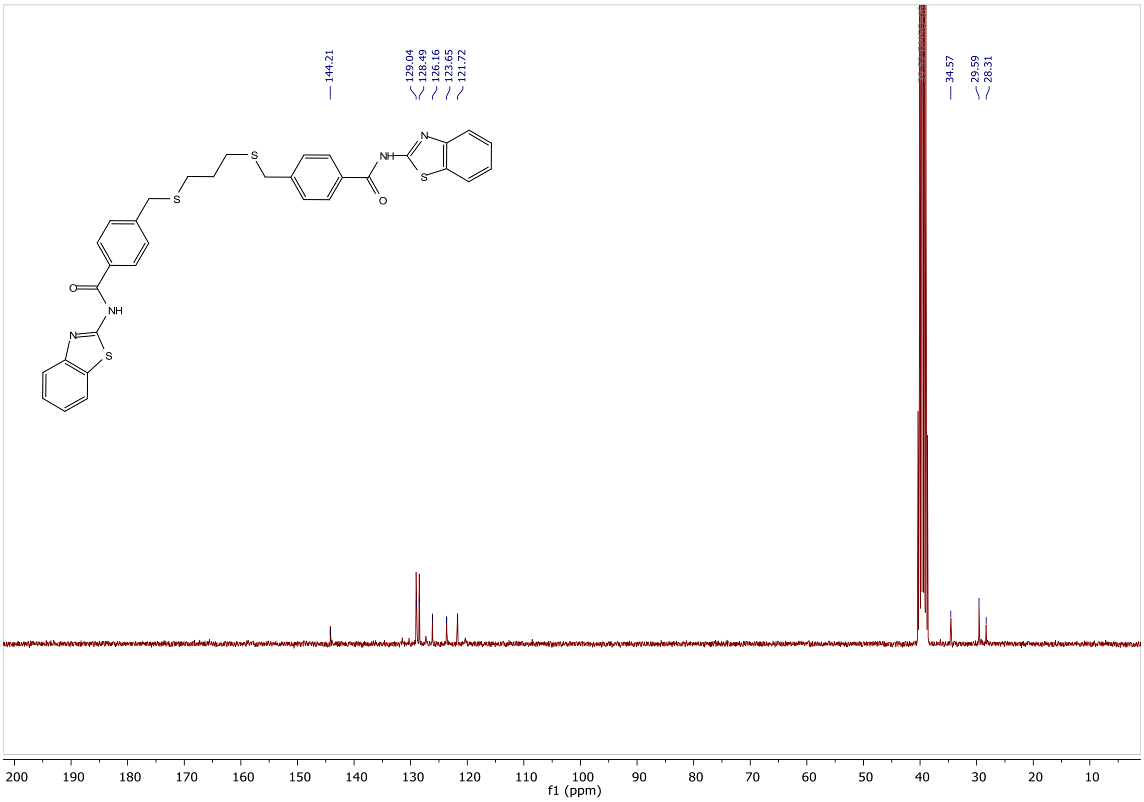

Compound 4

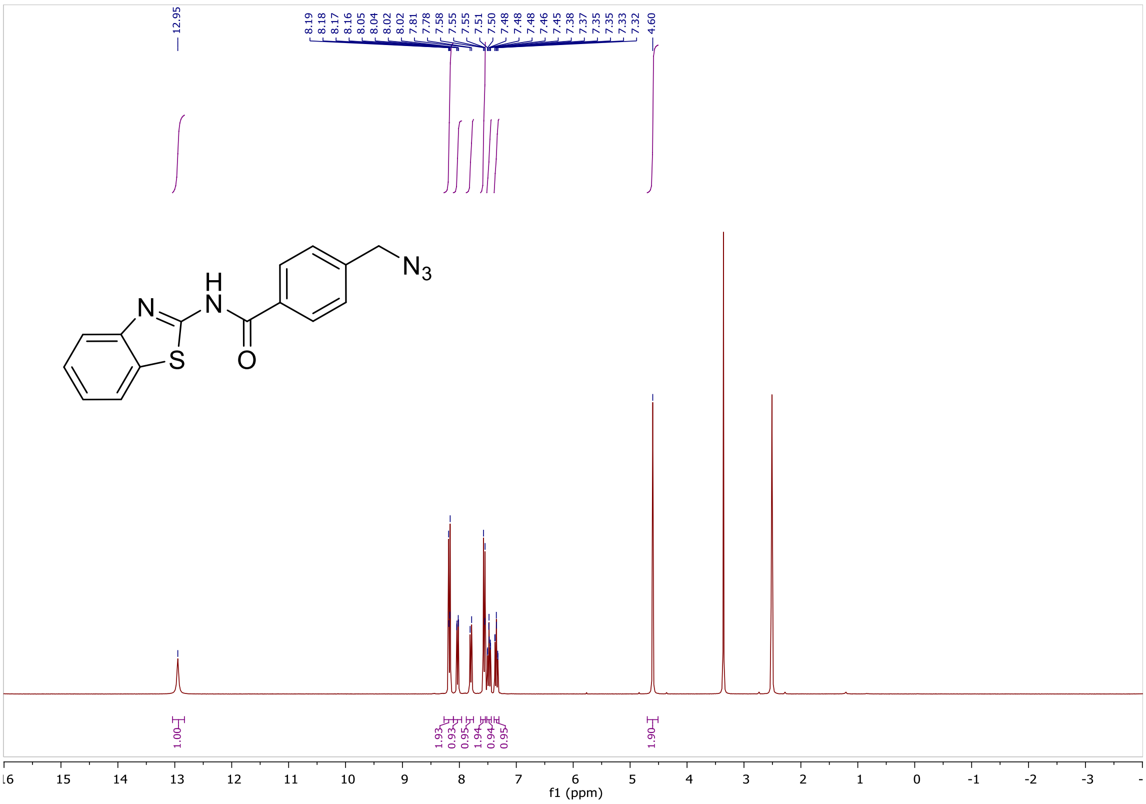

12.95
8.19 8.18 8.17 8.16 8.05 8.04 8.02 8.02 7.81 7.78 7.58 7.55 7.55 7.51 7.50 7.48 7.48 7.46 7.45 7.38 7.37 7.35 7.35 7.33 7.32
4.60
N
H
N
N_3
S
O
1.00
1.93 0.93 0.95 1.94 0.94 0.95
1.90
f1 (ppm)

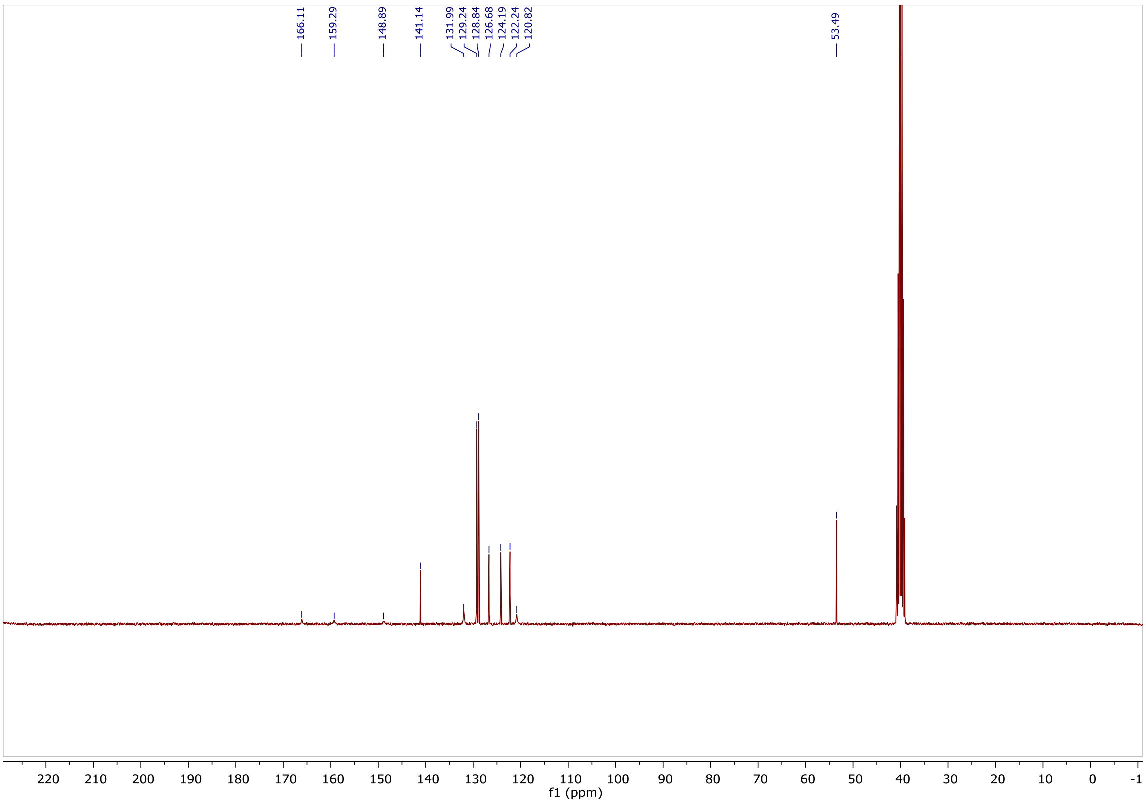

166.11
159.29
148.89
141.14
131.99 129.24 128.84 126.68 124.19 122.24 120.82
53.49
f1 (ppm)

Compound 7

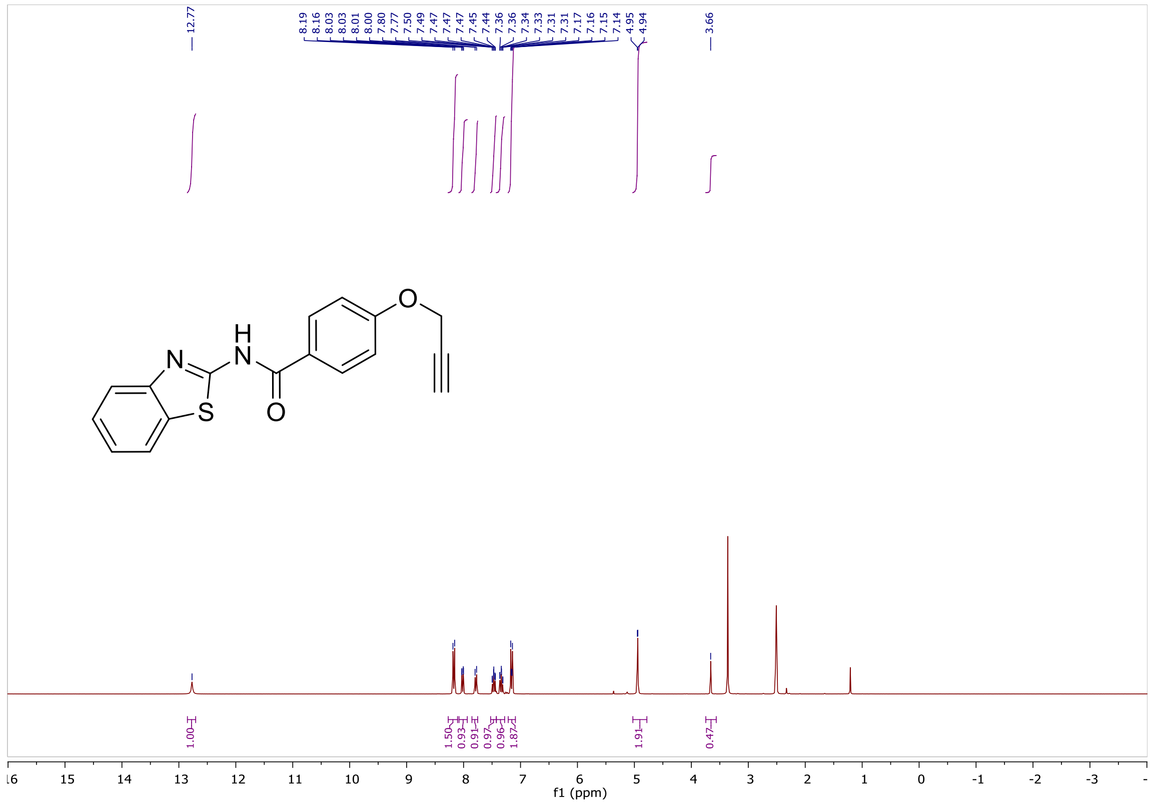
12.77
8.19 8.16 8.03 8.03 8.01 8.00 7.80 7.77 7.50 7.49 7.47 7.47 7.47 7.45 7.44 7.36 7.36 7.34 7.33 7.31 7.31 7.17 7.16 7.15 7.14 4.95 4.94
3.66
H
N
N
S
O
O
1.00
1.50
0.93
0.91
0.97
0.96
1.87
1.91
0.47
f1 (ppm)

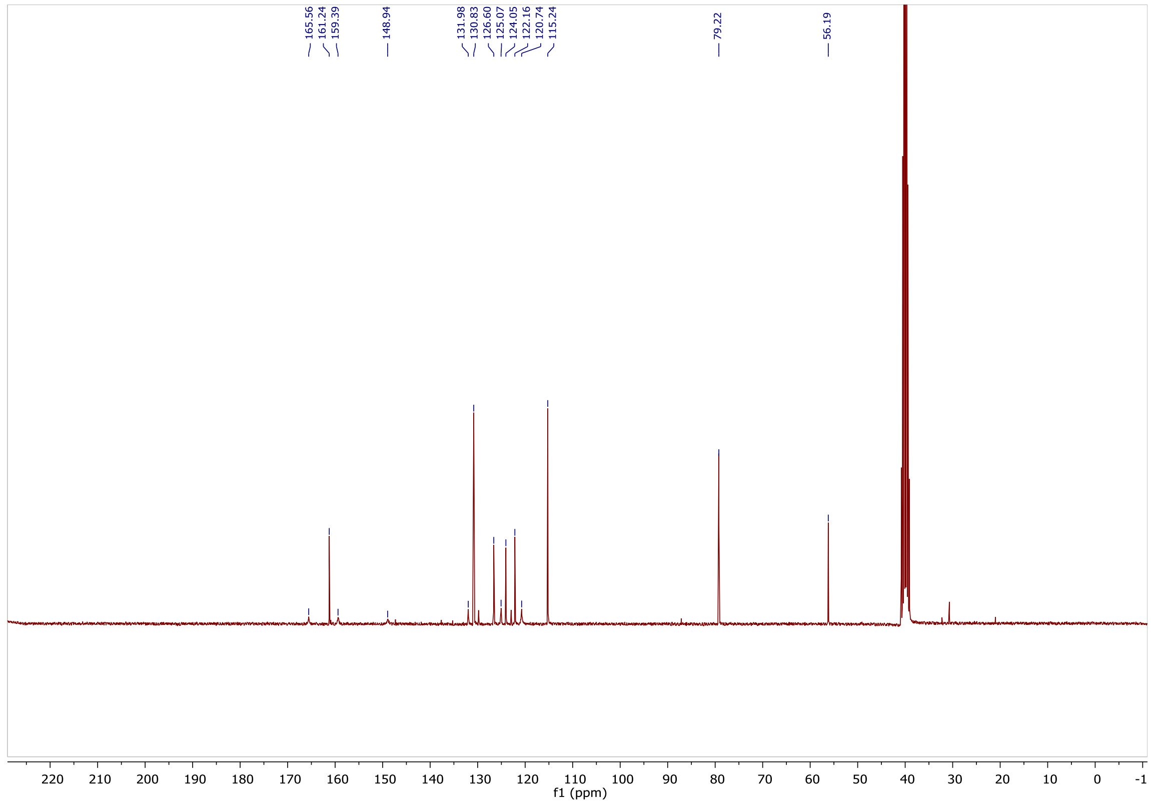
165.56 161.24 159.39
148.94
131.98 130.83 126.60 125.07 124.05 122.16 120.74 115.24
79.22
56.19
f1 (ppm)

Compound 8

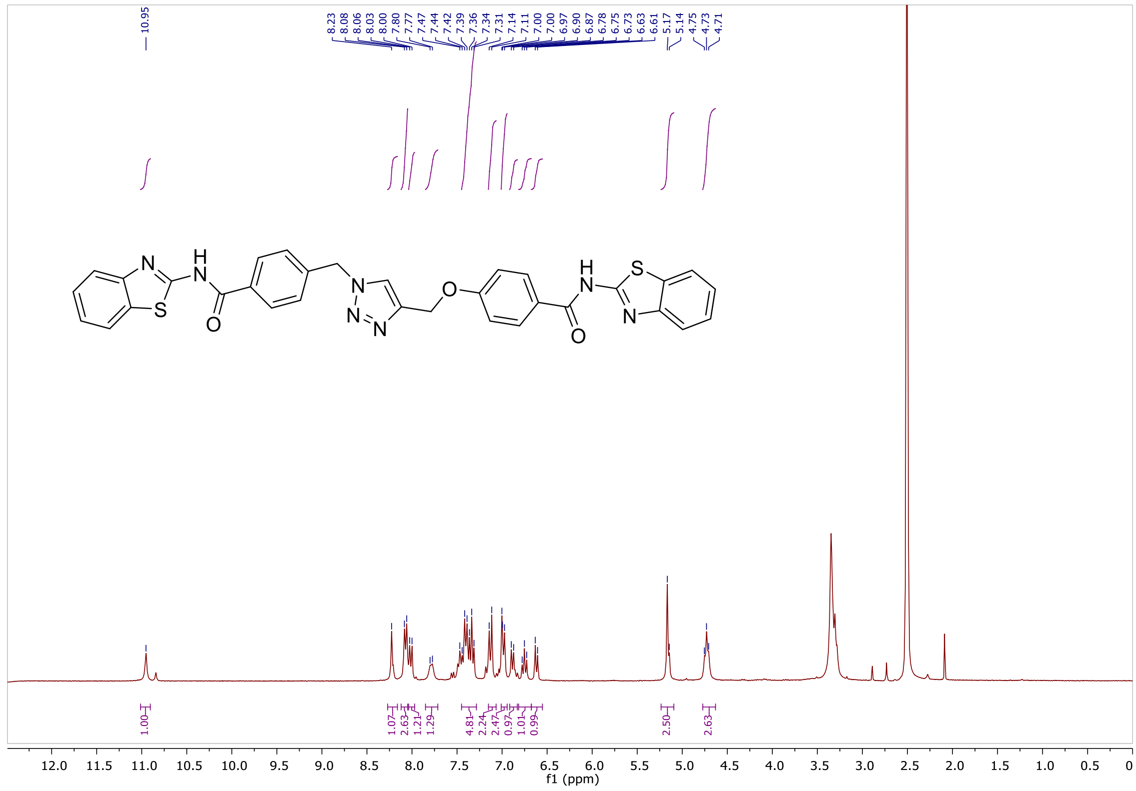

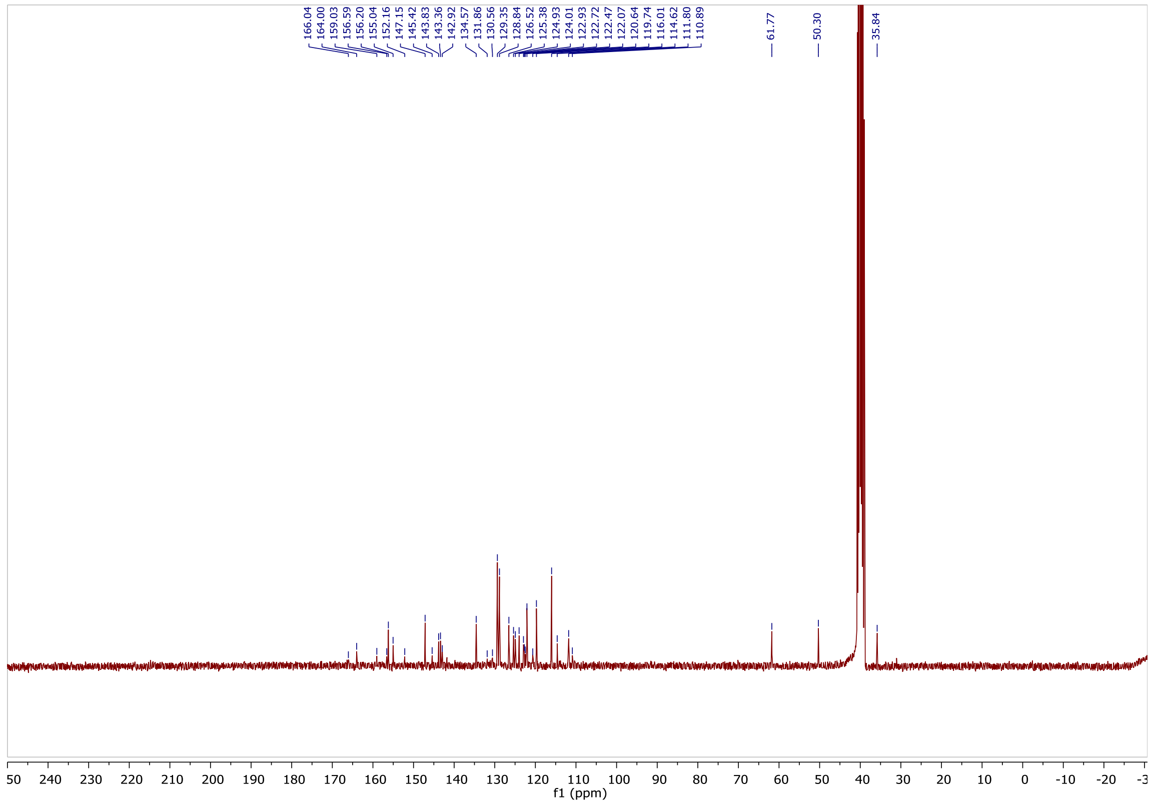

Compound 11

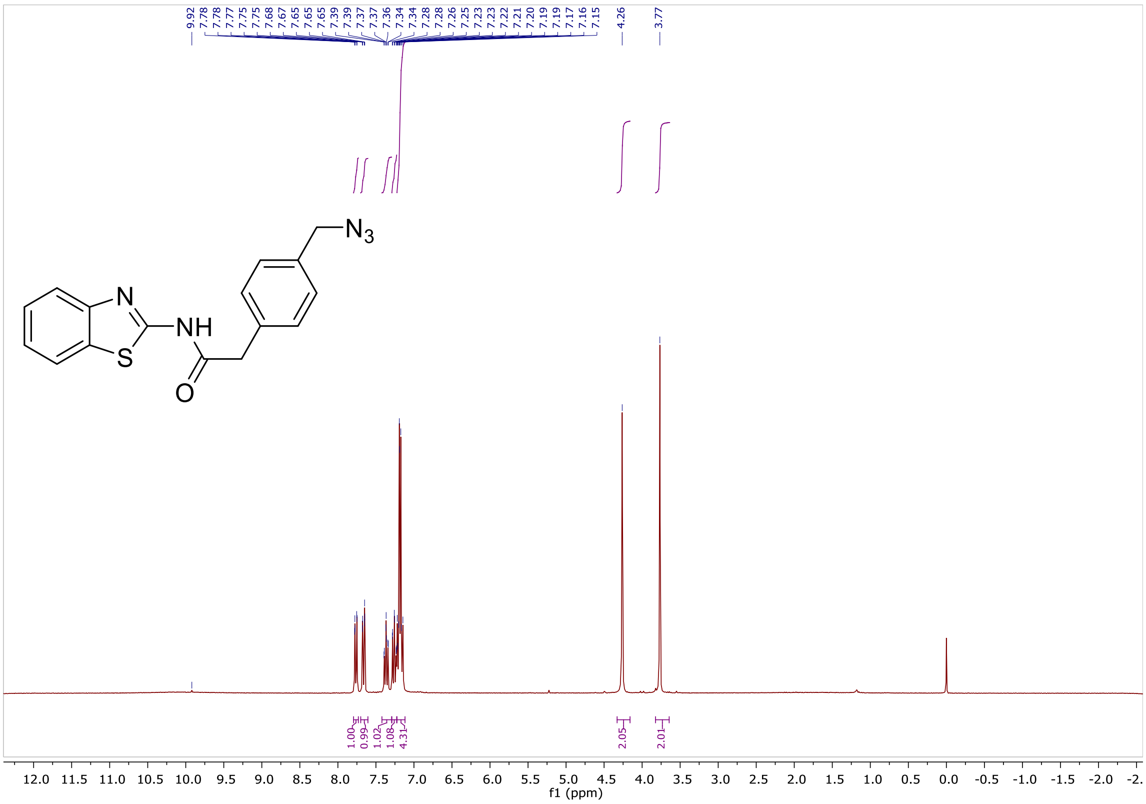

9.92
7.78
7.78
7.77
7.75
7.75
7.68
7.67
7.65
7.65
7.39
7.39
7.37
7.37
7.36
7.34
7.34
7.28
7.28
7.26
7.25
7.23
7.23
7.22
7.21
7.20
7.19
7.17
7.16
7.15
4.26
3.77
N
NH
S
O
N3
1.00
0.99
1.02
1.08
4.31
2.05
2.01
f1 (ppm)

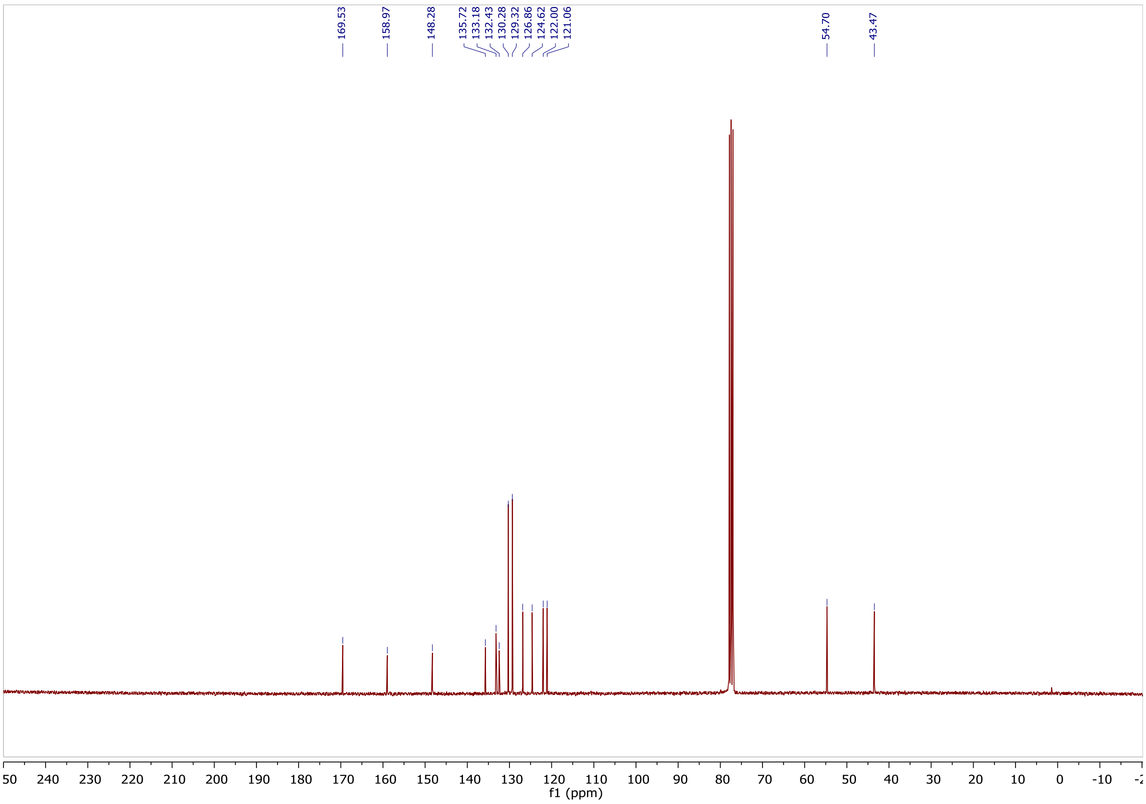

169.53
158.97
148.28
135.72
133.18
132.43
130.28
129.32
126.86
124.62
122.00
121.06
54.70
43.47
f1 (ppm)

Compound 12

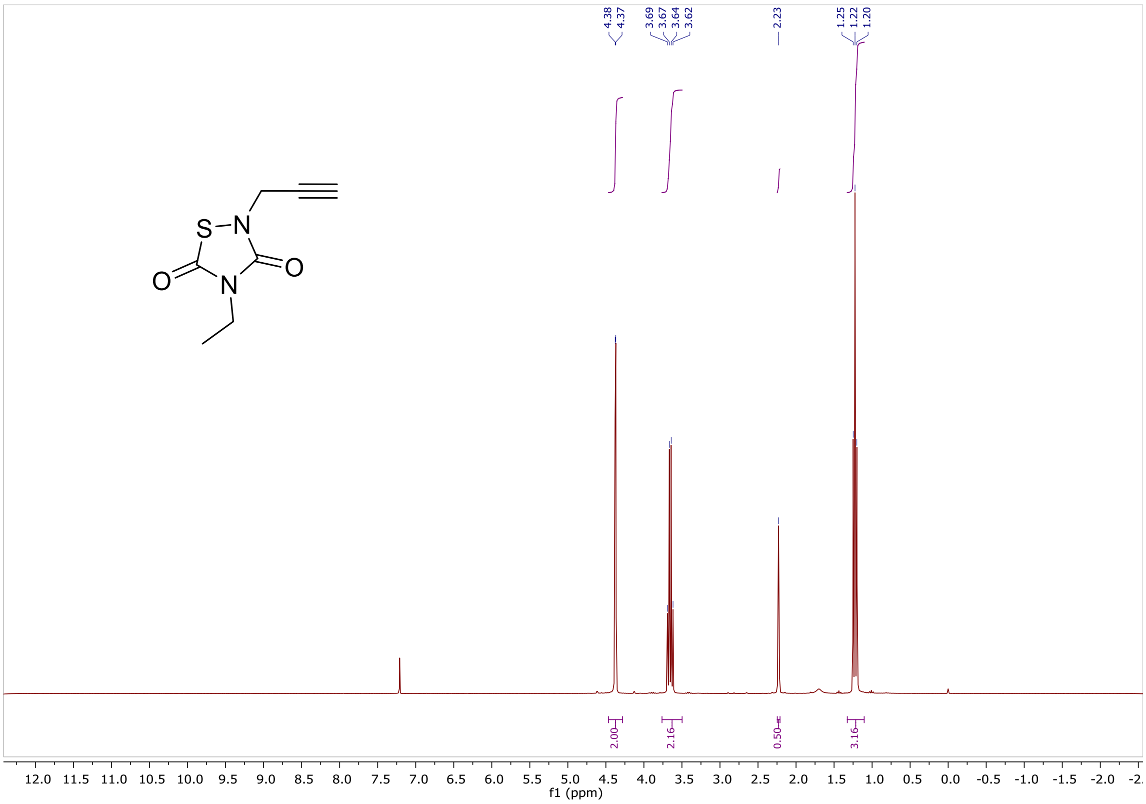
4.38
4.37
3.69
3.67
3.64
3.62
2.23
1.25
1.22
1.20
S
N
N
O
O
2.00
2.16
0.50
3.16
f1 (ppm)

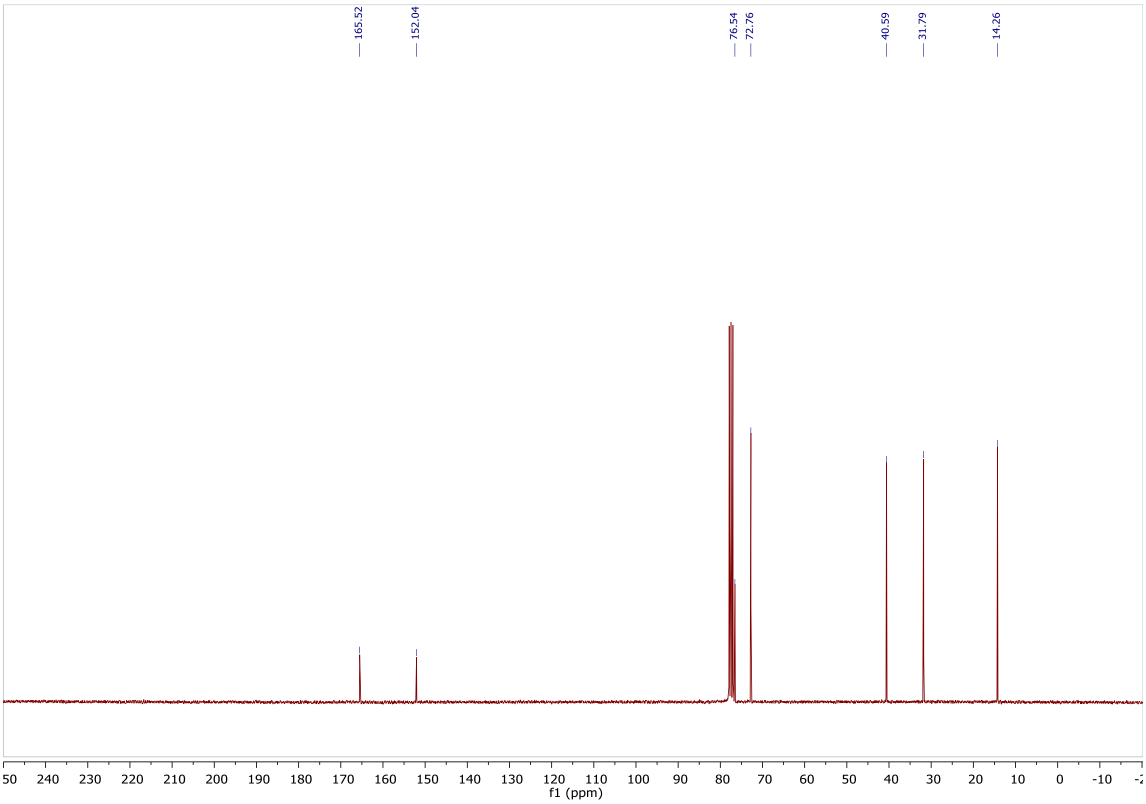
165.52
152.04
76.54
72.76
40.59
31.79
14.26
f1 (ppm)

Compound 13

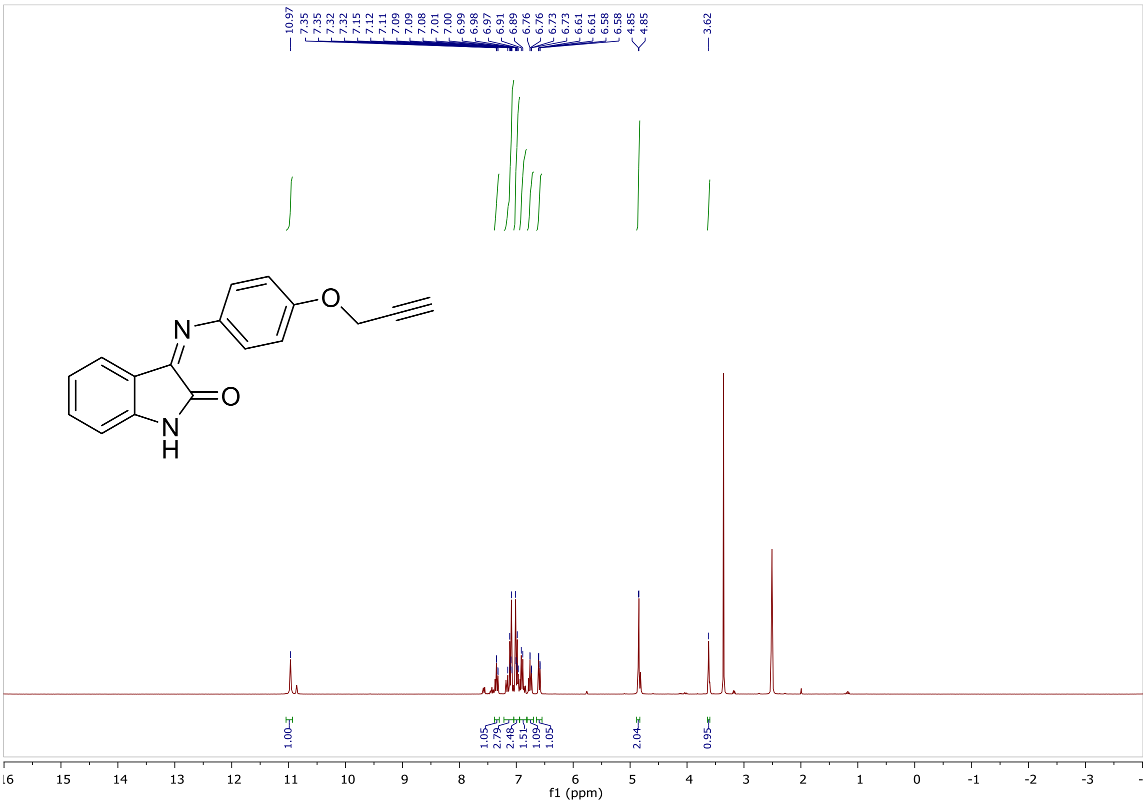

10.97
7.35
7.35
7.32
7.32
7.15
7.12
7.11
7.09
7.09
7.08
7.01
7.00
6.99
6.98
6.97
6.91
6.89
6.76
6.76
6.73
6.73
6.61
6.61
6.58
6.58
4.85
4.85
3.62
1.00
1.05
2.79
2.48
1.51
1.09
1.05
2.04
0.95
f1 (ppm)

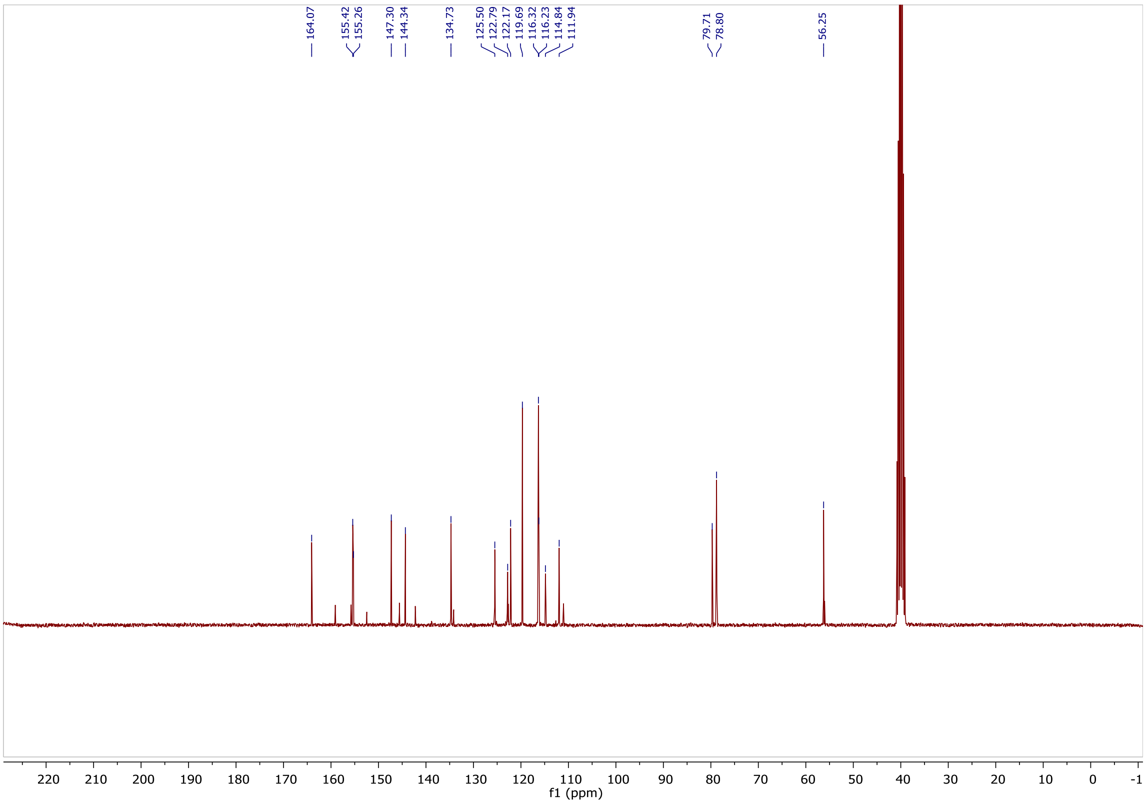

164.07
155.42
155.26
147.30
144.34
134.73
125.50
122.79
122.17
119.69
116.32
116.23
114.84
111.94
79.71
78.80
56.25
f1 (ppm)

Compound 14

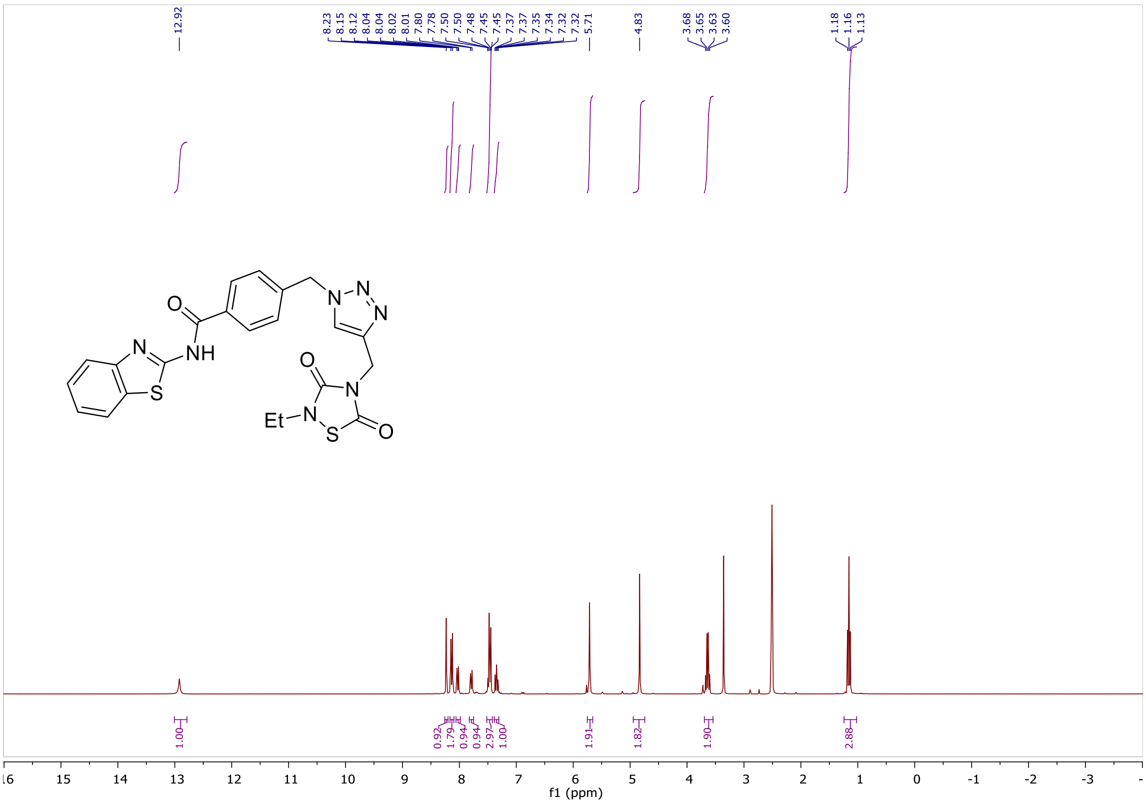

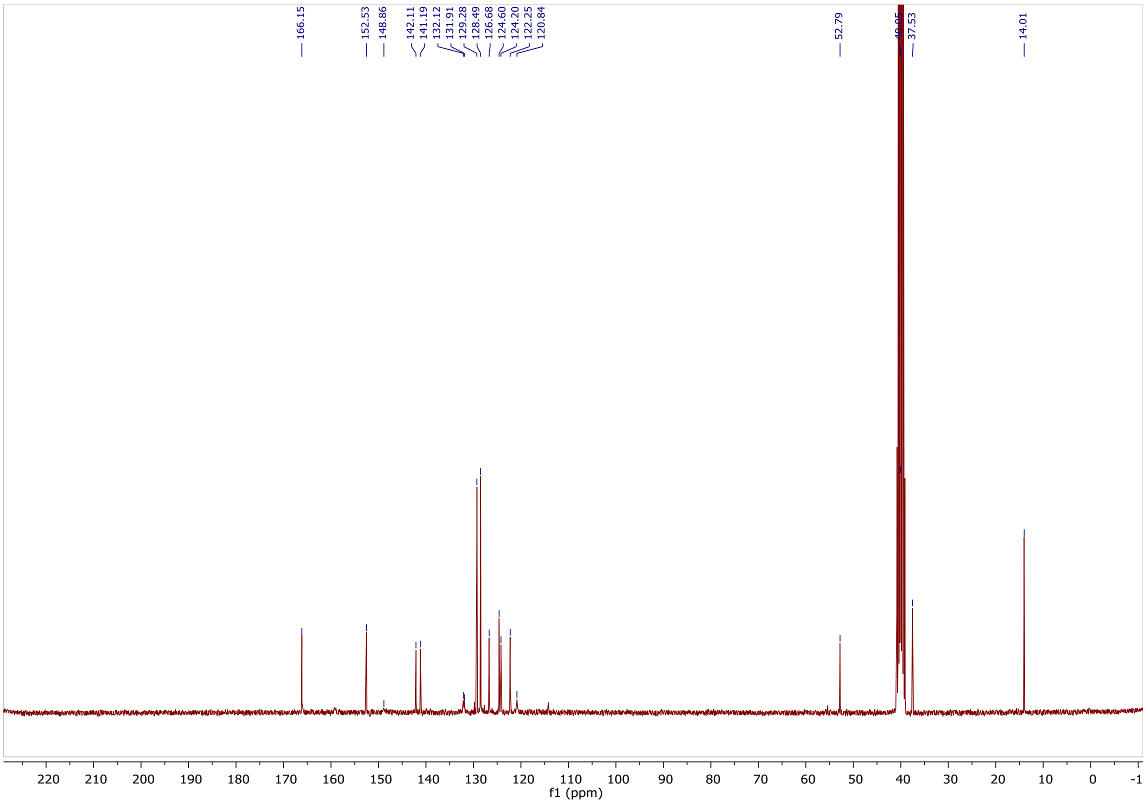

Compound 15

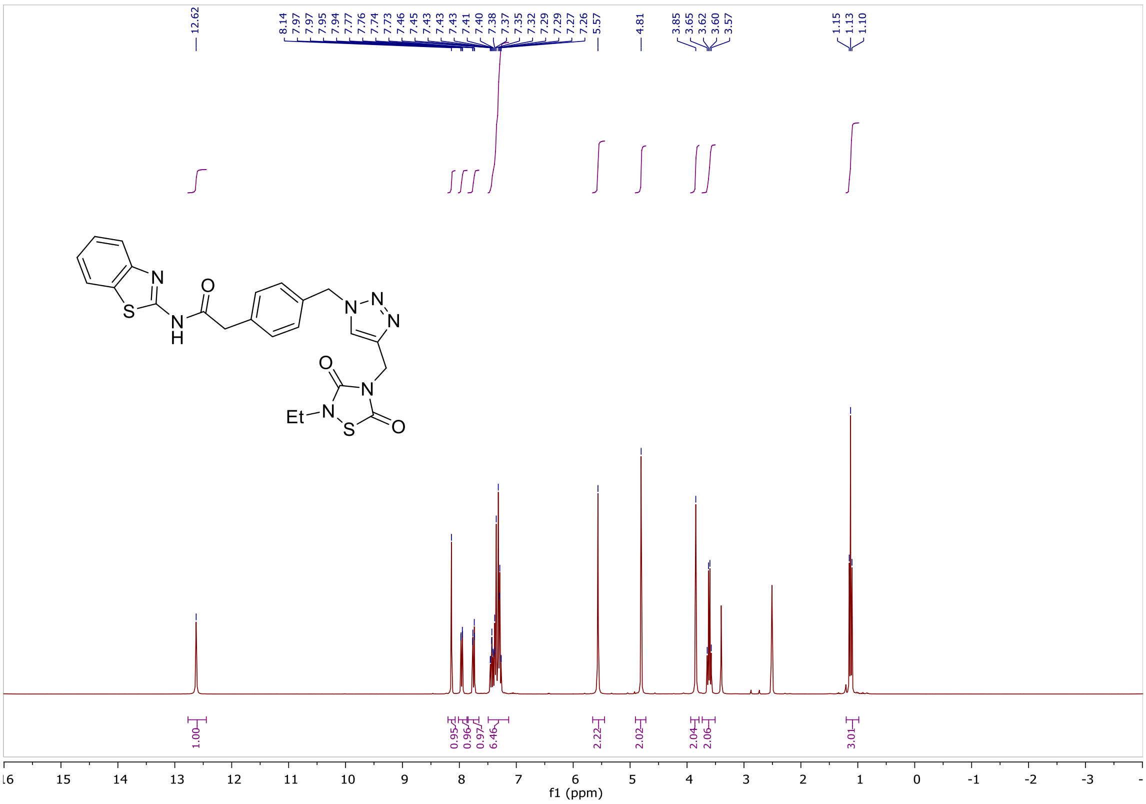

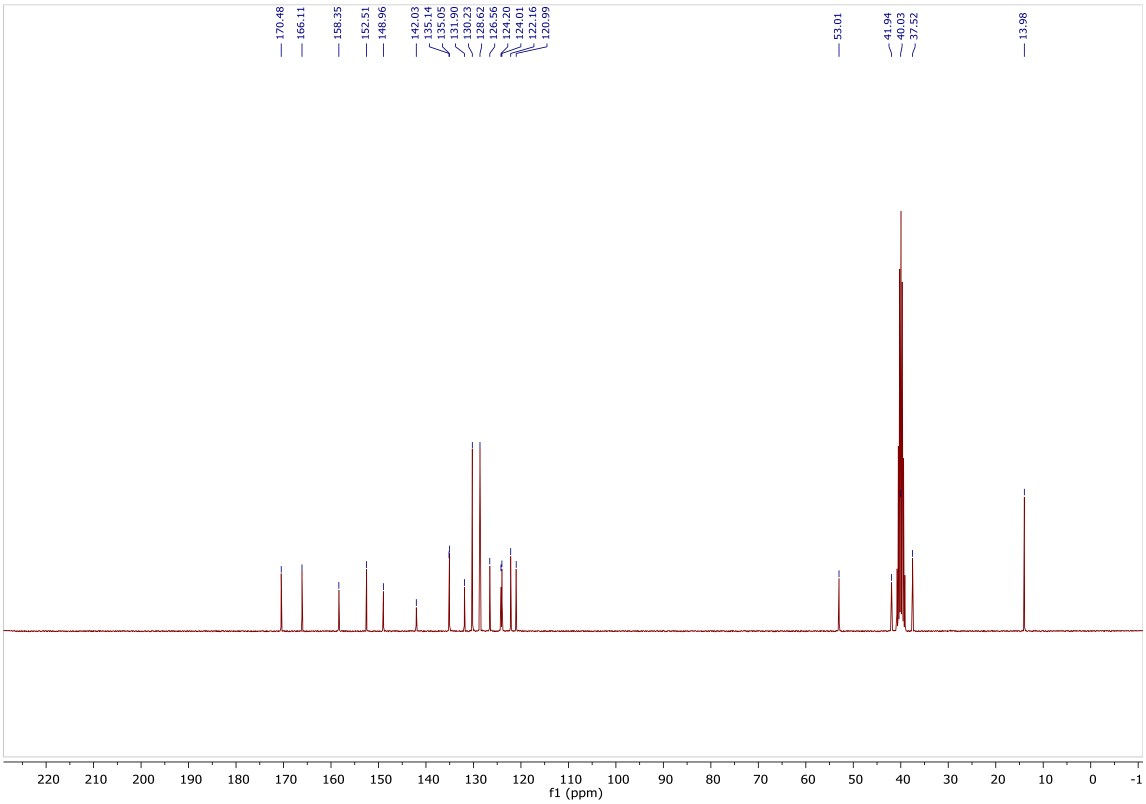

Compound 16

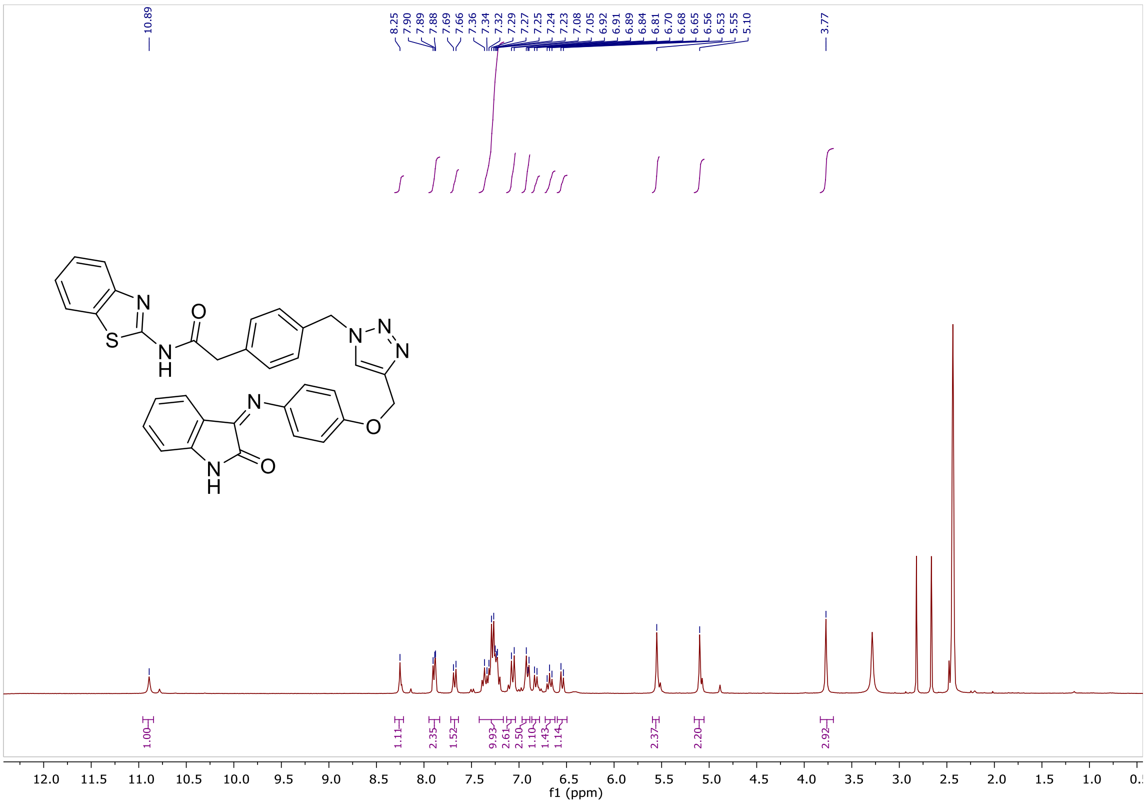

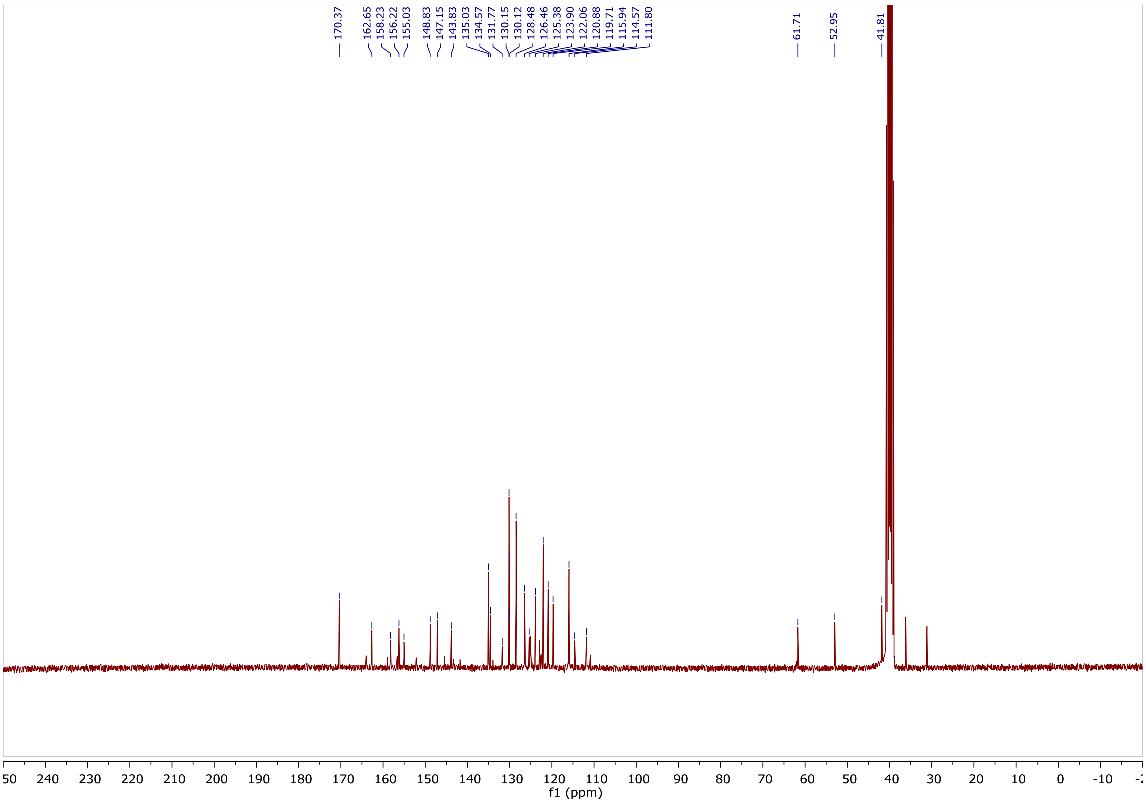

Compound 17

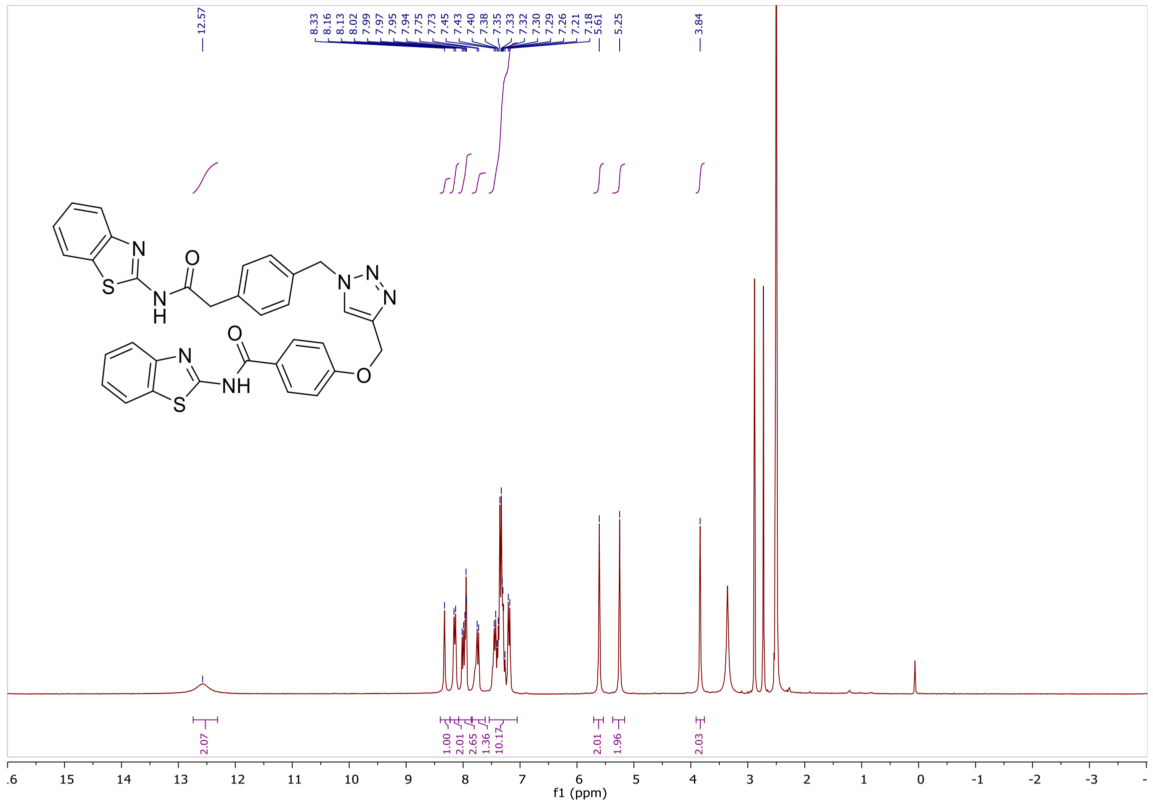

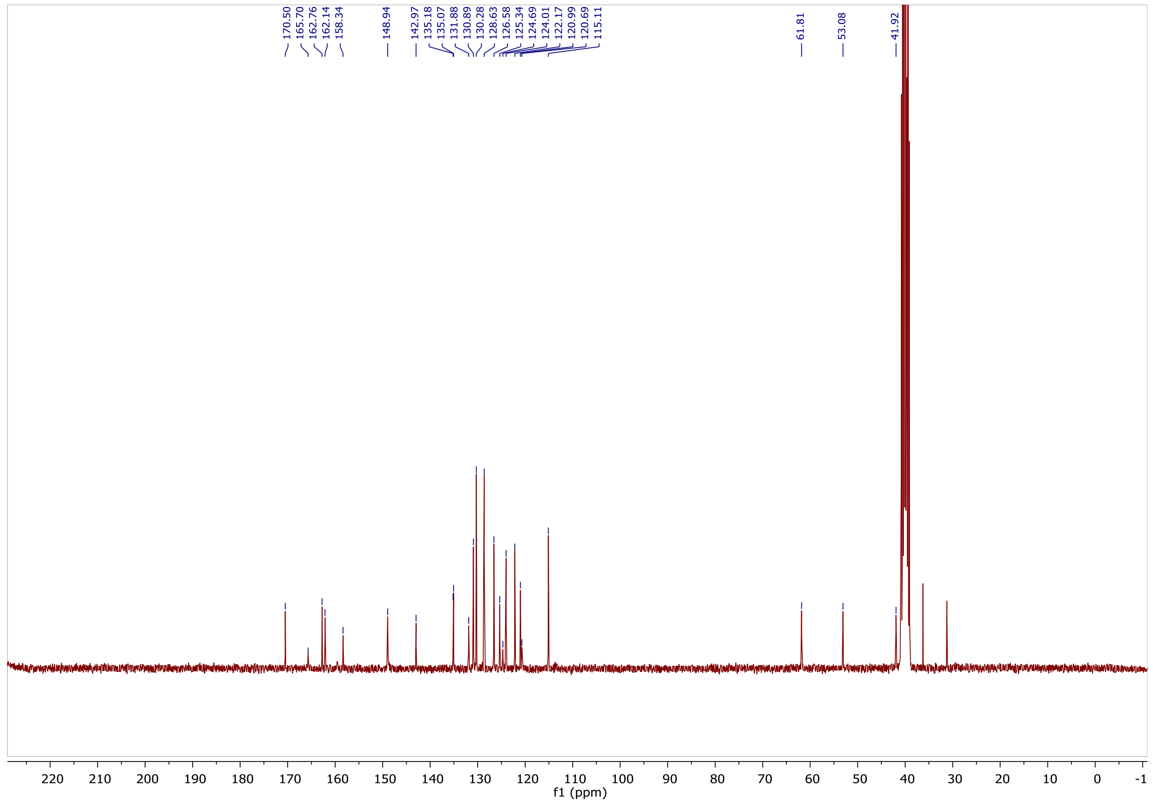

Compound 18

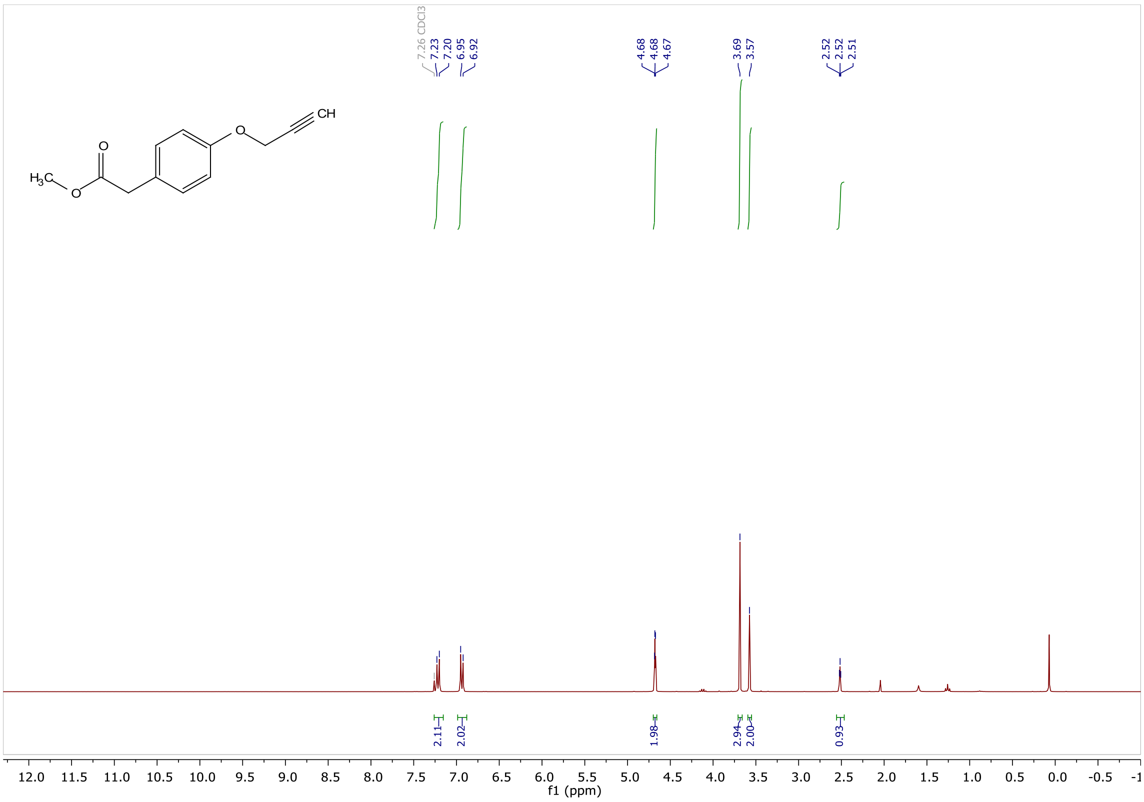

7.26 CDCl3
7.23
7.20
6.95
6.92
4.68
4.68
4.67
3.69
3.57
2.52
2.52
2.51
CH
O
O
H3C
O
2.11
2.02
1.98
2.94
2.00
0.93
12.0 11.5 11.0 10.5 10.0 9.5 9.0 8.5 8.0 7.5 7.0 6.5 6.0 5.5 5.0 4.5 4.0 3.5 3.0 2.5 2.0 1.5 1.0 0.5 0.0 -0.5 -1
f1 (ppm)

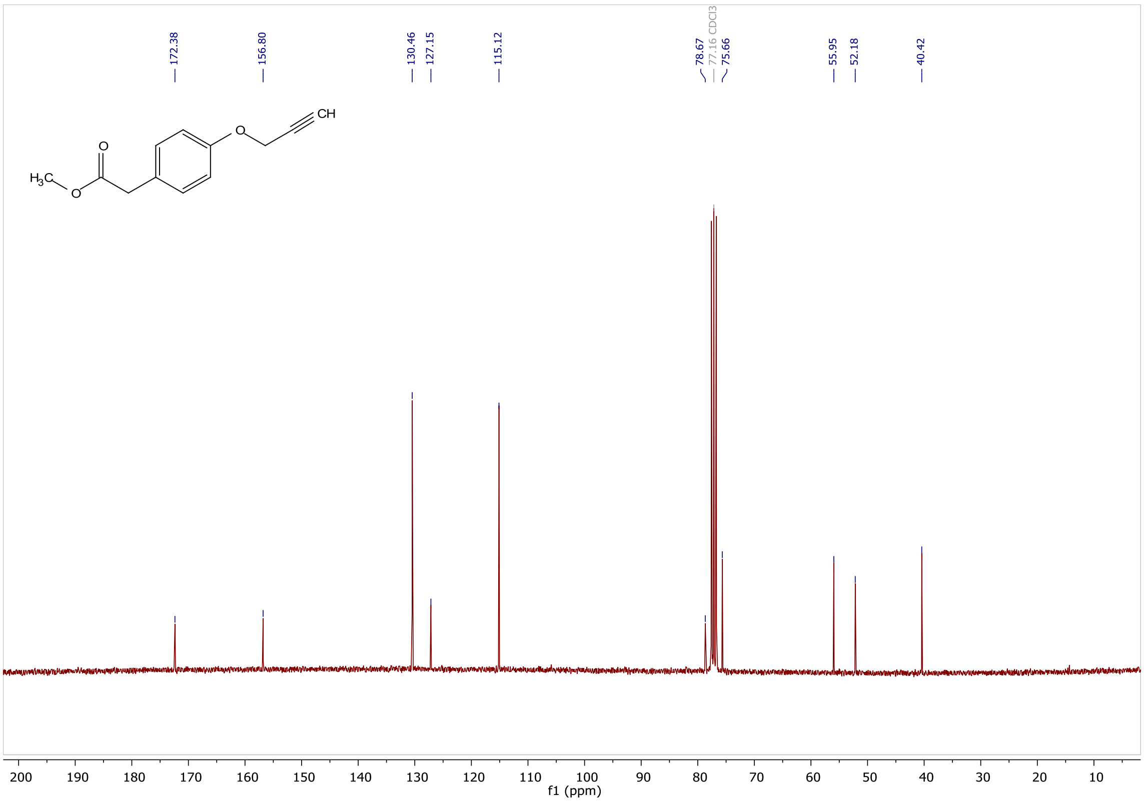

172.38
156.80
130.46
127.15
115.12
78.67
77.16 CDCl3
75.66
55.95
52.18
40.42
CH
O
O
H3C
O
200 190 180 170 160 150 140 130 120 110 100 90 80 70 60 50 40 30 20 10
f1 (ppm)

Compound 19

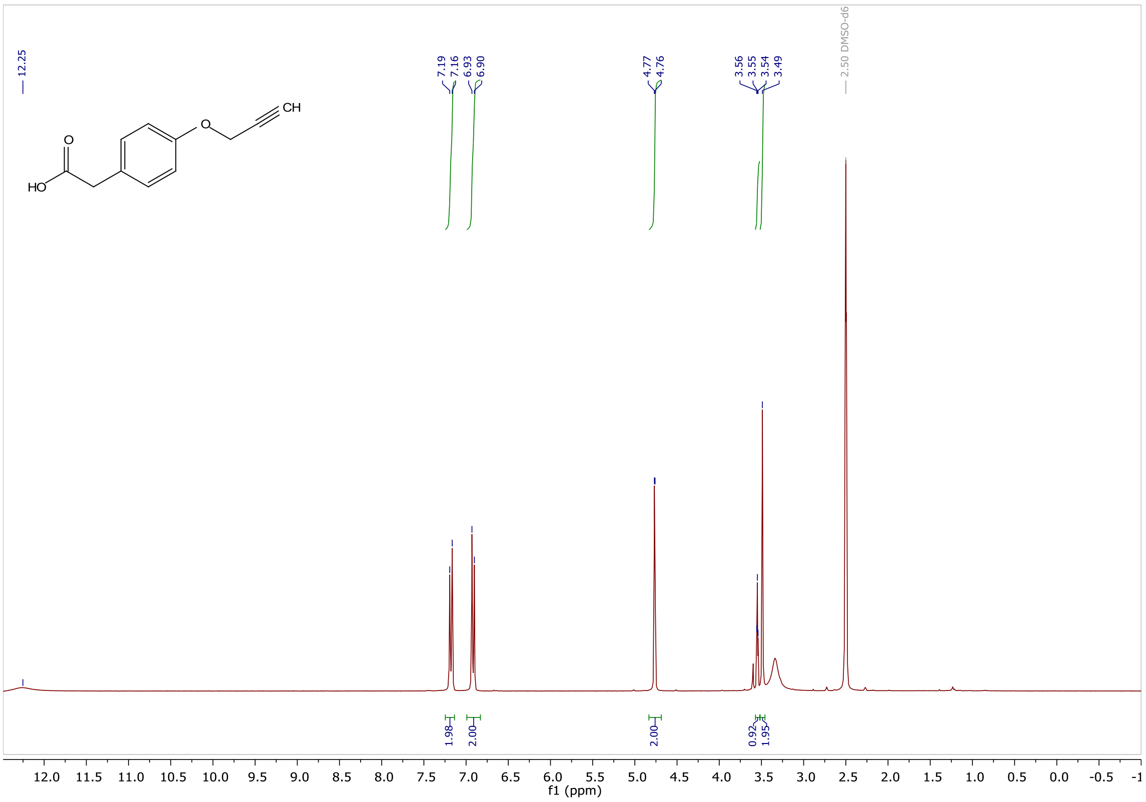

12.25
7.19
7.16
6.93
6.90
4.77
4.76
3.56
3.55
3.54
3.49
2.50 DMSO-d6
CH
O
HO
1.98
2.00
2.00
0.92
1.95
f1 (ppm)

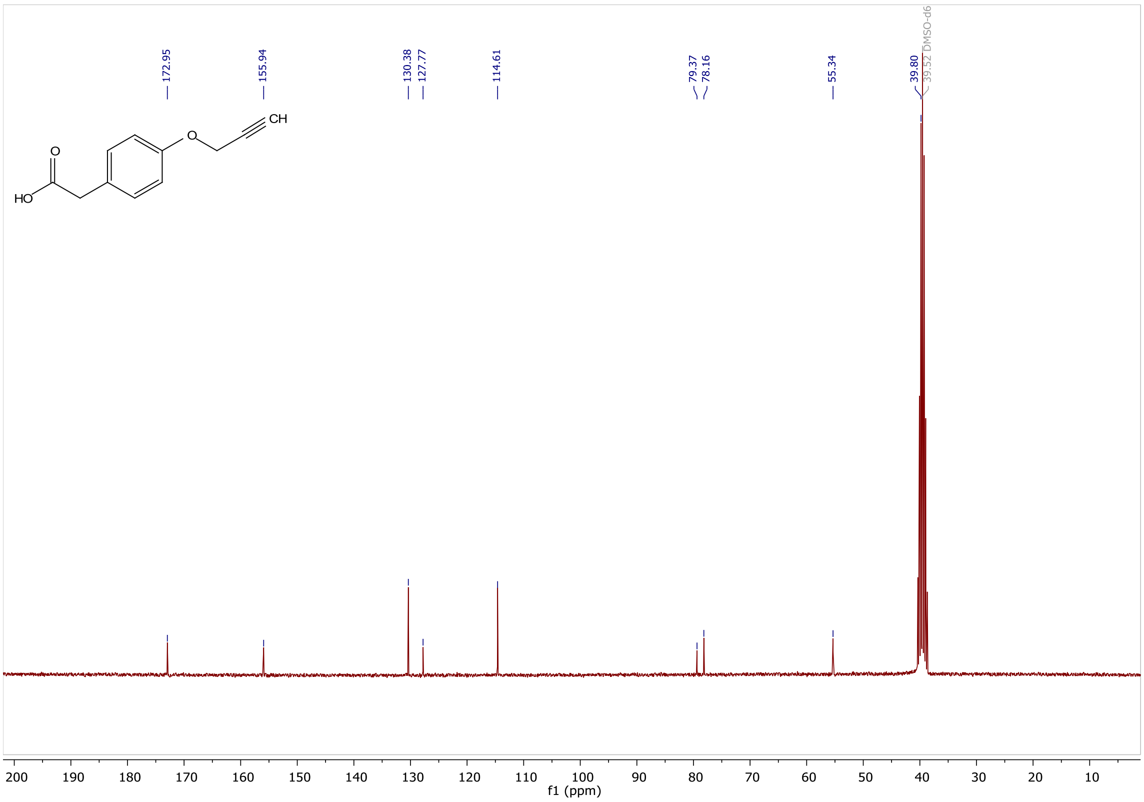

172.95
155.94
130.38
127.77
114.61
79.37
78.16
55.34
39.80
39.52 DMSO-d6
CH
O
HO
f1 (ppm)

Compound 20

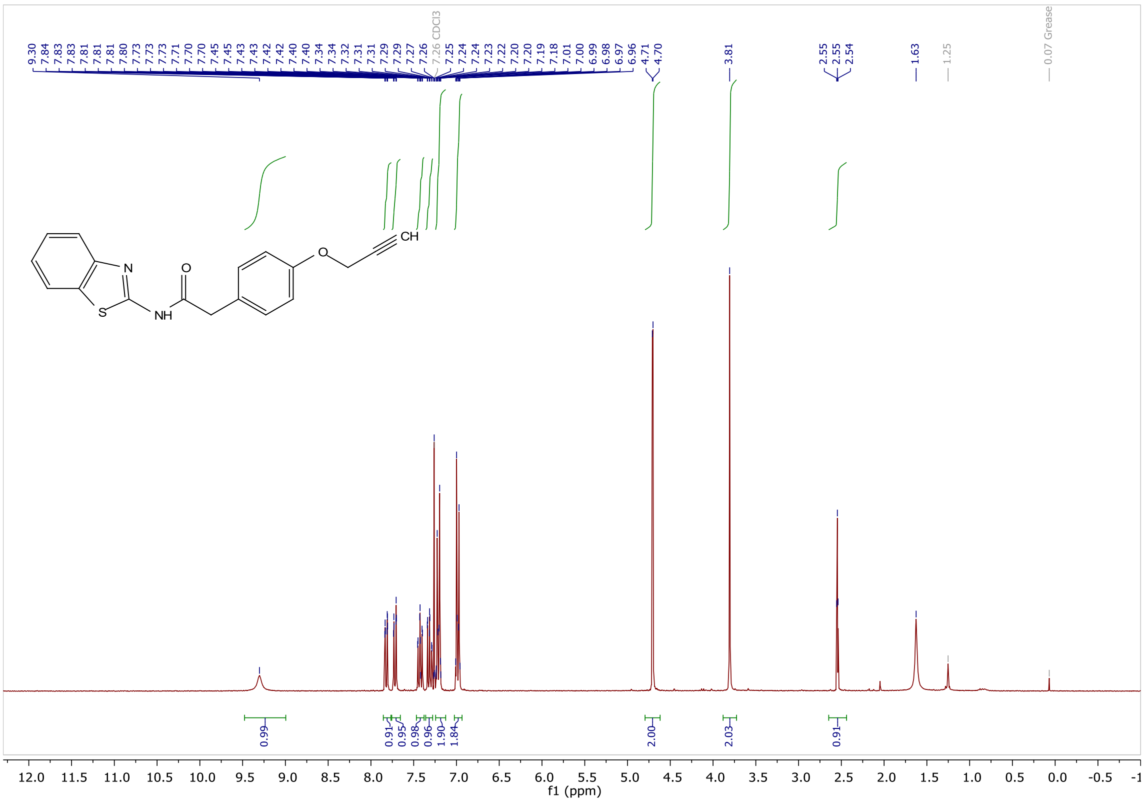

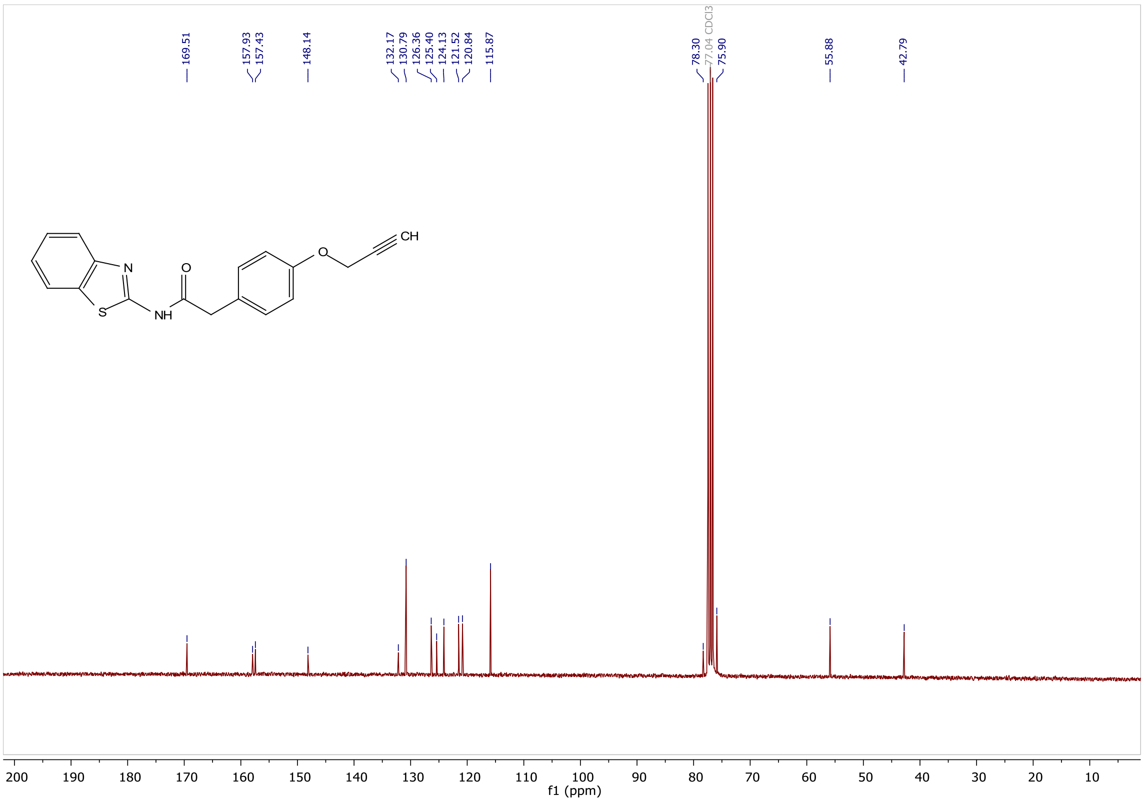

Compound 21

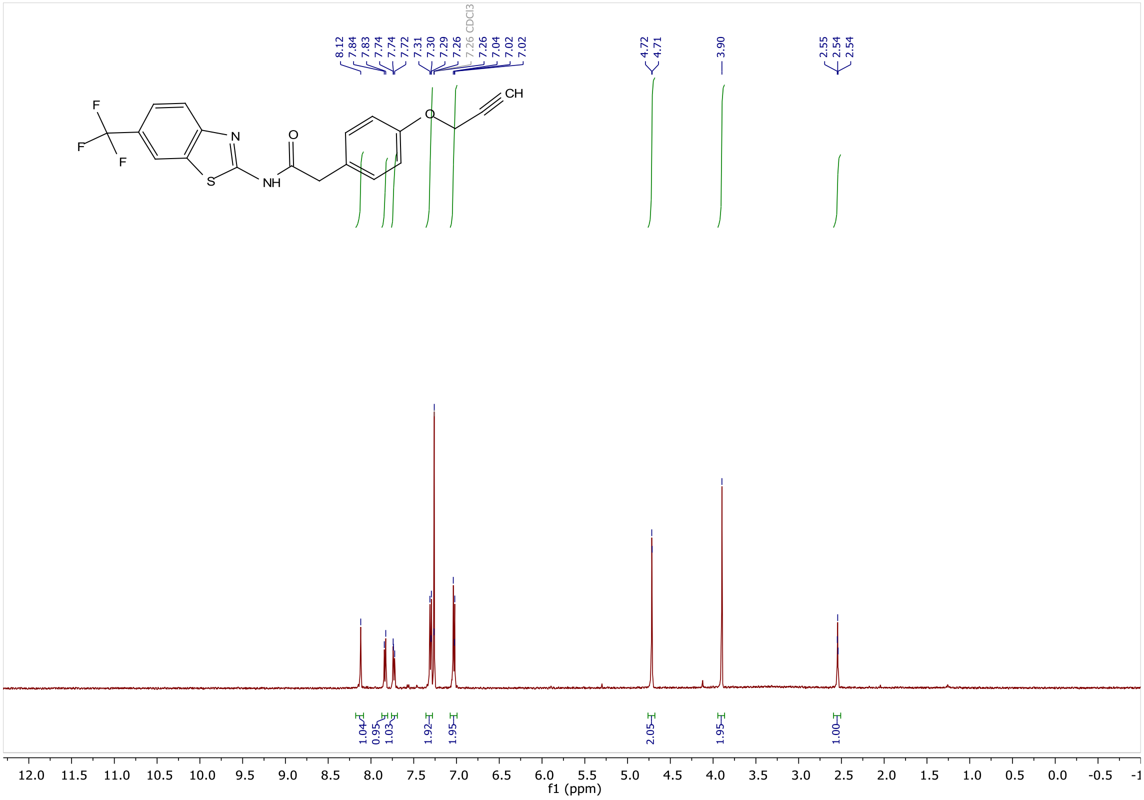

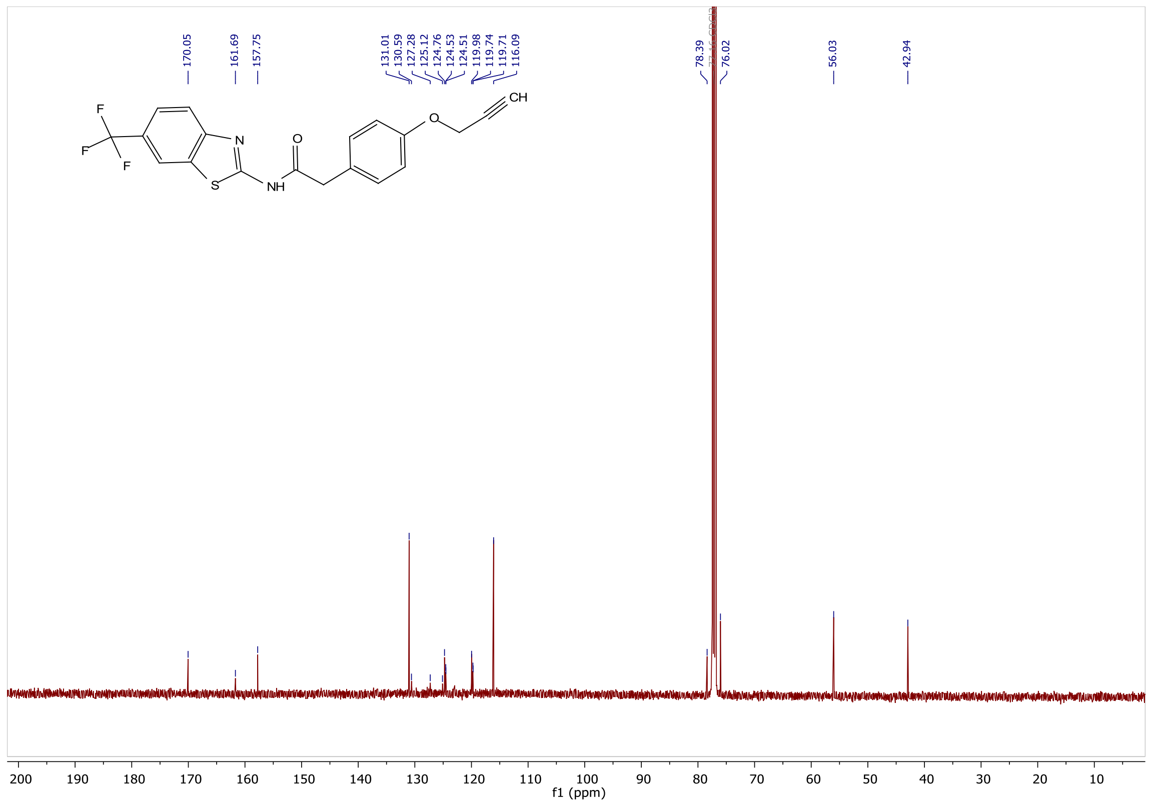
170.05
161.69
157.75
131.01
130.59
127.28
125.12
124.76
124.53
124.51
119.98
119.74
119.71
116.09
78.39
76.02
56.03
42.94
F
F
F
N
S
NH
O
O
CH
f1 (ppm)

Compound 22

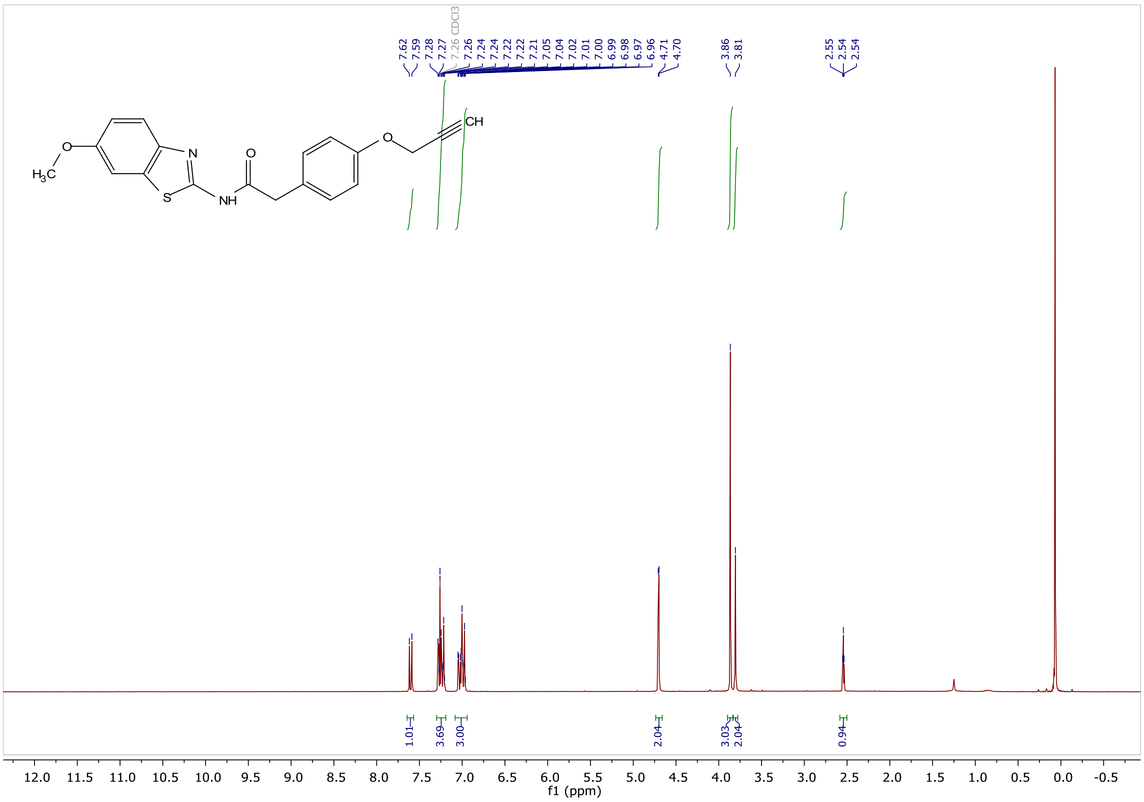

7.62
7.59
7.28
7.27
7.26 CDCl3
7.26
7.24
7.24
7.22
7.22
7.21
7.05
7.04
7.02
7.01
7.00
6.99
6.98
6.97
6.96
4.71
4.70
3.86
3.81
2.55
2.54
2.54
O
N
O
O
CH
H3C
S
NH
1.01
3.69
3.00
2.04
3.03
2.04
0.94
f1 (ppm)

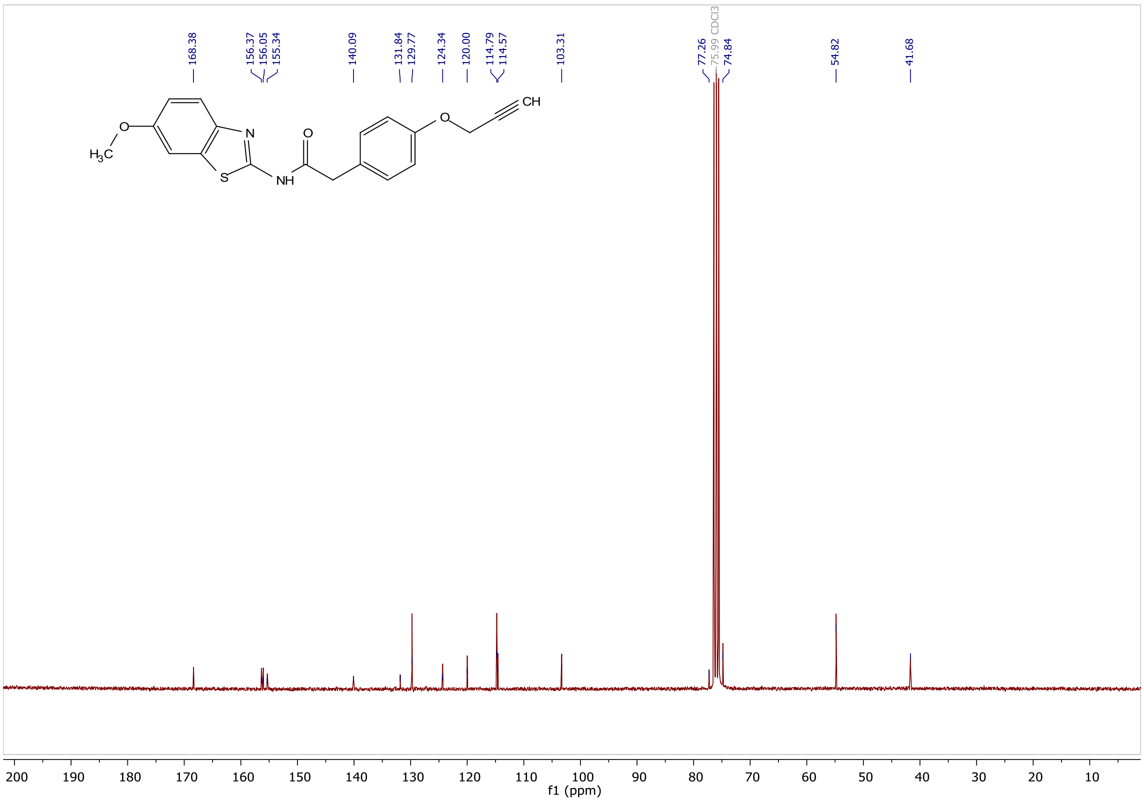

168.38
156.37
156.05
155.34
140.09
131.84
129.77
124.34
120.00
114.79
114.57
103.31
77.26
75.99 CDCl3
74.84
54.82
41.68
O
N
O
O
CH
H3C
S
NH
f1 (ppm)

Compound 23

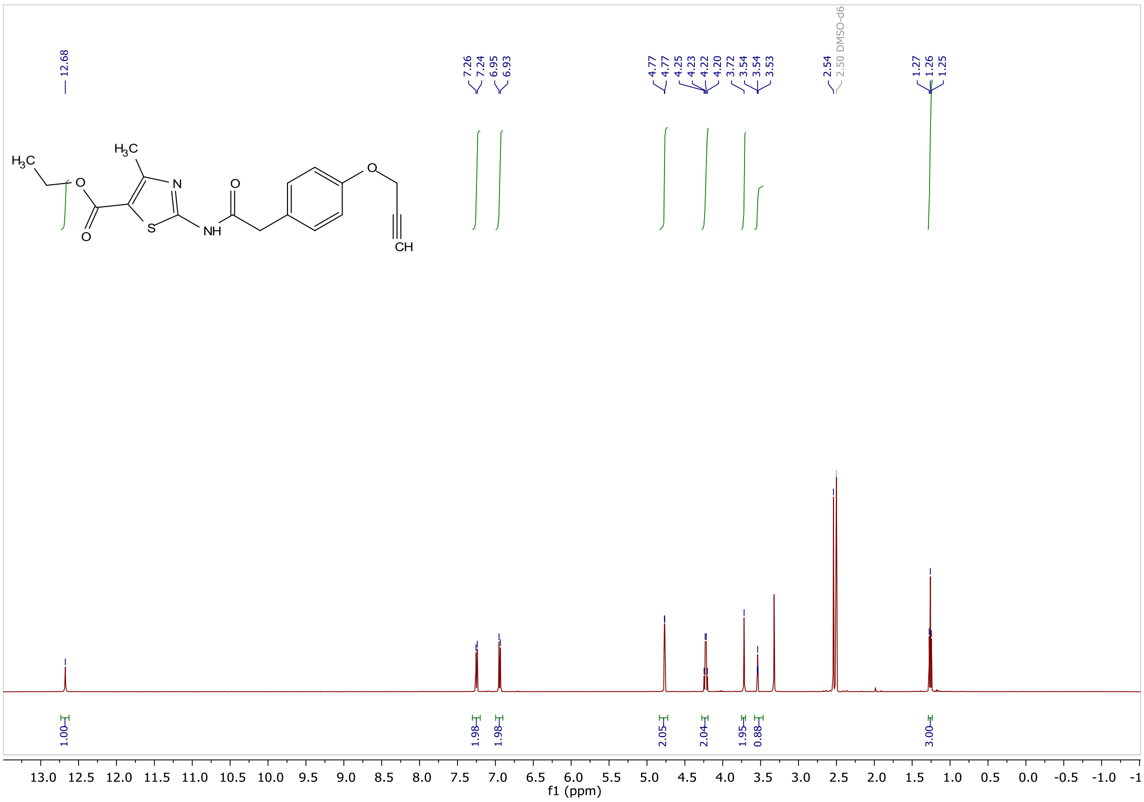

12.68
7.26
7.24
6.95
6.93
4.77
4.77
4.25
4.23
4.22
4.20
3.72
3.54
3.54
3.53
2.54
2.50 DMSO-d6
1.27
1.26
1.25
1.00
1.98
1.98
2.05
2.04
1.95
0.88
3.00
13.0 12.5 12.0 11.5 11.0 10.5 10.0 9.5 9.0 8.5 8.0 7.5 7.0 6.5 6.0 5.5 5.0 4.5 4.0 3.5 3.0 2.5 2.0 1.5 1.0 0.5 0.0 -0.5 -1.0
f1 (ppm)

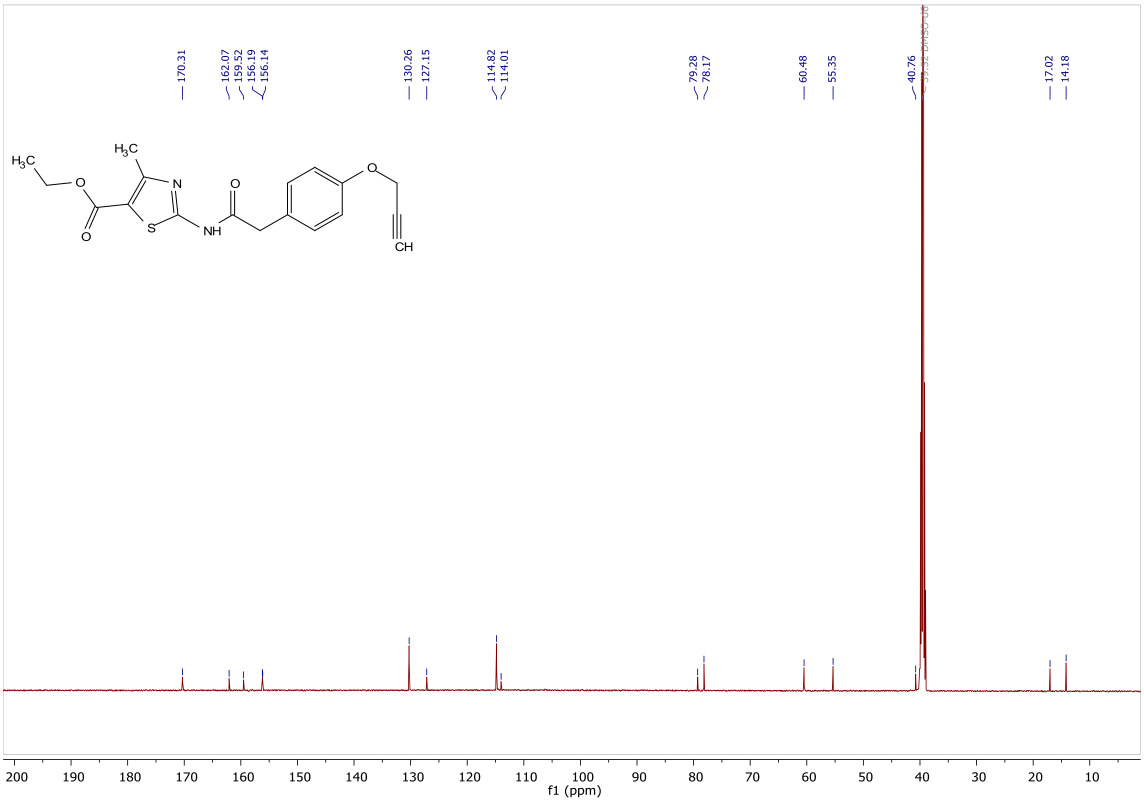

170.31
162.07
159.52
156.19
156.14
130.26
127.15
114.82
114.01
79.28
78.17
60.48
55.35
40.76
17.02
14.18
200 190 180 170 160 150 140 130 120 110 100 90 80 70 60 50 40 30 20 10
f1 (ppm)

Compound 25

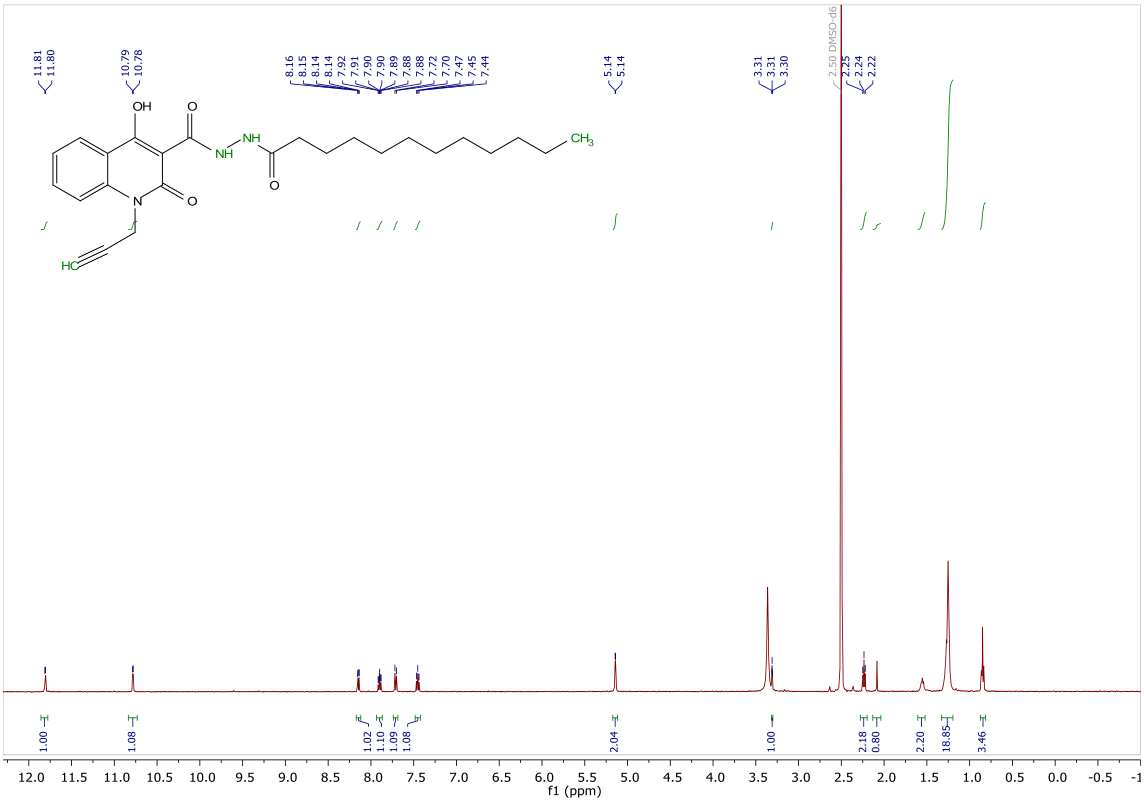
11.81
11.80
10.79
10.78
8.16
8.15
8.14
8.14
7.92
7.91
7.90
7.90
7.89
7.88
7.88
7.72
7.70
7.47
7.45
7.44
5.14
5.14
3.31
3.31
3.30
2.50 DMSO-d6
2.25
2.24
2.22
OH
O
NH
NH
O
CH3
N
O
HC
1.00
1.08
1.02
1.10
1.09
1.08
2.04
1.00
2.18
0.80
2.20
18.85
3.46
f1 (ppm)

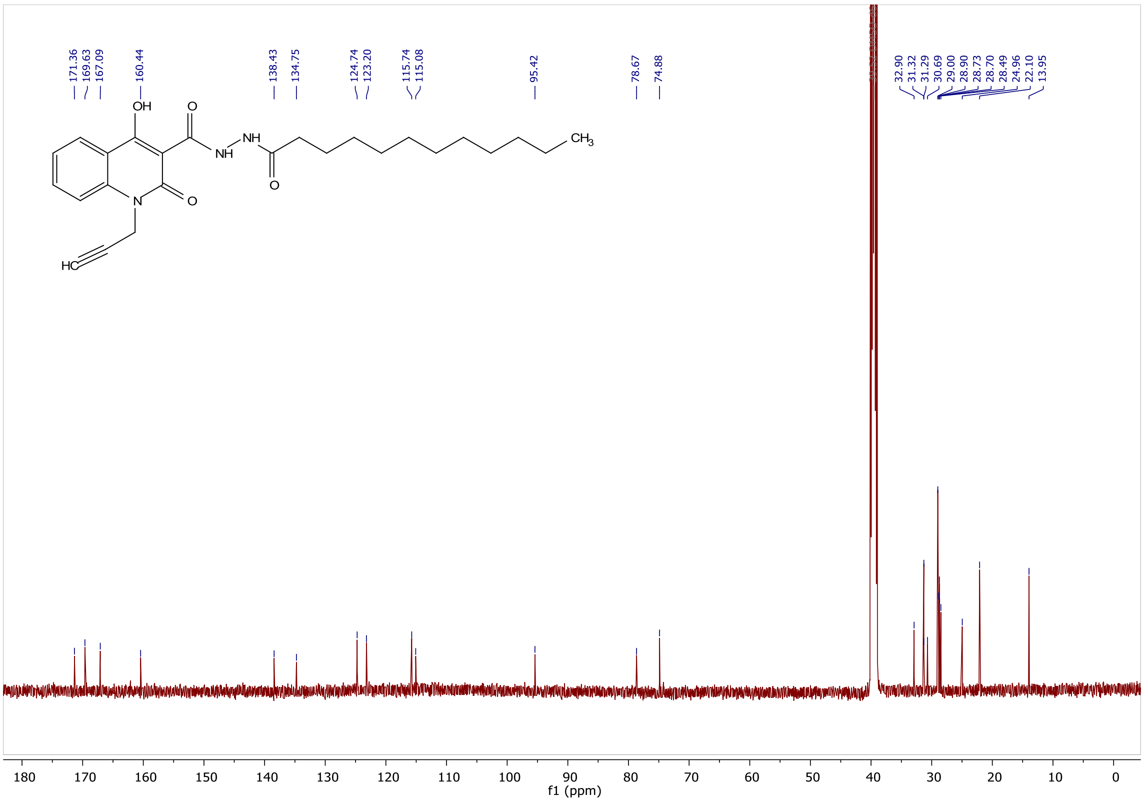
171.36
169.63
167.09
160.44
138.43
134.75
124.74
123.20
115.74
115.08
95.42
78.67
74.88
32.90
31.32
31.29
30.69
29.00
28.90
28.73
28.70
28.49
24.96
22.10
13.95
OH
O
NH
NH
O
CH3
N
O
HC
f1 (ppm)

Compound 27

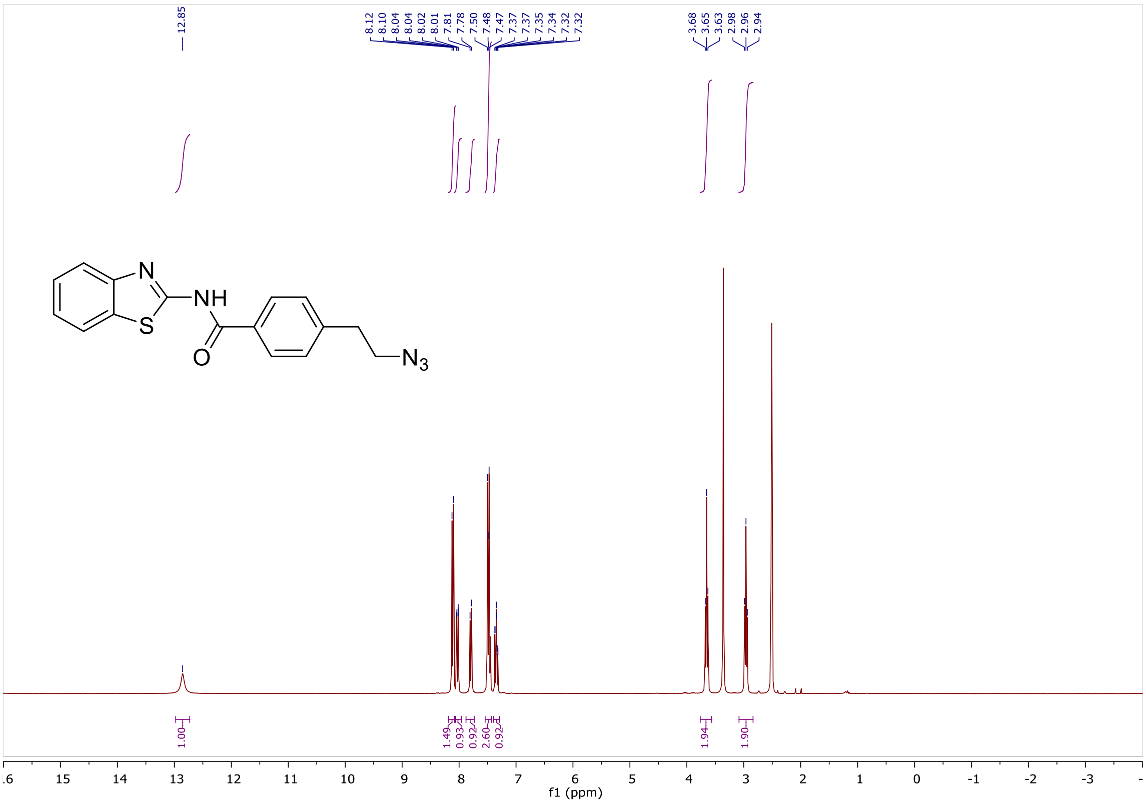

12.85
8.12
8.10
8.04
8.04
8.02
8.01
7.81
7.78
7.50
7.48
7.47
7.37
7.37
7.35
7.34
7.32
7.32
3.68
3.65
3.63
2.98
2.96
2.94
N
NH
S
O
N3
1.00
1.49
0.93
0.92
2.60
0.92
1.94
1.90
f1 (ppm)

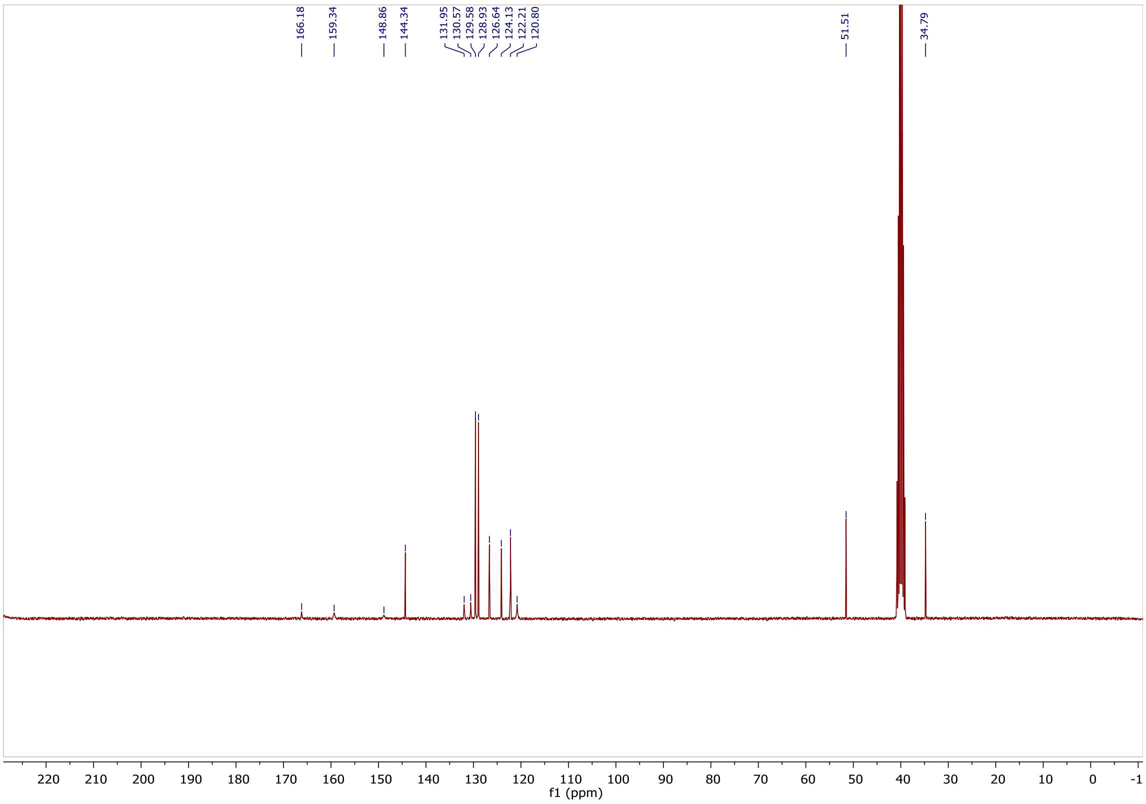

166.18
159.34
148.86
144.34
131.95
130.57
129.58
128.93
126.64
124.13
122.21
120.80
51.51
34.79
f1 (ppm)

Compound 28

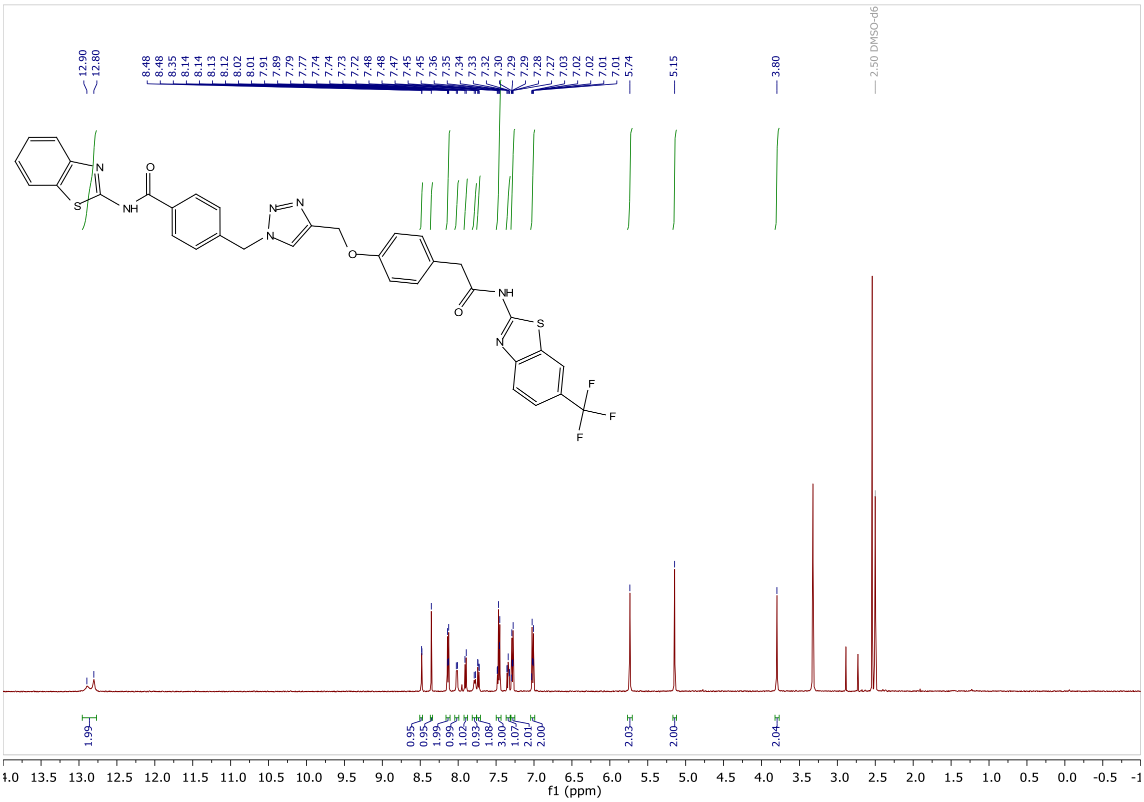
12.90
12.80
8.48
8.48
8.35
8.14
8.14
8.13
8.12
8.02
8.01
7.91
7.89
7.79
7.77
7.74
7.74
7.73
7.72
7.48
7.48
7.47
7.45
7.45
7.36
7.35
7.34
7.33
7.32
7.30
7.29
7.29
7.28
7.27
7.03
7.02
7.02
7.01
7.01
5.74
5.15
3.80
2.50 DMSO-d6
1.99
0.95
0.95
1.99
0.99
1.02
0.93
1.08
3.00
1.07
2.01
2.00
2.03
2.00
2.04
f1 (ppm)

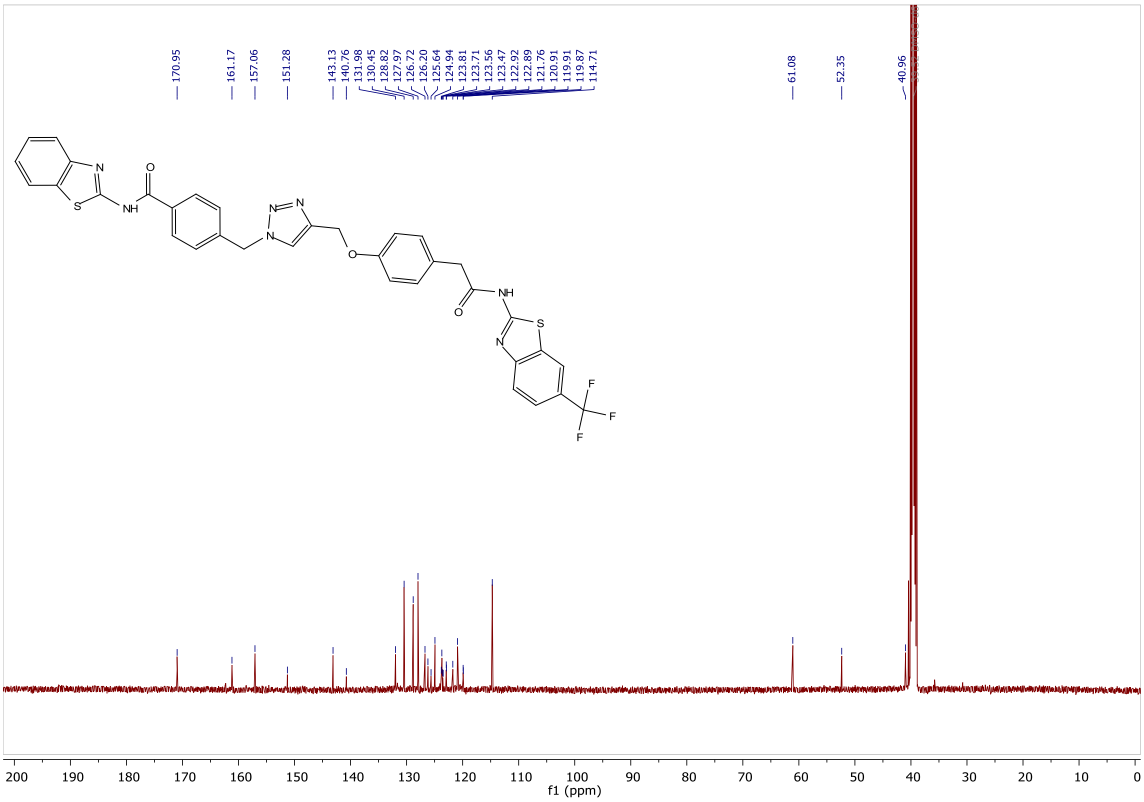
170.95
161.17
157.06
151.28
143.13
140.76
131.98
130.45
128.82
127.97
126.72
126.20
125.64
124.94
123.81
123.71
123.56
123.47
122.92
122.89
121.76
120.91
119.91
119.87
114.71
61.08
52.35
40.96
f1 (ppm)

Compound 29

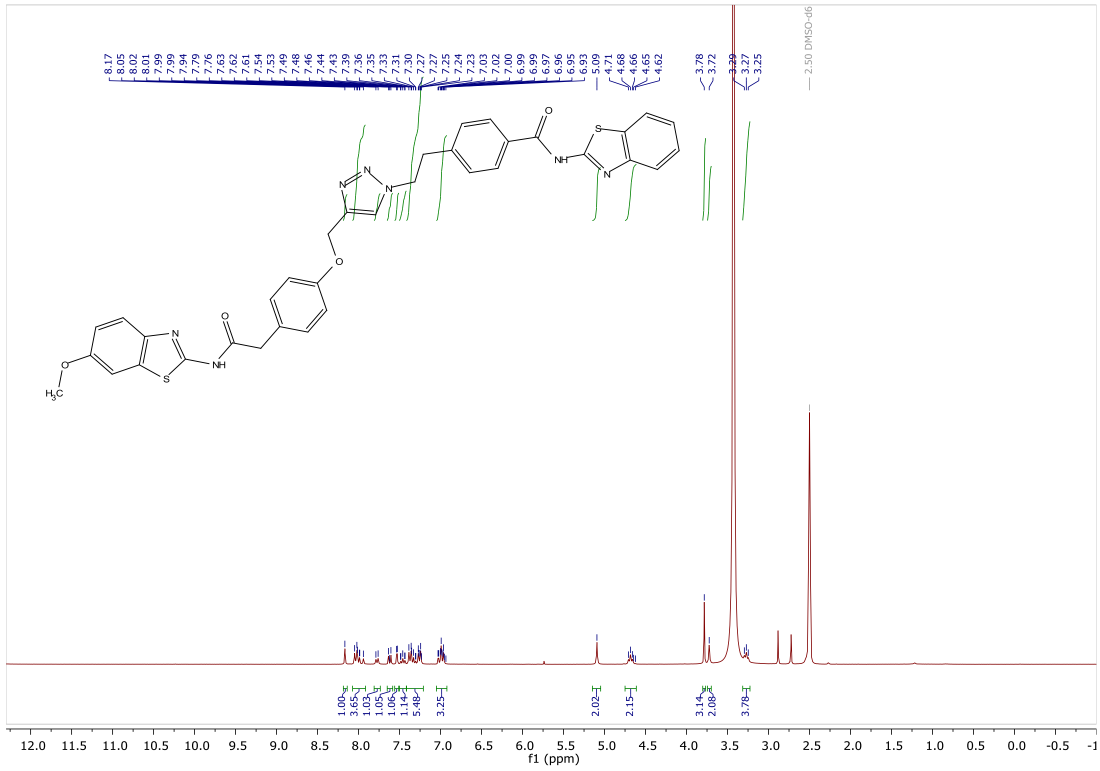

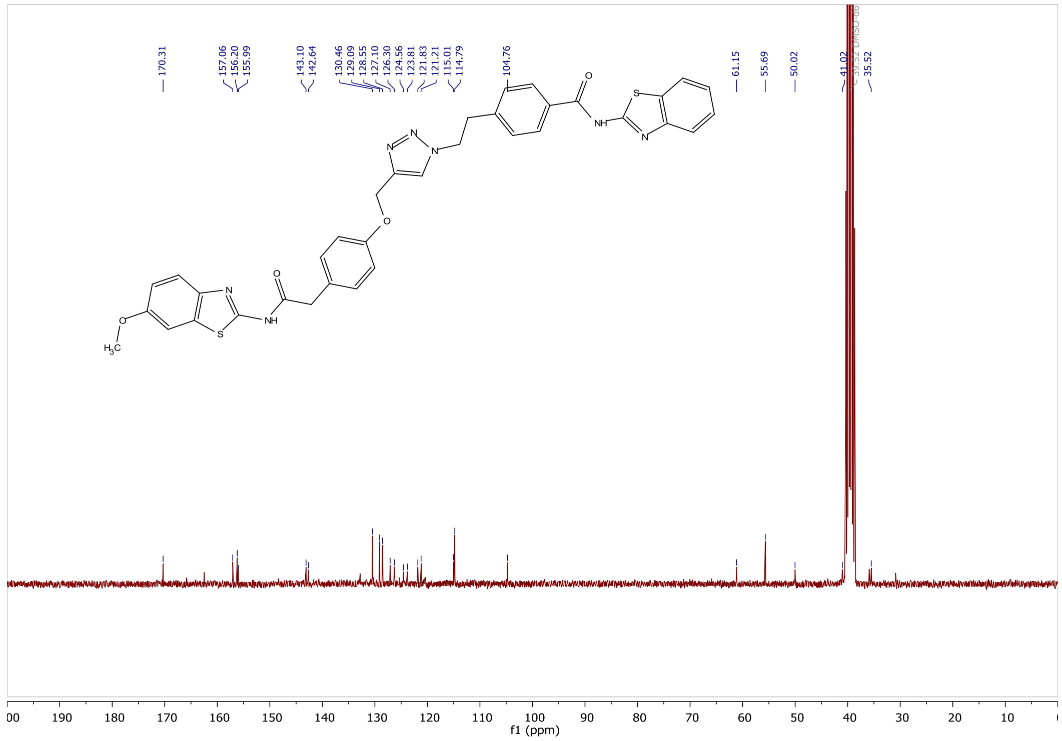

Compound 30

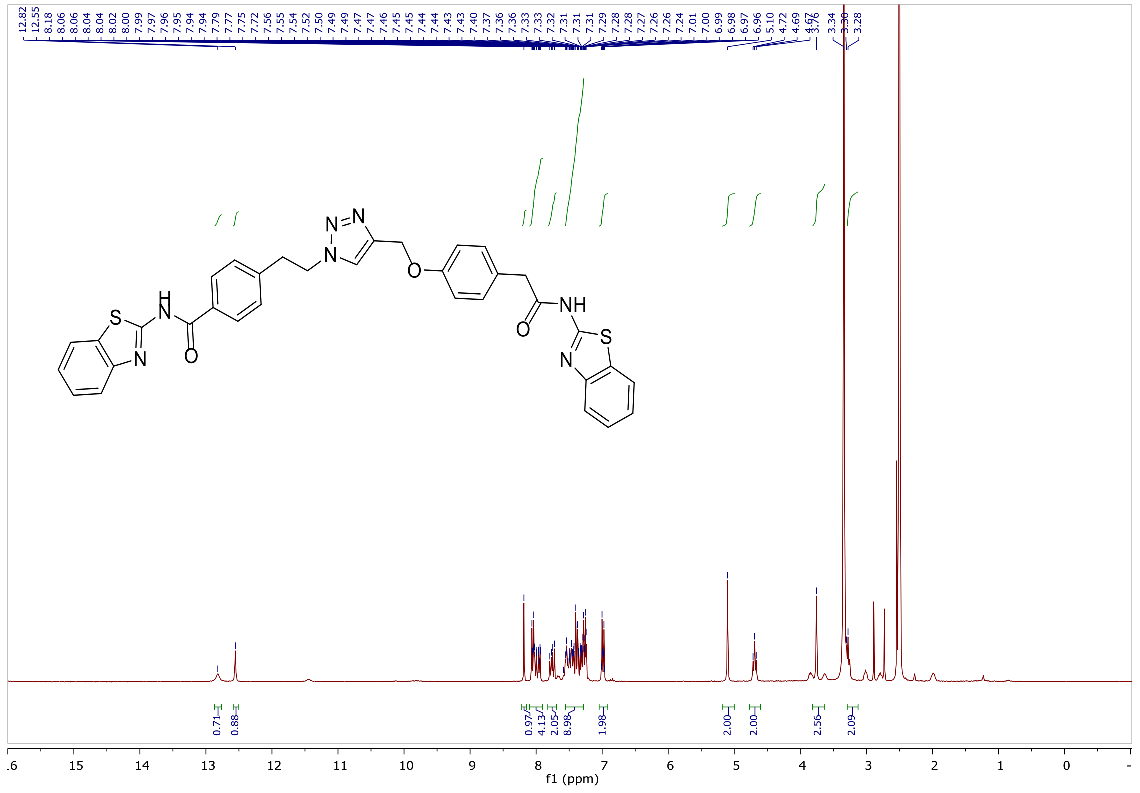

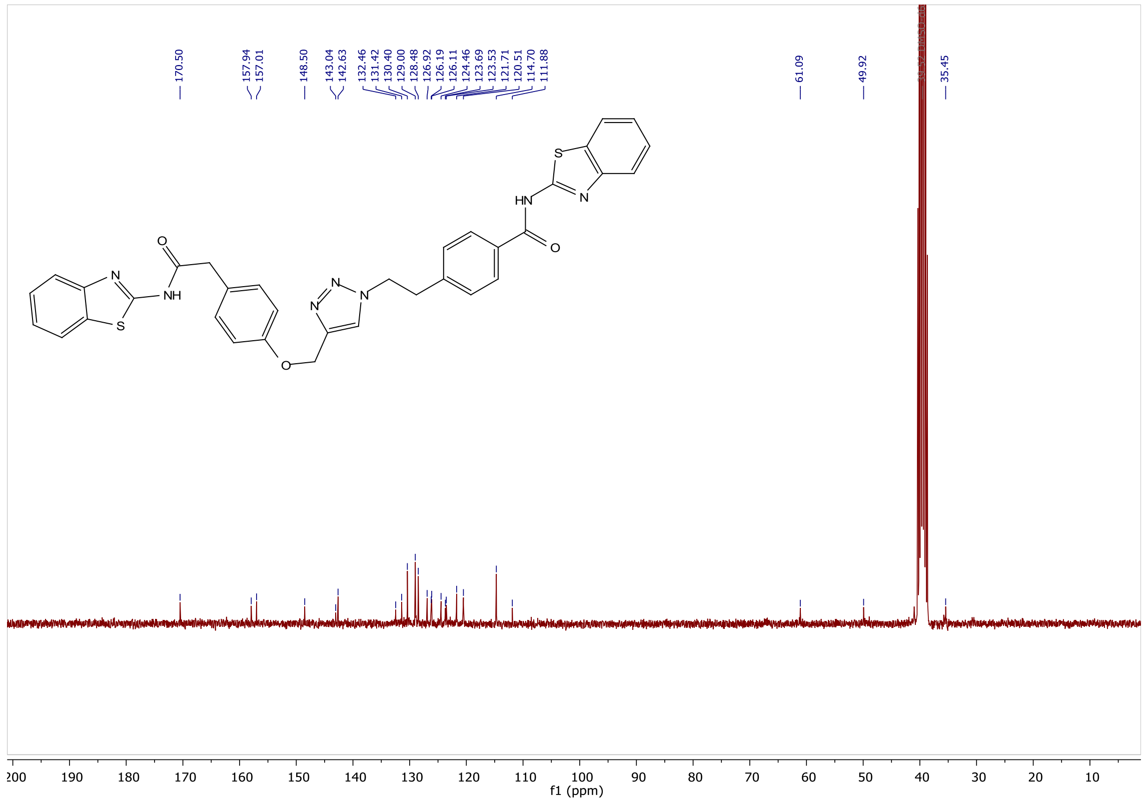

Compound 31

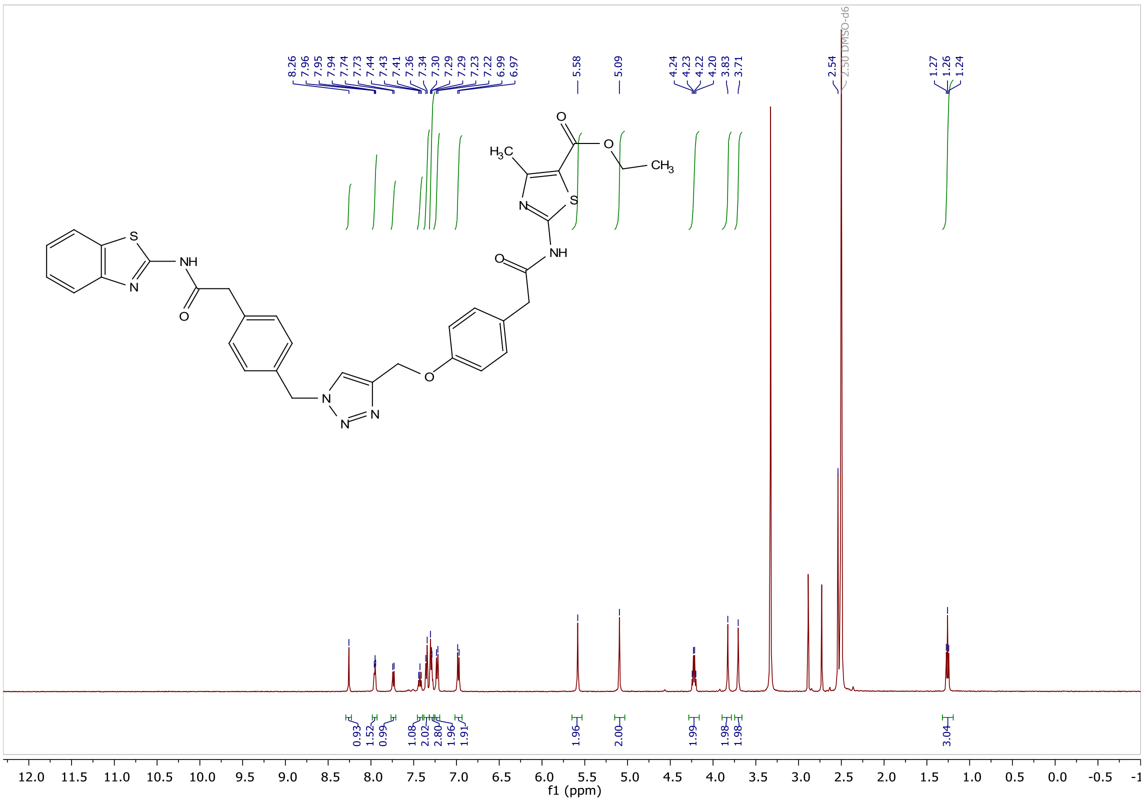
8.26 7.96 7.95 7.94 7.74 7.73 7.44 7.43 7.41 7.36 7.34 7.30 7.29 7.29 7.23 7.22 6.99 6.97
5.58
5.09
4.24 4.23 4.22 4.20 3.83 3.71
2.54
2.50 DMSO-d6
1.27 1.26 1.24
H3C
O
O
CH3
S
N
NH
O
S
NH
N
O
O
N
N
N
0.93 1.52 0.99 1.08 2.02 2.80 1.96 1.91
1.96
2.00
1.99
1.98 1.98
3.04
f1 (ppm)

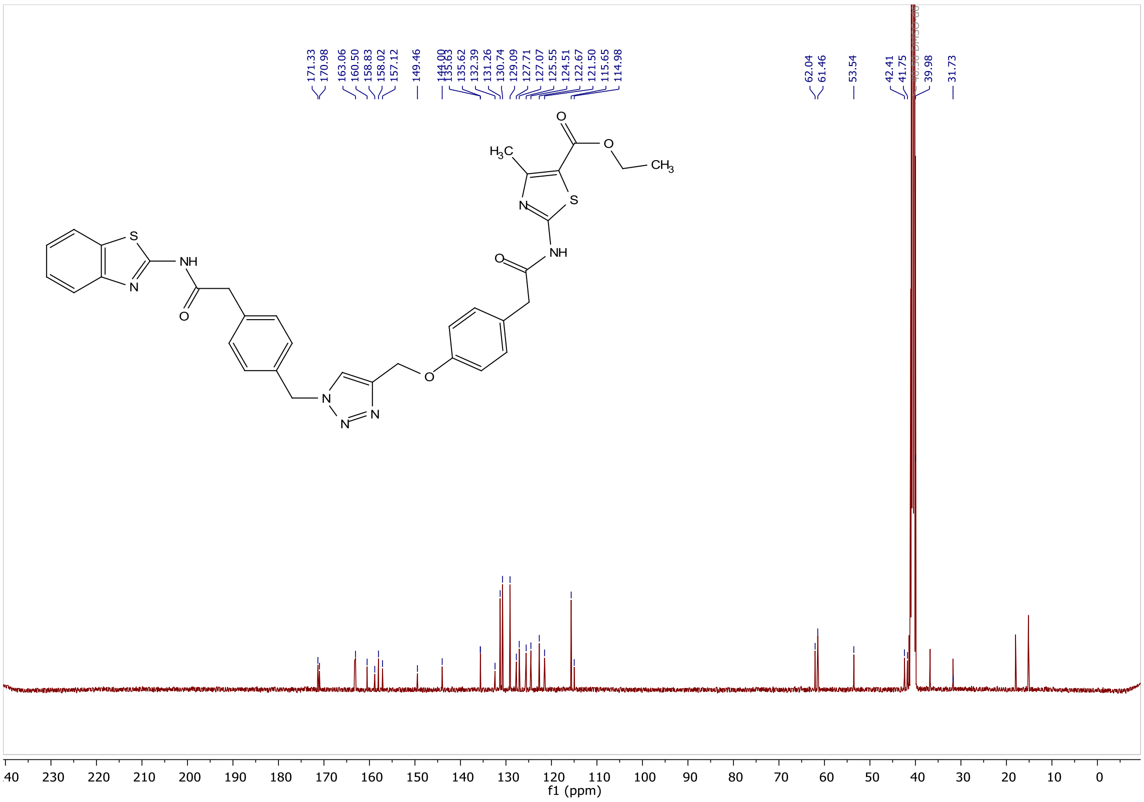
171.33 170.98 163.06 160.50 158.83 158.02 157.12
149.46
144.00 [illegible] 135.62 132.39 131.26 130.74 129.09 127.71 127.07 125.55 124.51 122.67 121.50 115.65 114.98
62.04 61.46
53.54
42.41 41.75
39.98
31.73
H3C
O
O
CH3
S
N
NH
O
S
NH
N
O
O
N
N
N
f1 (ppm)

Compound 32

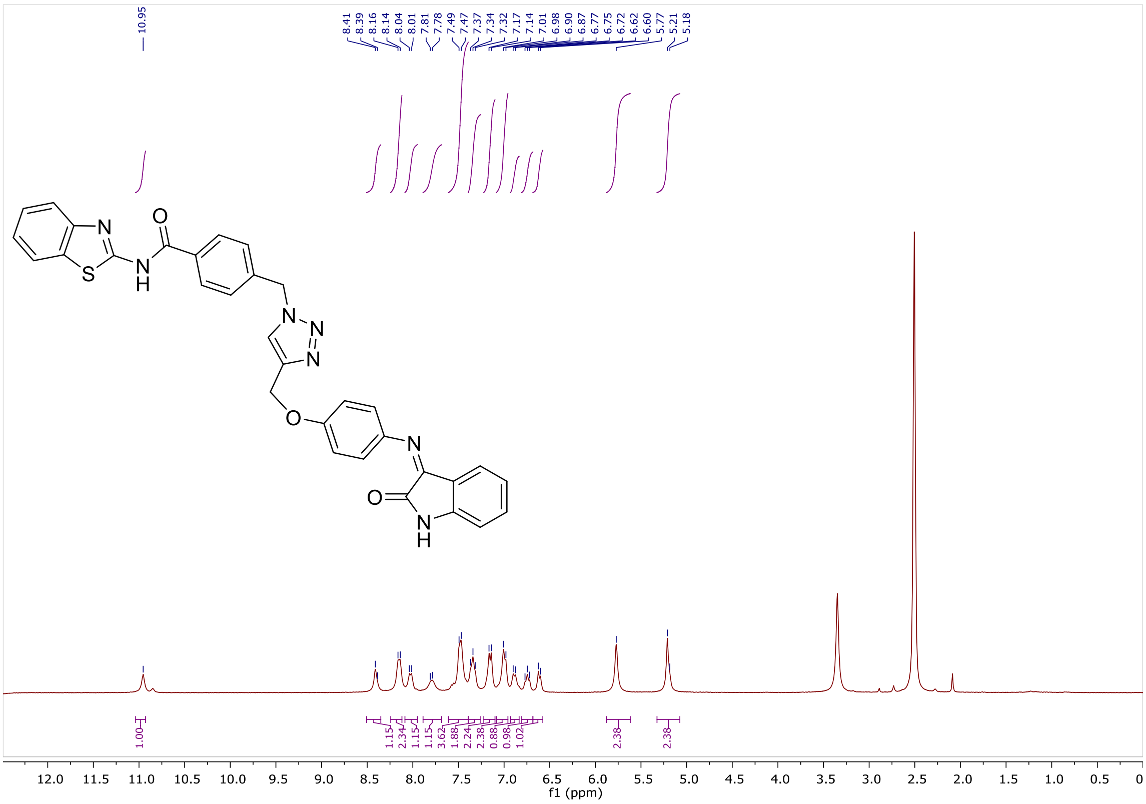

10.95
8.41
8.39
8.16
8.14
8.04
8.01
7.81
7.78
7.49
7.47
7.37
7.34
7.32
7.17
7.14
7.01
6.98
6.90
6.87
6.77
6.75
6.72
6.62
6.60
5.77
5.21
5.18
1.00
1.15
2.34
1.15
1.15
3.62
1.88
2.24
2.38
0.88
0.98
1.02
2.38
2.38
f1 (ppm)

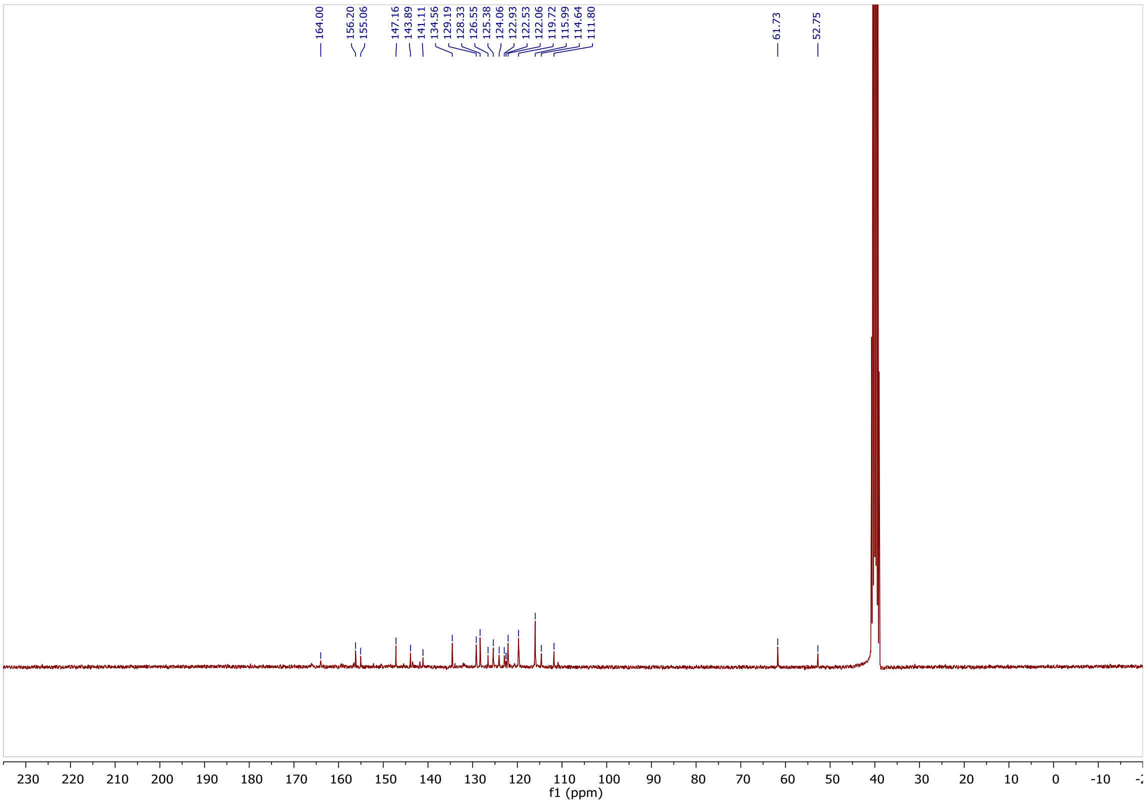

164.00
156.20
155.06
147.16
143.89
141.11
134.56
129.19
128.33
126.55
125.38
124.06
122.93
122.53
122.06
119.72
115.99
114.64
111.80
61.73
52.75
f1 (ppm)

Compound 33

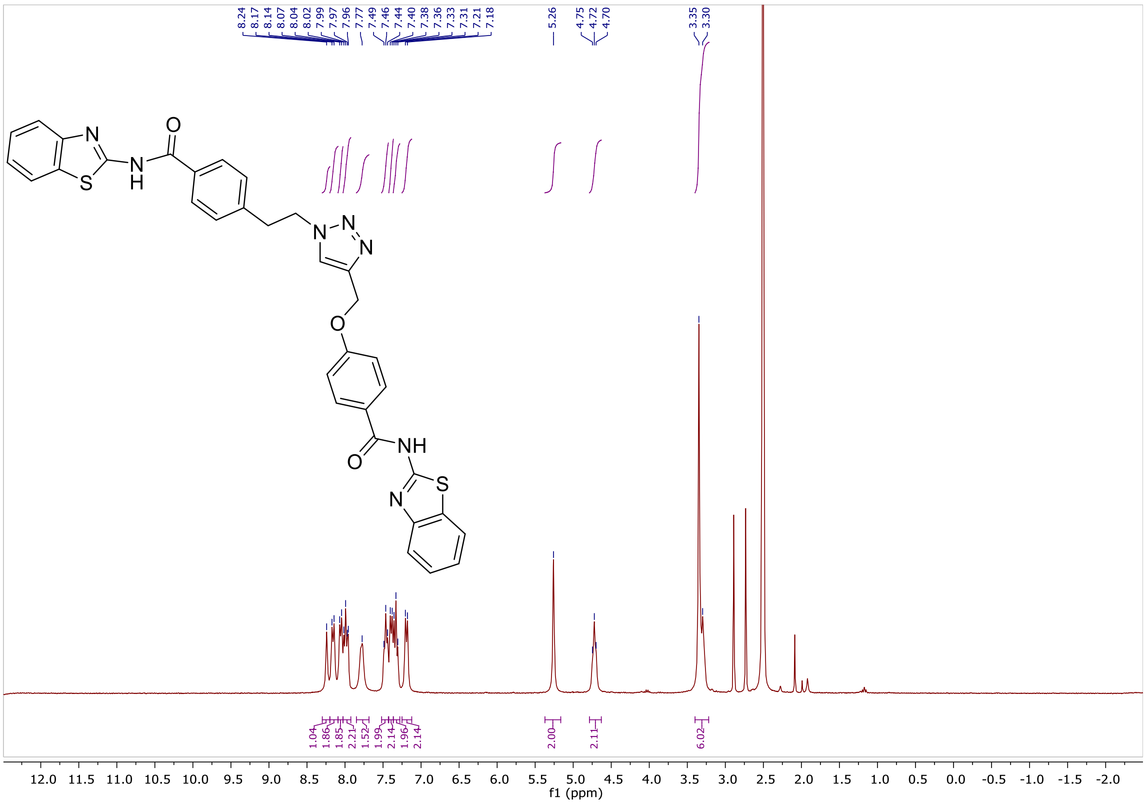
8.24
8.17
8.14
8.07
8.04
8.02
7.99
7.97
7.96
7.77
7.49
7.46
7.44
7.40
7.38
7.36
7.33
7.31
7.21
7.18
5.26
4.75
4.72
4.70
3.35
3.30
1.04
1.86
1.85
2.21
1.52
1.99
2.14
1.96
2.14
2.00
2.11
6.02
f1 (ppm)

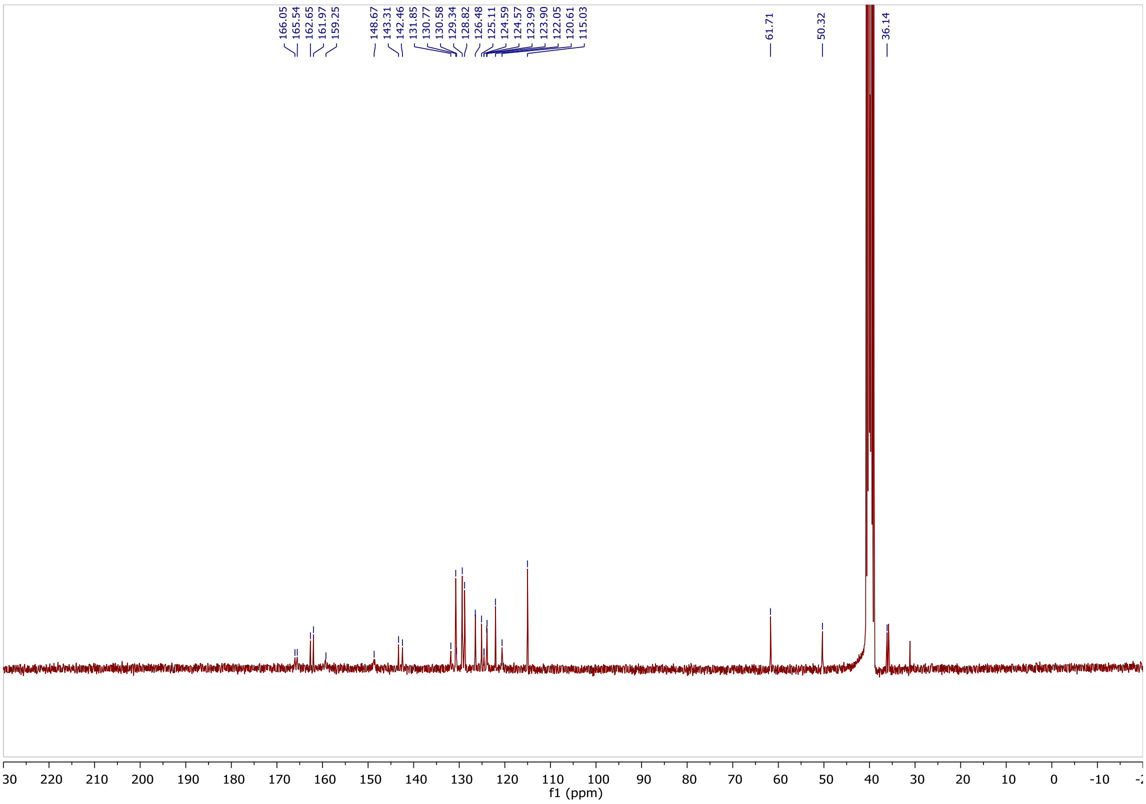
166.05
165.54
162.65
161.97
159.25
148.67
143.31
142.46
131.85
130.77
130.58
129.34
128.82
126.48
125.11
124.59
124.57
123.99
123.90
122.05
120.61
115.03
61.71
50.32
36.14
f1 (ppm)

Table S3. Permeability prediction (Pe 10-6 cm s-1) in the PAMPA-BBB assay for the commercial drugs used as validation and compounds with their predictive penetration in the CNS. Results are expressed as the mean (standard deviation) of two independent runs.

**Compound**	**Bibl.**	**Pe exp (10-6 cm s-1)**	**CNS prediction**
Atenolol	0.8	0.60± 0.47	
Caffeine	1.3	1.45 ± 0.26	
Desipramine	12	7.44 ± 0.9	
Enoxacine	0.9	1.41 ± 0.05	
Ofloxacine	0.8	1.74 ± 0.04	
Piroxicam	2.5	2.92 ± 0.37	
Promazine	8.8	5.18 ± 0.43	
Testosterone	17	13.36 ± 1.06	
Verapamile	16	10.99 ± 1.47	
14		-	CNS-
15		5.08 ± 0.63	CNS+
16		3.07 ± 0.25	CNS+/CNS-
28		0.65± 0.18	CNS-
29		-	CNS-
30		3.58± 0.12	CNS+

Figure S14. Linear correlation among experimental and reported permeability of commercial drug using the PAMPA-BBB assay.

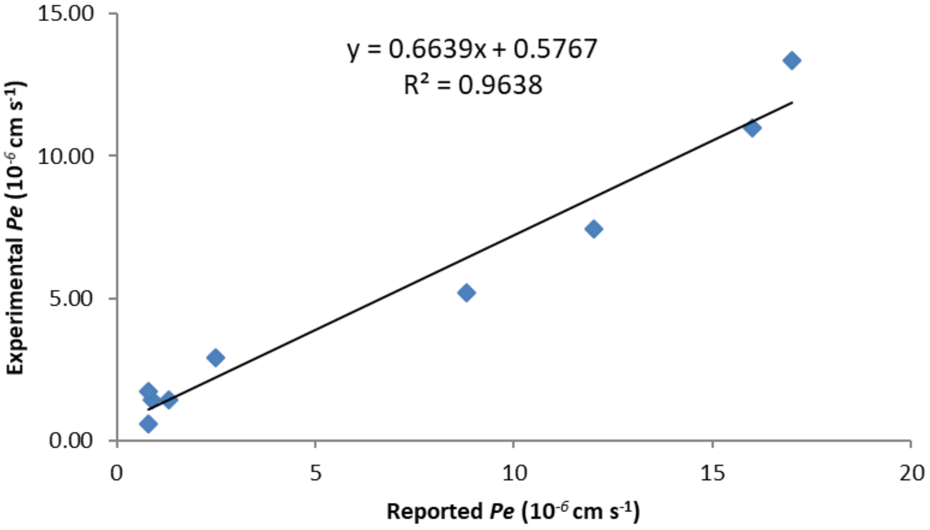

Supplementary bibliography

1. Di, L.; Kerns, E. H.; Fan, K.; McConnell, O. J.; Carter, G. T., High throughput artificial membrane permeability assay for blood-brain barrier. *Eur J Med Chem* **2003,** *38* (3), 223-32.
2. Crivori, P.; Cruciani, G.; Carrupt, P. A.; Testa, B., Predicting blood-brain barrier permeation from three-dimensional molecular structure. *J Med Chem* **2000,** *43* (11), 2204-16.
3. Lesnik, S.; Stular, T.; Brus, B.; Knez, D.; Gobec, S.; Janezic, D.; Konc, J., LiSiCA: A Software for Ligand-Based Virtual Screening and Its Application for the Discovery of Butyrylcholinesterase Inhibitors. *J Chem Inf Model* **2015,** *55* (8), 1521-8.
4. Jordan, J. B.; Whittington, D. A.; Bartberger, M. D.; Sickmier, E. A.; Chen, K.; Cheng, Y.; Judd, T., Fragment-Linking Approach Using (19)F NMR Spectroscopy To Obtain Highly Potent and Selective Inhibitors of beta-Secretase. *J Med Chem* **2016,** *59* (8), 3732-49.
5. Friesner, R. A.; Murphy, R. B.; Repasky, M. P.; Frye, L. L.; Greenwood, J. R.; Halgren, T. A.; Sanschagrin, P. C.; Mainz, D. T., Extra precision glide: docking and scoring incorporating a model of hydrophobic enclosure for protein-ligand complexes. *J Med Chem* **2006,** *49* (21), 6177-96.
6. Jacobson, M. P.; Pincus, D. L.; Rapp, C. S.; Day, T. J.; Honig, B.; Shaw, D. E.; Friesner, R. A., A hierarchical approach to all-atom protein loop prediction. *Proteins* **2004,** *55* (2), 351-67.
7. Ramirez, D.; Concha, G.; Arevalo, B.; Prent-Penaloza, L.; Zuniga, L.; Kiper, A. K.; Rinne, S.; Reyes-Parada, M.; Decher, N.; Gonzalez, W.; Caballero, J., Discovery of Novel TASK-3 Channel Blockers Using a Pharmacophore-Based Virtual Screening. *Int J Mol Sci* **2019,** *20* (16).
8. Ramirez, D.; Bedoya, M.; Kiper, A. K.; Rinne, S.; Morales-Navarro, S.; Hernandez-Rodriguez, E. W.; Sepulveda, F. V.; Decher, N.; Gonzalez, W., Structure/Activity Analysis of TASK-3 Channel Antagonists Based on a 5,6,7,8 tetrahydropyrido[4,3-d]pyrimidine. *Int J Mol Sci* **2019,** *20* (9).
9. Ramirez, D.; Arevalo, B.; Martinez, G.; Rinne, S.; Sepulveda, F. V.; Decher, N.; Gonzalez, W., Side Fenestrations Provide an "Anchor" for a Stable Binding of A1899 to the Pore of TASK-1 Potassium Channels. *Mol Pharm* **2017,** *14* (7), 2197-2208.
10. Bowers K.J.; Chow, E.; Xu, H.; Dror, R.O.; Eastwood, M.P.; Gregersen, B.A.; Klepeis, J.L.; Kolossvary, I.; Moraes, M.A.; Sacerdoti, F.D.; Salmon, J.K.; Shan, Y.; Shaw, D.E. Scalable Algorithms

for Molecular Dynamics Simulations on Commodity Clusters. *Proceedings of the ACM/IEEE Conference on Supercomputing (SC06), Tampa, Florida*, **2006**, November 11-17

11. Roos K, Wu C, Damm W, Reboul M, Stevenson JM, Lu C, Dahlgren MK, Mondal S, Chen W, Wang L, Abel R, Friesner RA, Harder ED. OPLS3e: Extending Force Field Coverage for Drug-Like Small Molecules. *J Chem Theory Comput.* **2019,** *15*(3),1863-1874.

12. Martyna, G.J.; Tobias, D.J.; Klein, M.L. Constant pressure molecular dynamics algorithms. *J. Chem. Phys.* **1994,** *101,* 4177-4189.

13. Mauricio Bedoya, & Francisco Adasme. (2020, October 14). Contact surface analyzer: v1.0 (Version 1.0). Zenodo. http://doi.org/10.5281/zenodo.4088995
